# An Overview of Reviews on Telemedicine and Telehealth in Dementia Care: Mixed Methods Synthesis

**DOI:** 10.2196/75266

**Published:** 2025-11-06

**Authors:** Israel Júnior Borges do Nascimento, Hebatullah Mohamed Abdulazeem, Ishanka Weerasekara, Amin Sharifan, Victor Grandi Bianco, Indunil Kularathne, Ciara Cunningham, Brijesh Sathian, Genevieve Deeken, Lasse Østengaard, Rachel Frederique-Djurdjevic, Joost van Hoof, Ledia Lazeri, Cassie Redlich, Hannah R Marston, Nathalia Sernizon Guimarães, Jerome de Barros, Ryan Alistar dos Santos, Natasha Azzopardi-Muscat, Yongjie Yon, David Novillo-Ortiz

**Affiliations:** 1 Division of Country Health Policies and Systems, World Health Organization Regional Office for Europe, Marmorvej 51, Copenhagen, 2100, Denmark, 45 45337198; 2 Center for Research in Epidemiology and Statistics (CRESS-U1153), Université Paris Cité and Université Sorbonne Paris Nord, INRAE, Inserm, Hôpital Hôtel-Dieu, 1, Paris, France; 3 Department of Internal Medicine, School of Medicine and Hospital Universitário Clementino Fraga Filho, Federal University of Rio de Janeiro, Rio de Janeiro, Brazil; 4 Chair of Epidemiology, TUM School of Medicine and Health, Technical University of Munich, Munich, Germany; 5 Institute of Health and Wellbeing, Federation University, Churchill, Australia; 6 School of Health Sciences, University of Newcastle Australia, Callaghan, Australia; 7 Department for Evidence-based Medicine and Evaluation, University for Continuing Education Krems, Krems, Austria; 8 Núcleo de Estudos e Pesquisas em Atenção ao Uso de Drogas (NEPAD), Rio de Janeiro State University (UERJ), Rio de Janeiro, Brazil; 9 Sports Medicine Unit, National Hospital of Kandy, Kandy, Sri Lanka; 10 Faculty of Allied Health Sciences, University of Peradeniya, Peradeniya, Sri Lanka; 11 Geriatrics and Long-Term Care Department, Rumailah Hospital, Hamad Medical Corporation, Doha, Qatar; 12 Centre for Evidence-Based Medicine Odense (CEBMO) and Cochrane Denmark, Department of Clinical Research, University of Southern Denmark, Odense, Denmark; 13 Faculty of Spatial Management and Landscape Architecture, Department of Systems Research, Wroclaw University of Environmental and Life Sciences, Wroclaw, Poland; 14 Research Group of Urban Ageing, Faculty of Social Work and Education, The Hague University of Applied Sciences, Den Haag, The Netherlands; 15 School of Health, Wellbeing and Social Care, The Open University, Milton Keynes, United Kingdom; 16 Department of Nutrition, Nursing School, Universidade Federal de Minas Gerais, Minas Gerais, Brazil; 17 Directorate-General for Health and Food Safety, European Commission, Brussels, Belgium

**Keywords:** people living with dementia, digital health technologies, evidence-based medicine, telehealth, telemedicine, PRISMA

## Abstract

**Background:**

Population aging has intensified the global burden of dementia, creating significant challenges for patients, caregivers, and health care systems. While traditional in-person dementia care faces barriers, digital health technologies offer promising solutions to enhance accessibility, efficiency, and patient-centered care. However, evidence on applicability, safety, and effectiveness in dementia care remains fragmented, underscoring systematic evaluation.

**Objective:**

This study aims to assess the effectiveness, applicability, safety, and cost-efficiency of telemedicine technologies in dementia care, providing a comprehensive summary of evidence spanning clinical, psychological, socioeconomic, and operational impacts for persons living with dementia and their caregivers and assess alignment with the World Health Organization (WHO) Age-friendly Cities and Communities’ Framework and Dementia Inclusive Society Framework.

**Methods:**

An overview of systematic and scoping reviews was conducted following a search in 5 databases (MEDLINE, Embase, Scopus, Epistemonikos, and Cochrane Database of Systematic Reviews), with a gray literature search on February 20, 2024. Eligible studies reported predefined outcomes related to telemedicine interventions for integrated dementia care, including effects on mental health, quality of life, physical activity, hospitalization, financial costs, safety, social isolation, and motor function. Screening and data extraction were performed by 10 reviewers. The findings were synthesized using the Thematic Analysis in Meta-Evidence (TAME) methodology, combining thematic and lexical analyses with single-proportion meta-analysis for comprehensive qualitative-quantitative synthesis. The methodological quality was assessed using the AMSTAR 2 (A Measurement Tool to Assess Systematic Reviews), with GRADE-CERQual (Confidence in the Evidence from Reviews of Qualitative Research) for outcomes’ confidence in evidence.

**Results:**

Ninety-one reviews provided evidence on the impact of telemedicine in dementia care. The most frequently reported outcomes were the effects of remote interventions on psychiatric and psychological well-being, particularly depression and anxiety (relative frequency of occurrence [RFO]=65%, 95% CI 54-75, moderate certainty of evidence). Fifty-seven studies highlighted the positive impact of telemedicine and telehealth on satisfaction and quality of life for persons living with dementia, caregivers, and health care providers (RFO=63%, 95% CI 52-73, moderate certainty of evidence). Remote technology-related interventions for reducing falls and managing behavioral symptoms were also frequently reported (RFO=33% 95% CI 23-44], moderate certainty of evidence). These interventions showed effectiveness in alleviating social isolation and loneliness (RFO=31%, 95% CI 22-41, moderate certainty of evidence). The methodological quality of the included reviews varied significantly, with the majority rated as low or critically low quality.

**Conclusions:**

Telemedicine and telehealth have been demonstrated to be effective and valuable tools in dementia care, offering significant benefits across psychological well-being, quality of life, and social impacts for persons living with dementia and their caregivers. This supports the adoption and implementation of telemedicine in dementia care, aligning with the strategies outlined in the United Nations Decade of Healthy Ageing (2021‐2030).

## Introduction

The population aging has accelerated at an unprecedented pace in recent times [1]. Besides the economic, social, and political implications, aging also brings attention to the significant health challenges posed by progressive neurodegenerative disorders, commonly referred to as dementia. These disorders present substantial burdens for health and care providers, patients, families, caregivers, and policy makers [2,3].

Traditionally, dementia care has been delivered primarily through in-person consultations. However, providing such care has posed significant challenges, particularly in rural communities where access to specialized services is limited, and for an already overstretched and overwhelmed health and care workforce. To attend these appointments, patients and their caregivers often face numerous barriers, including logistical difficulties, time constraints, and financial burdens [4].

The advent of digital health technologies (DHTs) has revolutionized the landscape of dementia care. These innovations range from medication reminder systems to sophisticated machine learning algorithms (ie, artificial intelligence) designed to predict and prevent falls and other adverse events, offering new opportunities to enhance the quality and accessibility of care [5].

Our research group was among the first to analyze the applicability, safety, enablers, and facilitators of multiple DHTs in clinical practice [6-10]. Through a range of observational studies, randomized clinical trials, and evidence-based medicine research, we have demonstrated the significant impact of DHTs across multiple modalities, including in medical and health care practice [7,11], cardiology and cardiovascular care [12,13], diabetology and endocrinology [12], dermatology [14], gynecology, and obstetrics [15]. Building on this foundation, we undertook an extensive bibliometric analysis to synthesize existing evidence on DHTs specifically tailored for holistic and integral dementia care [16]. This analysis highlighted critical methodological limitations and revealed a significant knowledge gap in the collation and systematization of global findings related to the use of telemedicine and telehealth for dementia care. The identified knowledge gap underscores the need for further research initiatives to enhance the integration of these technologies into dementia care practices. It highlights the need for horizontal, collaborative, and transformative partnerships, avoiding duplication of efforts.

Expanding on this work, the remote delivery of health care services offers a promising approach for addressing needs across various medical specialties, including Neurology and Psychiatry [17-19]. Recognizing gaps identified in our initial bibliometric analysis, we present this second publication in a series of studies evaluating the impact of digital health technologies on dementia care, focusing on their effects on patients and caregivers [20].

This study critically examines existing systematic and scoping reviews to assess the applicability, safety, and effectiveness of telemedicine and telehealth services in improving multiple aspects of dementia care. These aspects include clinical outcomes (eg, cognitive function, behavioral symptoms, and quality of life), mental health (eg, the psychological and psychiatric well-being of patients and caregivers), process-related outcomes (eg, medication adherence, patient engagement, and health care use), and economic factors (eg, cost-effectiveness analyses). To note, our study resonates with large-scale health projects launched within the EU4Health program, that is, a direct grant to Member States Joint Action addressing Dementia and Health (JADE) aimed at prevention and early detection leveraging cutting-edge technology, which, combined, highlights the importance of improving dementia care frameworks and builds upon unprecedented person-centered assistance models [21]. In particular, on the one hand, clinical care pathways for patients living with dementia are designed to establish and foster high-quality evidence-based best practices (tailored upon 8 pilot initiatives within 5 European Union countries); while on the other hand, person-centered new care models emphasize recognizing unmet needs in determined practices through the revision and analysis of at least 10 care frameworks for dementia and other neurocognitive abnormalities [21] in 6 European Union countries. In summary, these initiatives positively impact the development of applicable and valuable insights into the definition of telemedicine and telehealth-mediated interventions involved in dementia care, highlighting the need for scalable, data-based solutions to improve patient-centered assistance.

The findings of this study aim to provide valuable insights into the real-world effectiveness, applicability, safety, and cost-efficiency of telemedicine and telehealth technologies in dementia care. This study serves as a practical guide for practitioners, health authorities involved in decision-making, and patients and their caregivers, offering evidence-based perspectives on the role of telecommunication and remote health care services in dementia management.

## Methods

### Overview

This overview of systematic reviews is based on a large project registered with the International Prospective Register of Systematic Reviews (CRD42024511241) [22]. Given the study design, which is based on secondary data, no ethical review was required. The study adheres to the PRISMA 2020 (Preferred Reporting Items for Systematic Reviews and Meta-Analyses) reporting guidelines [23]. All methods used in the research were pre-established before data collection, minimizing potential biases commonly associated with other evidence synthesis approaches.

### Search Strategy and Selection Criteria

First, an experienced information librarian (LØ) systematically searched multiple databases for article retrieval. The search strategy was tailored in collaboration with specialists in epidemiology, evidence-based medicine, geriatrics and gerontology, neurology, psychiatry, psychology, and policy makers (Multimedia Appendix 1) [5,24-113]. In our overview, 5 leading databases were searched for eligible reviews (MEDLINE, Embase, Scopus, Epistemonikos, and Cochrane Database of Systematic Reviews). Furthermore, the first 300 hits on Google Scholar were screened, potentially identifying studies from the gray literature. The systematic search was performed on February 20, 2024. The inclusion criteria were primarily related to systematic or scoping reviews reporting relevant data on the applicability, effectiveness, and safety of telemedicine and telehealth services for the management of people living with dementia. Included reviews were deemed eligible with reported relevant data on people living with dementia (in any form of neurodegenerative disorders), including Alzheimer disease, vascular dementia, Lewy body dementia, and frontotemporal dementia, regardless of the international classification used (eg, *ICD-11* [*International Classification of Diseases, 11th Revision*], *ICD-10* [*International Statistical Classification of Diseases and Related Health Problems, Tenth Revision*], or *DSM-5* [*Diagnostic and Statistical Manual of Mental Disorders* {Fifth Edition}]). Although the diagnosis of dementia commonly occurs among the older population, we are aware of early presentation of the disease among younger individuals. Therefore, we considered eligible for inclusion primary records including young and older people living with dementia. We included reviews that reported relevant data on patient outcomes and evaluated the impact of these telemedicine and telehealth on the daily lives of formal or informal caregivers. We included systematic and scoping reviews from multiple countries, regardless of the publication language. Definitions for each type of review are available in previously published sources [114,115]. Narrative reviews were excluded from eligibility due to their higher susceptibility to bias [116].

After obtaining the records from the databases, duplicates were removed on EndNote (Clarivate Analytics), and the final list of studies was imported into Covidence (Veritas Health Innovation). Ten investigators on our team performed the independent and dual-stage screening process (IJBN, HMA, IW, CC, GD, AS, IK, VGB, BS, and AOM). Similarly, for papers deemed eligible for inclusion, the same authors performed data extraction independently and in duplicate. Any discrepancies were resolved through discussion facilitated via an online smartphone-based instant messaging platform. A complete list of included and excluded studies with reasons for exclusion after full-text review has been developed in Multimedia Appendix 1 [5,24-113].

### Definition of Terminologies

We understand that telemedicine and telehealth are commonly used interchangeably. However, for practical differentiation, we used the World Health Organization’s (WHO’s) definitions [117,118] of telemedicine described as “the delivery of health-care services where distance is a critical factor, by all health-care professionals using information and communication technologies for the exchange of valid information for diagnosis, treatment and prevention of disease and injuries all in the interests of advancing the health of individuals and their communities—page 2” [117]. Notably, we emphasize that telemedicine is a component of telehealth, primarily applied to educational purposes, along with different applications wherein information and communication technologies are used to enhance health care services [117,119,120].

We used the previously published definition for systematic reviews, defined as “a research report which searched for primary research studies on a specific topic using an explicit search strategy, had a detailed description of the methods with explicit inclusion criteria provided, and provided a summary of the included studies either in narrative or quantitative format (such as a meta-analysis)”—page 3 [121]. We included Cochrane and non-Cochrane systematic reviews, with or without meta-analysis, that reported on the clinical, mental, managerial, or economic effects of telemedicine and telehealth services in dementia care. Scoping reviews were defined as evidence synthesis studies that “systematically identify and map the breadth of evidence available on a particular topic, field, concept, or issue, often irrespective of source (ie, primary research, reviews, and nonempirical evidence) within or across contexts”—page 1 [122]. Studies reporting relevant data on different modalities of DHTs were included. However, when multiple types of digital intervention were described, only results specific to telemedicine and telehealth were extracted and accounted for in the final analysis.

### Data Extraction and Management

Ten researchers (IJBN, HMA, IW, CC, GD, AS, IK, VGB, BS, and AOM) independently participated in data extraction using a prevalidated extraction Microsoft Excel (Microsoft Corporation) spreadsheet. Each study underwent data extraction by 2 reviewers and was re-evaluated by a third reviewer to verify the accuracy of selected data. We extracted key characteristics of each review, including the title and first author, year of publication, journal, review objective, main population studied, number of included primary studies, key reported outcomes relevant to this analysis, and the review category (systematic or scoping). Regarding review findings, we qualitatively analyzed telemedicine and telehealth’s reported impact and role in the following areas: (1) psychiatric or psychological outcomes; (2) satisfaction and quality of life for patients, families, or health care providers; (3) metrics of habitual physical activity; (4) hospitalization rates; (5) financial aspects; (6) safety outcomes; (7) clinical improvement of underlying diseases beyond neurological or dementia-related disorders; (8) social isolation and loneliness; (9) motor progression in clinical and research contexts; and (10) prevention of falls and management of behavioral and psychological symptoms. The explanation of how findings were collated and the processes is briefly explained under the “Data Analysis and Synthesis” section.

In addition, we assessed the reviews to identify the influence of telemedicine and telehealth on at least one of the 10 domains of WHO Age-friendly Cities and Communities’ Framework [123] adapted to Dementia Inclusive Society [124], including housing, social participation, respect and social inclusion, civic participation and employment, communication and information, community support and health services, outdoor spaces and buildings, transportation, safety, and caregiver support, alongside medical management [123]. This mapping was conducted by 2 independent researchers and validated by an expert on age-friendly environments at the WHO Regional Office for Europe. The WHO Age-Friendly Cities and Communities’ Framework is a key component of the United Nations Decade of Healthy Ageing (2021‐2030) program, aligning with the Sustainable Development Goals [125] and encompassing a series of actionable activities. Finally, we extracted all necessary information to evaluate the overall quality of the included systematic reviews.

### Assessment of the Methodological Quality of Included Reviews and Certainty of the Evidence in Included Reviews

We independently evaluated the methodological quality of the included systematic reviews based on the AMSTAR 2 (A Measurement Tool to Assess Systematic Reviews) measurement approach [126]. The tool consists of 16 domains, 7 of which are prioritized due to their critical impact on a review’s validity, transparency, and replicability [126]. While we chose not to report a final summary score, we accounted for the implications and effects of inadequate ratings for each evaluated domain in our final analysis [126]. Systematic reviews were classified as having “high,” “moderate,” “low,” or “critically low” quality. The certainty of evidence for the main findings of the reviews was assessed using the GRADE-CERQual (“Confidence in the Evidence from Reviews of Qualitative Research”) methodology [127]. This evaluation considered 5 key factors influencing the evidence: methodological limitations, coherence, data adequacy, relevance, and publication bias. The confidence in each qualitative primary review finding was rated as “high,” “moderate,” “low,” or “very low.”

### Data Analysis and Synthesis

Given the included studies’ methodological heterogeneity, we used qualitative and quantitative approaches to summarize the findings, adhering to established methodological guidance for conducting mixed methods systematic reviews [128]. Our research group recently published the Thematic Analysis in Meta-Evidence (TAME) Visualization Methodology, specifically designed for reporting results from studies predominantly based on qualitative evidence, using thematic and lexical analysis principles. The TAME methodology combines content analysis with single-proportion meta-analysis, enabling the calculation of the overall proportion of studies reporting a particular finding. This integrated approach enhances the interpretation and visualization of mixed methods evidence, providing a comprehensive understanding of the data. Final estimates were expressed as the relative frequency of occurrence (RFO) and 95% CI. Our study used single-proportion meta-analysis to graphically and numerically present the results. This method facilitates the conversion of qualitative data into quantifiable outcomes, enabling a clear and straightforward evaluation of qualitative findings through mathematical representation. R (version 4.4.0; R Core Team) was the software used for statistical analysis.

### Ethical Considerations

This study was exempt from ethical approval as it did not involve original human data collection.

## Results

### Search Results and Characteristics of Included Studies

**Figure 1.**
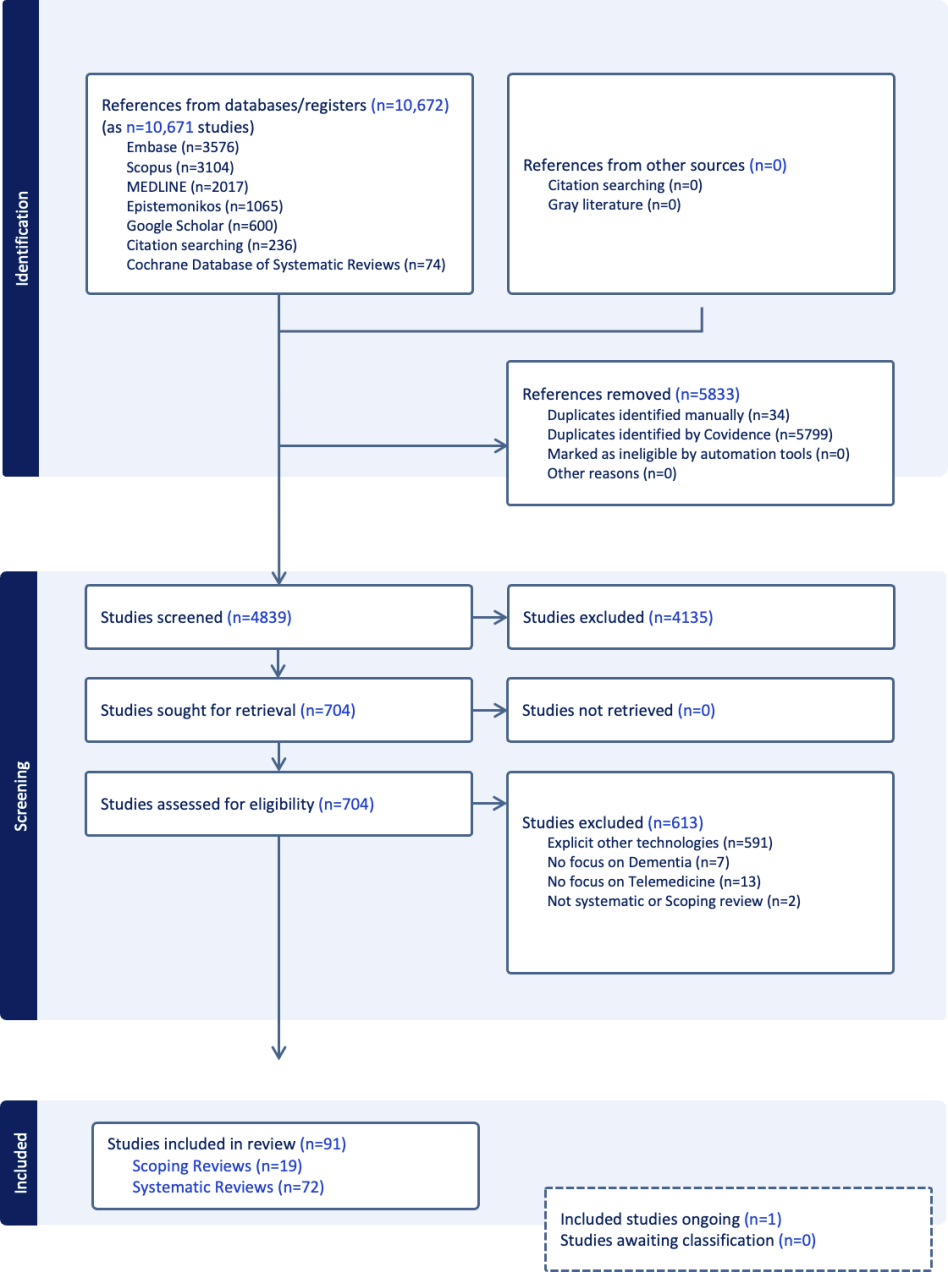
Covidence analysis.

In our bibliometric analysis, we shortlisted 704 studies within the broader category of DHTs applicable to dementia care [20]. After prioritizing systematic and scoping reviews focusing on specific modalities of telemedicine-related digital intervention, 91 reviews [5,24-113] were included in our final analysis (Figure 1). The ipsilateral main objectives and participants of the included reviews, published between 2008 and 2024, are summarized in Multimedia Appendix 2 [5,24-113]. The number of publications remained relatively stable from 2008 to 2015, with one to 3 reviews published annually. However, a notable growth phase began in 2016, accelerating further during the COVID-19 pandemic, significantly increasing research activity in this field. This surge was evidenced in the publications of 13 reviews in 2021 [26,44,47,60,82,84,87,88,94,95,99-101] and 18 in 2023 [35,37,39,48,50,53,62,66,75,76,92,93,97,98,102,104,109,111]. Most reviews were published in the *Aging & Mental Health* (n*=*6), *International Journal of Geriatric Psychiatry* (n*=*5), and *Cochrane Database of Systematic Reviews* and *Journal of Medical Internet Research* (n*=*4). Several of the included reviews highlighted findings related to remote cognitive assessments and populations in remote or resource-limited settings, reflecting the importance of accessibility in dementia care. Among the 91 included reviews [5,24-113], the majority were systematic reviews (n*=*72), while 19 [33,35,37,39,43,48,59,63,66,68,70,81,84,91,93,98,109-111] were classified as scoping reviews. Although we did not assess the percentage of overlapping primary studies across the included reviews, the total number of primary studies encompassed within these reviews amounted to 2929 records, reflecting a substantial body of evidence analyzed in the present overview of reviews.

The included reviews predominantly evaluated telemedicine and telehealth technologies’ effectiveness, feasibility, and acceptability in dementia care. Many included reviews (28/91, 30%) also assessed remote cognitive assessments, primarily using telephone- and video-based tools, focusing on mild cognitive impairment and dementia. Likewise, several studies (49/91, 53%) explored the impact of psychoeducational, psychotherapeutic, and social support online interventions to support caregivers’ mental health and well-being. Also, some reviews highlighted the potential of telemedicine and telehealth services to enhance clinical outcomes, ensure caregiver support, facilitate safety outcomes, and decrease loneliness for people living with dementia.

**Figure 2.**
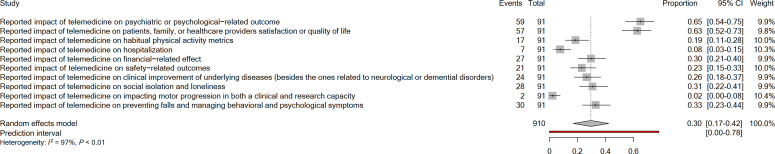
Outcome-specific relative frequency of occurrence estimates.

Outcome-specific RFO estimates ranged from 2% (95% CI 0‐8, related to the impact of telemedicine on motor progression in both clinical and research contexts) to 65% (95% CI 54‐75, related to the reported impact of telemedicine on psychiatric or psychological outcome), as shown in Figure 2. Below, we summarize findings for the top 5 most prevalent domains, with additional findings for 5 other domains provided in Table 1.

**Table 1.** Summary of review findings and confidence in the evidence for the use of telemedicine and telehealth services for multifaceted dementia care.

GRADE-CERQual components	RFO^a^ % [95% CI]	Methodological limitations^b^	Coherence	Adequacy of data	Relevance^c^	Overall assessment^d^
Reported impact of telemedicine on psychiatric or psychological-related outcome	65% [54‐75]	Moderate concerns	Moderate concerns^e^	Moderate concerns^f^	No or very minor concerns^c^	Moderate confidence
Reported impact of telemedicine on patients, family, or health care providers' satisfaction or quality of life	63% [52‐73]	Moderate concerns	No or very minor concerns^g^	Moderate concerns^h^	No or very minor concerns^c^	Moderate confidence
Reported impact of telemedicine on habitual physical activity metrics	19% [11‐28]	Moderate concerns	Moderate concerns^i^	Moderate concerns^j^	No or very minor concerns^c^	Moderate confidence
Reported impact of telemedicine on hospitalization	8% [3‐15]	Moderate concerns	Serious concerns^k^	Serious concerns^l^	Moderate concerns^m^	Very low confidence
Reported impact of telemedicine on financial-related effect	30% [21‐40]	Moderate concerns	Moderate concerns^n^	Moderate concerns^j^	No or very minor concerns^c^	Moderate confidence
Reported impact of telemedicine on safety-related outcomes	23% [15‐33]	Moderate concerns	No or very minor concerns^g^	Moderate concerns^j^	No or very minor concerns^c^	Moderate confidence
Reported impact of telemedicine on clinical improvement of underlying diseases (besides the ones related to neurological or dementia disorders)	26% [18‐37]	Moderate concerns	Moderate concerns^o^	Moderate concerns^j^	No or very minor concerns^p^	Moderate confidence
Reported impact of telemedicine on social isolation and loneliness	31·0% [22·0‐41·0]	Moderate concerns	Moderate concerns^i^	Moderate concerns^j^	No or very minor concerns^c^	Moderate confidence
Reported impact of telemedicine on impacting motor progression in both a clinical and research capacity	2% [0‐8]	Moderate concerns	Serious concerns^k^	Serious concerns^l^	No or very minor concerns^c^	Very low confidence
Reported impact of telemedicine on preventing falls and managing behavioral and psychological symptoms	33% [23‐44]	Moderate concerns	Moderate concerns^i^	No or very minor concerns^h^	No or very minor concerns^c^	Moderate confidence

a RFO: relative frequency of occurrence.

b We downgraded one level of confidence in the evidence based on the methodological quality of included systematic reviews and not based on the methodological limitations of primarily included studies. The rationale is that the AMSTAR 2 tool has 7 strict critical domains, which, if they occur at least once, decrease overall confidence by 2 levels. Nevertheless, since several experts have already suggested that the reporting of many items in the PRISMA (Preferred Reporting Items for Systematic Reviews and Meta-Analyses) statement is suboptimal, we believe that this lack of reporting or evaluation might be associated with a “mass effect,” where researchers simply follow an inadequate pattern. Therefore, we decreased one level in the certainty of evidence instead of 2 levels on reviews’ methodological limitations.

c Based on the alignment of most studies reporting this outcome in the reality of telemedicine and telehealth interventions for dementia care, we considered this domain to have minor concerns evidencing not significant reduction of confidence in the relevance of collated results and with a well-coverage of needed analyses.

d Although most of our included reviews were classified as “very low methodological quality” using the AMSTAR 2 tool, we believe that the reported data are significant enough not to decrease the confidence level primarily based on the methodological quality.

e We downgraded one level for coherence because of inconsistent findings across included reviews, ambiguous or differential measurement of outcomes, and due to competing theories or formats reported (within the modality of telemedicine and telehealth, we observed competing theoretical explanations on different degrees of effectiveness according to the intervention type).

f The evaluated data are overall rich in details for caregivers’ findings and emotional well-being. However, inconsistent data volume decreased the confidence in some reported findings, specifically, those associated with psychiatric outcomes.

g Collated results on this outcome demonstrated elevated systematic coherence, with consistent evidence of convenience, reduction of travel burden, and remarkable positive feedback from patients and their caregivers. Likewise, caregiver self-efficacy and emotional well-being outcomes demonstrated generally elevated coherence. With regard to safety outcomes, we judged this outcome as having minor concerns because safety outcomes and benefits are consistently reported within studies (particularly related to fall prevention, monitoring, and reducing physical harm).

h Synthesized evidence related to satisfaction with telemedicine interventions for dementia care is adequate and sufficiently robust across included reviews, though the impact on quality of life is mostly heterogeneous. Related to the impact of telemedicine on preventing falls and managing behavioral and psychological symptoms, the obtained data were deemed adequate, with a variety of interventions covering both falls and behavioral symptoms.

i Included reviews contained relevant evidence related to the enhancement of physical activity, adherence to exercise programs following the intervention for patients living with dementia. However, findings are not consistent across all studies. As far as the potential of telemedicine to reduce social isolation and loneliness, we judged it as moderate evidence because, overall, qualitative evidence collated is consistent, but quantitative outcomes are mixed, with some showing no improvement. Related to the impact of telemedicine in preventing falls and managing behavioral and psychological symptoms of dementia, multiple positive findings were observed across included reviews, though with some inconsistent results across studies.

j Judged as moderate concerns due to lack of detailed report or uneven reporting across themes approached within included studies.

k Findings for hospitalization outcomes are overall scattered and inconsistent, with some minor isolated positive reports.

l We downgraded this domain due to limited detailed data within included reviews; besides, whenever available, there was no significant impact on hospitalization. Regarding the impact of telemedicine on motor progression in either clinical or research settings, we downgraded the evidence because of limited evidence, with most studies reporting no data.

m We observed the reporting of some relevant findings across included reviews; however, they were primarily focused on emergency department metrics and readmission rates, which hinder and limit direct relevance.

n We observed that the obtained findings related to the financial impact of telemedicine are generally consistent, but with some variability in reporting of long-term savings and cost parameters.

o We systematically observed that merged results generally align but with relevant inconsistencies in clinical outcomes and variability in effectiveness across the different interventions delivered.

p As we considered cognitive clinical outcomes in a separate domain, we are judging this domain as nonserious concerns because included reviews systematically reported relevant improvements in chronic disease management.

### Impact of Telemedicine and Telehealth on Psychiatric and Psychological Outcomes

Globally, the report of the impact of telemedicine and telehealth on both psychiatric and psychological outcomes was the most frequent among the 10 included dementia-inclusive domains (RFO=65%, 95% CI 54‐75; weight: 9.9%). Included reviews predominantly demonstrated the positive impact of these technologies in dementia care, either by the reduction of depression and anxiety symptoms among included patients (particularly as a result of telephone- or video-based cognitive behavioral therapy and psychoeducation), enhancement of cognitive and mental health outcomes (expressed by improved cognitive function [evidenced by increased Montreal Cognitive Assessment and Quality of Life–Alzheimer Disease {QOL-AD} scores]), and decrease of informal or formal caregiver burden and stress attributed to the delivery of dementia care. Similarly, several reviews highlighted the enhanced social support and self-efficacy associated with the use of telemedicine and telehealth, which ultimately provided emotional and social benefits while helping caregivers manage stress more effectively. However, despite these positive outcomes, some reviews reported mixed or limited effectiveness in addressing psychiatric and psychological outcomes. Notably, studies that found no significant difference between telemedicine or telehealth interventions and standard care often concluded that the interventions were not inferior to traditional approaches. For instance, one meta-analysis observed inconsistent benefits regarding caregiver workload and depression. It reported no significant effect on caregiver burden (standardized mean difference=0.05; 95% CI –0.20 to 0.30) across multiple studies, highlighting variability in effectiveness.

In addition, some reviews identified challenges associated with telemedicine and telehealth services. Fatigue and frustration were commonly reported as adverse effects of online assessments, particularly among users (patients or caregivers) unfamiliar with the technologies used. These findings underscore the need to address usability and accessibility to optimize the impact of telemedicine on dementia care.

### Impact of Telemedicine and Telehealth on Satisfaction and Quality of Life for Patients, Families, or Health Care Providers

Fifty-seven studies (RFO=63%, 95% CI 52‐73; weight=9.8%) reported on the impact of telemedicine and telehealth on satisfaction or quality of life among patients, families, and both formal and informal health care providers. Overall, the majority of studies indicated a positive impact, particularly associated with interventions such as videoconferencing, telephone-based support, and telepsychiatry services, which were linked to high satisfaction rates among end users.

In studies that explicitly measured satisfaction, reported rates ranged from 80% to 90%, highlighting the broad acceptance of these remote care approaches [101,108]. Telemedicine and telehealth were frequently described as convenient and accessible, with benefits such as reduced travel-related burden and enhanced user support. Furthermore, most studies observed improved quality of life for both patients and caregivers, often through increased self-efficacy in managing work- or task-related responsibilities and by alleviating stress associated with traditional in-person care delivery.

In addition, we observed that some technologies (ie, online cognitive rehabilitation systems and remote activity monitoring platforms) facilitated patients’ empowerment (by promoting their independence associated with care) and improved their engagement in care. Online assistance groups and peer-support frameworks were also reported to encourage and facilitate social interaction among users, reduce caregiver distress, and enhance users’ emotional well-being. Regarding the negative and mixed impacts of telemedicine and telehealth services on users’ satisfaction and quality of life, only a few studies (9/91, 9.8*%*) reported nonsignificant improvement on these outcomes. For instance, one systematic review and meta-analysis of internet-based interventions revealed mixed findings, with some studies showing minimal gains in quality of life and others reporting no significant differences compared to standard care [72]. Some studies also reported adverse effects derived from the use of telemedicine and telehealth, particularly related to user satisfaction, such as user-related anxiety reported by caregivers, discomfort during the use of video consultations, and lack of physical contact [25,37]. Likewise, some reviews suggested the limited impact of telemedicine and telehealth in reducing caregiver work-related burden [35]. This conclusion was supported by findings from multiple meta-analyses, which reported no significant improvements in caregiver burden.

### Impact of Telemedicine and Telehealth on Fall Prevention and Management of Behavioral and Psychological Symptoms

We extracted relevant data from reviews (30/91, 33%) to evaluate the impact of telemedicine and telehealth on preventing falls and managing behavioral and psychological symptoms, ranking it as the third most prevalent domain (RFO=33%, 95% CI 23%‐44%; weight=9.9%). The pooled estimates were supported by multiple reviews highlighting the effectiveness of remote assistive technologies and teleassistance platforms in reducing falls with metrics indicating a general fall reduction of 28% to 31% and a decrease in indoor falls ranging from 32.7% to 63.8%. Furthermore, we observed that telemedicine technologies demonstrated notable effectiveness in managing behavioral and psychological symptoms. Reviews reported improvements in overall well-being, reductions in disruptive behaviors, and effectiveness in addressing agitation, hallucinations, and sleep disturbances, further emphasizing the potential of telemedicine to enhance dementia care in these areas. We observed that some studies demonstrated that remote interventions promoted a better caregiver response and a decrease in negative-derived reaction to improved caregiver responses and reductions in negative caregiver reactions to deviant behaviors. Variability in the reported magnitude of effect for this outcome was relatively limited, with only a small number of reviews indicating no significant improvements in the occurrence of disruptive behaviors or reductions in falls among people living with dementia who received remote interventions.

### Impact of Telemedicine and Telehealth on Social Isolation and Loneliness

Remote support technologies, particularly video conferencing systems and online peer assistive platforms, emerged as a promising alternative for reducing social isolation and loneliness among people living with dementia. These technologies ranked as the fourth most frequently reported outcome (RFO=31%, 95% CI 22%‐41%; weight=9.9%). The identified technologies facilitated sustained social engagement by fostering meaningful interactions between patients and their caregivers. By enabling patients to engage in community activities, participate in peer support networks, and share their lived experiences in safe, supportive environments, these technologies played a critical role in strengthening social connectedness. This, in turn, contributed to improved emotional well-being and reduced feelings of isolation among individuals living with dementia and their caregivers. For instance, one review found that approximately 86% of enrolled individuals in virtual cognitive stimulation therapy reported improved engagement in social tasks, with 67% of caregivers observing a notable reduction in loneliness through peer interactions. The qualitative evidence consistently highlighted the emotional benefits of telemedicine and telehealth technologies in dementia care. Users frequently reported reduced feelings of loneliness, improved relationship quality, and a strong sense of solidarity fostered by online communities. While these emotional advantages were evident across included reviews, the quantitative outcomes remained less definitive. For instance, one review found that video conference-based interventions improved metrics related to perceived social support but did not demonstrate statistically significant changes in support domain scores. This disparity underscores the need for further research to quantitatively validate the emotional benefits reported by users.

### Overall Findings Aligned With the WHO’s Dementia Inclusive Society Framework

**Figure 3.**
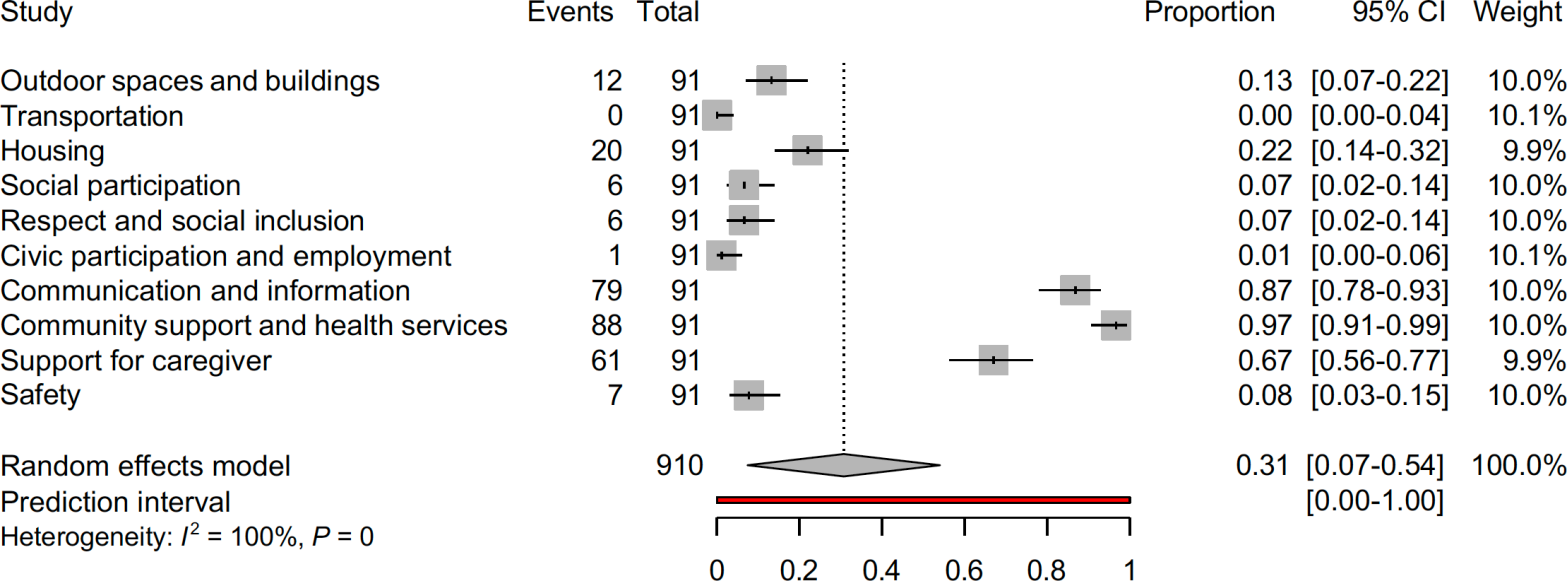
Alignment of findings from the included reviews with the World Health Organization Age-friendly Cities and Communities’ Framework.

**Figure 4.**
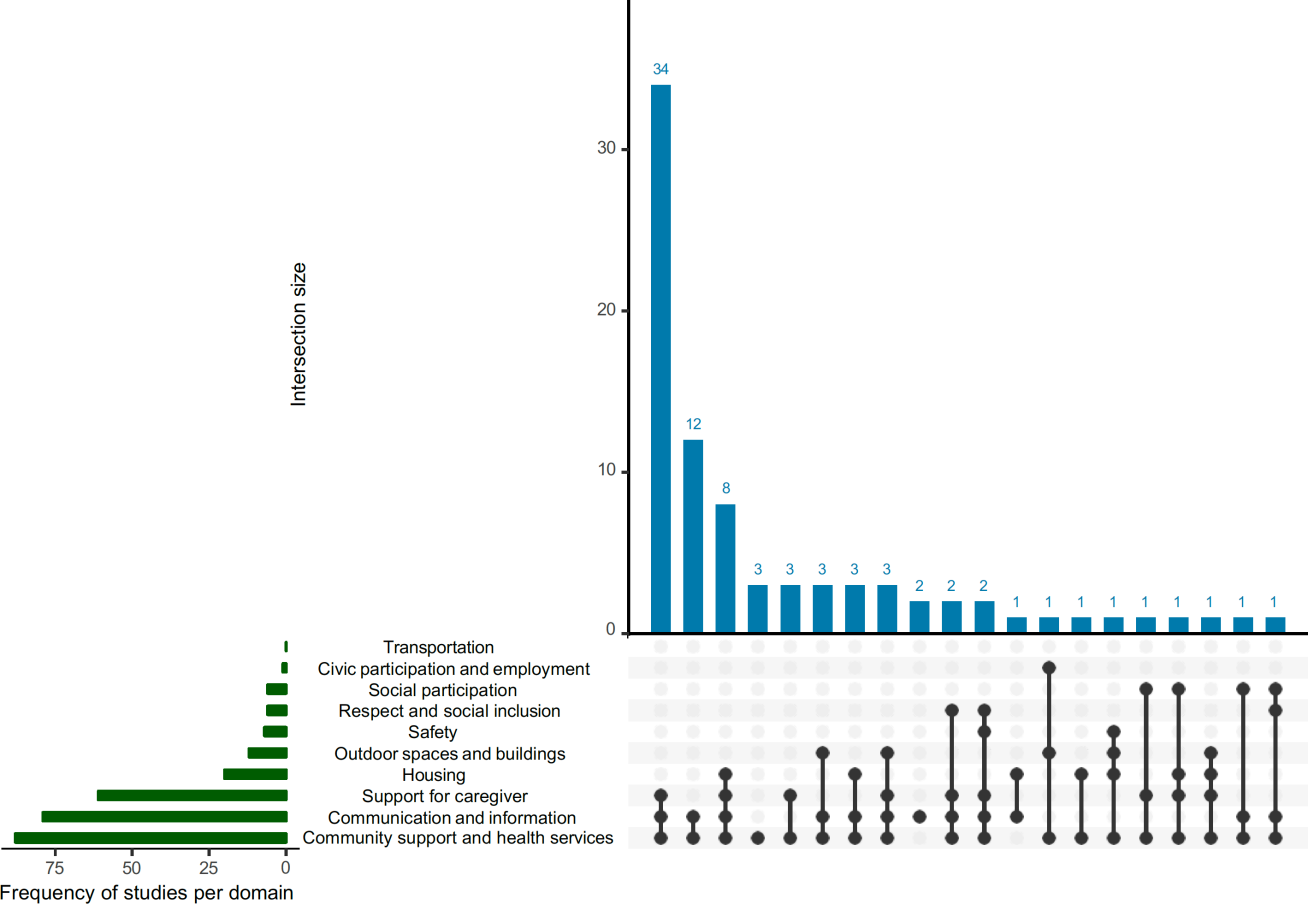
Domain co-occurrence in the Dementia Inclusive Society Framework for telemedicine interventions. The left bar chart (green) shows the frequency of studies addressing each domain, while the upper bar chart (blue) depicts the number of studies covering specific combinations of domains (intersection size). Black dots in the matrix indicate the domains included in each combination, with connected dots representing multiple-domain overlaps.

Our meta-analysis, presented in Figure 3, illustrates the alignment of findings from the included reviews with the WHO Age-friendly Cities and Communities’ Framework and its derivative Dementia Inclusive Society Framework. The analyses reveal that the domains with the highest pooled estimates are “community support and health services,” “communication and information,” and “caregiver support” (RFO=97%, 95% CI 91%‐99%, 87%, 95% CI 78%‐93%, and 87%, 95% CI 78%‐93%, respectively). In contrast, domains, such as “transportation” and “civic participation and employment,” were the least represented, with RFO of 0% (95% CI 0%‐4%) and 1% (95% CI 0%‐6%), respectively. Figure 4, an upset plot illustrating domain co-occurrence, highlights key patterns in how telemedicine interventions are applied in dementia care as well as in the daily routine of people living with dementia. The analysis emphasizes a predominant focus on “Community support and health services” and “Communication and information,” which often intersect. This intersection underscores telemedicine’s critical role in connecting patients and caregivers to essential health services and informational resources, effectively addressing fundamental care needs remotely. Less frequently observed domains, such as “Social participation” and “Respect and social inclusion,” also intersect with other domains, pointing to the untapped potential of telemedicine to foster social connectedness and reduce isolation. These findings suggest that while telemedicine is already proving essential in many areas, its capacity to promote broader social integration remains underexplored and warrants further investigation. Likewise, people living in rural communities can benefit from using telemedicine, connecting them with medical professionals to maintain a level of connectivity [129,130].

### Methodological Quality of Included Systematic Reviews

The AMSTAR 2 evaluation of included systematic reviews is summarized in Multimedia Appendix 3 [5,24-113]. All 4 Cochrane reviews were rated as high quality [46,47,58,88]. However, most non-Cochrane reviews were classified as “critically low quality” due to several major and minor methodological shortcomings. These included the absence of published protocol or failure to report protocol deviations, incomplete or noncomprehensive search strategies, omission of the final list of excluded studies, inadequate assessment of the risk of bias in individual studies and their implications, improper use of meta-analytical approaches, and lack of publication bias analysis. These issues highlight significant methodological gaps in many of the included non-Cochrane reviews.

### Certainty of Evidence and Qualitative Synthesis

Most of the included reviews used either qualitative methods alone or a mixed methods approach for evidence synthesis, integrating qualitative and quantitative data using our thematic, content, and lexical-based methodology. Many reviews provided detailed analyses of the overall impact of telemedicine and telehealth services on both individuals living with dementia and their caregivers (formal or nonformal). Table 1 presents the summary of qualitative findings and associated confidence assessments.

Several themes emerged as systematically homogeneous across the included reviews, with the direction of effect clearly leaning toward either positive or negative outcomes. Quality appraisals were conducted exclusively for outcomes related to the reported impact of telemedicine across these areas. Overall, our evaluation demonstrated promising results for the 10 primary outcomes, although the findings were somewhat heterogeneous across standardized domains. This variability underscores the need for further research to refine and standardize the evidence base supporting the integration of telemedicine in dementia care.

## Discussion

### Principal Findings

Our review included 91 systematic and scoping reviews examining the applications of telemedicine and telehealth in dementia care [5,24-113]. While the findings were promising, evidence on the effectiveness of remote health interventions across clinical, psychological, and socioeconomic outcomes was often mixed or inconclusive. The findings suggested that telemedicine and telehealth interventions have the potential to improve psychiatric and psychological symptoms, alleviate caregiver burden and emotional stress for both formal and informal caregivers, and enhance the quality of life for patients and other end users. Evidence also pointed to the potential of telehealth to reduce social isolation, aid in fall prevention, and effectively address behavioral symptoms, although the consistency of these effects across reviews varied. Although less frequently addressed, some reviews indicated possible financial benefits associated with telemedicine and telehealth services in dementia care. However, significant methodological limitations in the included reviews, including variability in outcomes of interest and heterogeneity among primary studies, introduced uncertainty related to the restricted generalizability and robustness of our results. These limitations underscore the need for further high-quality research to support more definitive and reliable recommendations.

We observed that the use of telemedicine and telehealth interventions revealed significant psychiatric and psychological benefits for both patients and caregivers (moderate certainty of evidence). Our systematic synthesis demonstrated that these modalities of DHTs contribute to statistical and clinical reduction of depression, anxiety, and caregiver workload burden. In addition, some submodalities of remote services (ie, remote cognitive-behavioral therapy) and psychoeducational exercises have demonstrated an increase in mental health outcomes, with potential improvement in cognitive function and mood stability. Telemedicine and telehealth services benefit not only people diagnosed with dementia but also their caregivers and support networks. Our study highlights that many included reviews reported reduced stress levels and improved coping mechanisms, largely due to the convenience and accessibility of mental health–related resources provided through these technologies. Importantly, our findings do not categorically establish whether DHTs should be implemented as a supplement to traditional in-person care tools or as a replacement strategy.

Our findings align closely with the WHO’s Mental Health Action Plan 2013‐2030, which promotes enhancing access to mental health services based on high-quality scientific evidence and decreasing care inequities worldwide [131]. Recently, the WHO has emphasized the importance of tailored, population-specific health care strategies to address diverse needs in dementia care settings. While our findings indicated positive outcomes associated with telemedicine and telehealth interventions, caution is warranted before generalizing these results, as the evidence stems from heterogeneous settings. To advance the field, future studies should prioritize future customization in their design to address the unique needs of patients and caregivers more effectively.

In 2007, the WHO introduced the people-centered health care framework, advocating a critical shift from health care systems focused on diseases and institutions to systems designed around the needs of individuals—treating everyone as integral components of society [132]. For health care systems to be truly universal and accessible to all, including underserved, socially vulnerable, and marginalized populations, this renewed perspective must prioritize integration and person-centered care, ensuring that no one is left behind [130,133-136].

Our findings align with these global trends in medical research, demonstrating that telemedicine and telehealth interventions effectively deliver health care services to diverse groups, including patients, family members, and caregivers (moderate confidence level). These interventions also benefit individuals in resource-limited settings and isolated geographical areas [137-140]. Specifically, 57 reviews [24-27,31,32,35-38,41,42,44,45,47,48,52,53,56,58-60,62,64,66,68,69,71,72,78,80,82,83,86,88,90,91,93-111,113] (RFO=63%, 95% CI 52‐73) reported positive impacts on satisfaction and quality of life among patients, families, and health care providers. This personalized, microregional approach to care highlights telemedicine’s potential—particularly in dementia-related conditions—to enhance care convenience and actively engage patients and caregivers in health management, adhering to the principles of patient-centered care [141].

However, challenges remain in settings where the lack of physical presence in remote interventions slightly decreases user experience, particularly for certain conditions and among individuals unfamiliar with digital platforms [136,140]. In such scenarios, the feasibility, effectiveness, safety, and systematic implementation of telemedicine interventions require further evaluation. Given that an integrated, people-centered approach relies on core principles of equity in access, quality, responsiveness, participation, efficiency, and resilience, we emphasize the urgent need for adaptive telemedicine solutions. These approaches should not only address the diverse characteristics of users but also foster the cocreation of systems tailored to their unique life circumstances [136,142]. This includes developing hybrid care models that combine online and in-person care modalities, when feasible and appropriate, to optimize health care delivery and outcomes.

Social isolation and loneliness have been commonly classified as the century’s most damaging and prevalent human condition ever reported [143,144]. Accurately estimating the prevalence of loneliness and social isolation remains challenging due to limited and variable data. However, existing literature suggests that loneliness may affect up to 47.8% of adults aged 65 years and older [145]. Therefore, properly addressing these 2 events has potentially emerged as major areas where digital health (particularly telemedicine and telehealth solutions) contribute positively [139]. In our review, the impact of telemedicine and telehealth on social isolation and loneliness was reported in 28 reviews [25-27,31,32,36-38,41,45,48,49,53,65-67,91,93-95,98-100,104,106,109-111] (RFO=31%, 95% CI 22‐41; weight: 9.9% moderate certainty of evidence). Remote supporting technologies, such as videoconferencing systems and online peer assistive platforms, have proven to be promising tools in reducing social isolation and loneliness among people living with dementia. These technologies also offer structured opportunities for social engagement, effectively alleviating associated symptoms, particularly in older adults. Notably, our findings also support the United Nations Decade of Healthy Ageing (2021‐2030) by demonstrating that telemedicine interventions, such as virtual cognitive stimulation therapy, can significantly enhance social engagement and reduce feelings of loneliness. One study reported that 86% of participants experienced improved social connection and decreased sensations of loneliness. In addition, 67% of health caregivers noted a notable improvement in their social relationships with family members and the community following the adoption of telemedicine services. Despite these positive qualitative outcomes, slight inconsistencies in the quantitative synthesis for loneliness reduction and social participation among individuals living with dementia remain. These limitations highlight the need for further research to better quantify the effectiveness of telemedicine and telehealth in fostering social support and community engagement in dementia care. By addressing these gaps through robust and well-designed studies, the global understanding of telemedicine’s impact can be strengthened, supporting the WHO’s missions to create socially inclusive environments for aging populations, particularly through the integration of digital transformation initiatives [137-140].

The cost-effectiveness of telemedicine has been analyzed in multiple primary studies for a diverse array of medical conditions and specialties [146-149]. For instance, according to a study published in 2023 by Patel and colleagues [150], where the estimated cost savings of using telehealth among patients with cancer was evaluated, it was found that the estimated mean (SD) total cost savings ranged from US $147.4 (US $120.1) at US $0.56/mile (US $0.348/km) to US $186.1 (US $156.9) at US $0.82/mile (US $0.510/km). Likewise, in a study performed in a cost and effectiveness study of outpatients pulmonary care center in a rural area in Wisconsin (United States), the authors reported that telemedicine was found to be more cost-effective (US $335 per patient per year) compared to routine care (US $585 per patient per year) and on-site care (US $1166 per patient per year) [151]. When a sensitivity analysis was carried out, it revealed that the cost-effectiveness of telemedicine was sensitive to changes in the values for the number of patients, probability of successful telemedicine consultation, telemedicine equipment cost, utility of telemedicine, and percentage effort assigned to the on-site pulmonary physician [151]. Our data also suggested that telemedicine’s economic impact was substantial (with moderate certainty of evidence) due to its relative potential to decrease health care costs and increase accessibility. Another critical factor to consider in the cost-effectiveness model is the reduction in the need for in-person visits, along with the associated travel expenses [152,153]. This benefit is especially significant in resource-limited regions where in-person dementia care services are often inaccessible or precarious. Finally, telemedicine holds significant potential to deliver high-quality services and ensure the continuous provision of care. Together, these capabilities contribute to advancing the WHO’s goals of promoting equitable and universal access to health care. It is worthwhile mentioning that despite demonstrating cost benefits related to the use of remote interventions in dementia care, the development of robust cost-effectiveness studies and frameworks is still required, providing relevant insights for health care policy makers involved in advocating sustainable health care strategies across multiple regions in the globe [152-155].

Although we extracted and processed all barriers and facilitators mentioned in the included reviews, we chose to present them narratively due to the extensive body of literature that has emerged in these domains over recent years. Overall, the adoption of telemedicine and telehealth in dementia care is mediated by multiple components. Our findings suggested important barriers to the access and use of remote intervention for both patients and caregivers, including the need for in-person interactions, privacy and security concerns, technological challenges (eg, high-speed internet and device accessibility), and digital literacy gaps, which are particularly more challenging for older groups. In addition, geographic and accessibility limitations play a significant role in the hall of barriers, essentially in rural areas, complicating the delivery of telemedicine solutions due to multiple factors (ie, lack of infrastructure, limited connectivity, and lack of required technical support and professional training) [139,156]. On the other hand, our summary of facilitators that foster telemedicine uptake includes a wide range of modulators, such as enhanced health care accessibility, the convenience of remote care, and potential cost savings. Moreover, some reviews emphasized that training and educational activities prior to the large-scale and realistic implementation of interventions, availability of continuous technical support, and willingness to use the technologies are critical for effective use of these interventions.

Our overview of both scoping and systematic reviews included a considerable number of reviews, assessing the impact of telemedicine and telehealth solutions based on a complex and comprehensive number of relevant domains in dementia care. As is standard practice in our group, we adhered to established methodological requirements, including registering a protocol prior to project initiation, conducting a comprehensive literature search, providing justification for excluding shortlisted studies, using appropriate synthesis methods, and addressing potential biases that could influence the results. In addition, we used the AMSTAR 2 tool and GRADE-CERQual methodology to ensure the generation of high-quality evidence, emphasizing reliability, academic rigor, and methodological robustness. However, it is equally important to acknowledge and discuss the limitations of our study. First, our overview included patients with a diverse subtype of dementia, including Alzheimer disease, vascular dementia, Lewy body dementia, and frontotemporal dementia, which limited our work in terms of heterogeneity. This may obscure definitive conclusions and recommendations derived from our study. Furthermore, inconsistencies and variability in the quality of reporting across the included reviews introduced a degree of uncertainty in several outcomes assessed during the quality evaluation phase. This ultimately led to the downgrading of the certainty of evidence for all 10 primary outcomes. Despite these limitations, our overview offers an innovative perspective on the potential of telemedicine in dementia care. It identifies actionable strengths and critical knowledge gaps, serving as a foundation for generating higher-quality evidence in the fields of neurology, psychiatry, geriatrics, and gerontology, as well as telemedicine and telehealth.

### Conclusion

In conclusion, the results of this review underscore the transformative potential of telemedicine and telehealth in addressing the complex challenges of dementia care. Our comprehensive analysis revealed predominantly positive outcomes from the use of remote interventions across psychological, clinical, social, and economic outcomes. These findings are crucial for enhancing equality, accessibility, and sustainability in dementia care across diverse social and health care contexts. However, while the results are promising, they also emphasize the urgent need to address the heterogeneity in methodological quality and reporting across systematic and scoping reviews. The premises supported by this research are both viable and impactful, with the potential to be widely applicable. To fully achieve their potential as an integrated approach that overcomes multiple barriers, telemedicine and telehealth must be envisioned and implemented.

## Supplementary material

10.2196/75266 Multimedia Appendix 1 Search strategy, list of excluded studies, and additional outcome summary.

10.2196/75266 Multimedia Appendix 2 Primary information on included systematic and scoping reviews.

10.2196/75266 Multimedia Appendix 3 Reporting completeness among included systematic reviews using the AMSTAR 2 tool.

10.2196/75266 Checklist 1 PRISMA checklist.

## Abbreviations

AMSTAR 2: A Measurement Tool to Assess Systematic Reviews
DHT: digital health technology
*DSM-5*: *Diagnostic and Statistical Manual of Mental Disorders, Fifth Edition*
GRADE-CERQual: Confidence in the Evidence from Reviews of Qualitative Research
*ICD-10*: *International Statistical Classification of Diseases and Related Health Problems, Tenth Revision*
*ICD-11*: *International Classification of Diseases, 11th Revision*
JADE: Joint Action Addressing Dementia and Health
PRISMA: Preferred Reporting Items for Systematic Reviews and Meta-Analyses
RFO: relative frequency of occurrence
TAME: Thematic Analysis in Meta-Evidence
WHO: World Health Organization
