# Supplementary material for: An Overview of Reviews on Telemedicine and Telehealth in Dementia Care: Mixed Methods Synthesis
Source: JMIR Ment Health. 2025 Nov 6;12:e75266. doi: 10.2196/75266 (PMC12975415; doi:10.2196/75266)
Supplement: Multimedia Appendix 1 [file mental-v12-e75266-s001.docx]

**Multimedia Appendix 1**

**An Overview of Reviews on Telemedicine and Telehealth in Dementia Care: Mixed Methods Synthesis**

This supplementary material has been prepared by the research team to give additional and complementary information about the research project.

Table of Contents

[Supplementary Appendix 1 – Search strategies 3](#_Toc210583174)

[Embase 3](#_Toc210583175)

[MEDLINE^®^ 6](#_Toc210583176)

[Cochrane Library 9](#_Toc210583177)

[Scopus^®^ 12](#_Toc210583178)

[Epistemonikos 14](#_Toc210583179)

[Supplementary Appendix 2 – Reasons for excluding shortlisted studies 16](#_Toc210583180)

[Supplementary Appendix 3 – Additional Findings Relevant for Reporting 19](#_Toc210583181)

[Impact of telemedicine and telehealth on financial-related outcomes 19](#_Toc210583182)

[Impact of telemedicine on safety-related outcomes 20](#_Toc210583183)

[Impact of telemedicine on habitual physical activity metrics 21](#_Toc210583184)

[Impact of telemedicine on hospitalization 21](#_Toc210583185)

[Impact of telemedicine on motor progression in both a clinical and research capacity 22](#_Toc210583186)

# Supplementary Appendix 1 – Search strategies

The search was conducted on 20 February 2024

Search details for Comprehensive Overview of Digital Health Interventions for Dementia: A Synthesis of Systematic and Scoping Reviews Across Prevention, Diagnosis, Treatment, and Care Support Team

By: Lasse Østengaard - Bibliographic databases: Embase (Ovid), Medline (Ovid), Cochrane Database of Systematic Reviews (Cochrane Library), Scopus, and Epistemonikos.

## Embase

| **Embase Classic+Embase <1947 to 2024 February 19>** | | |
| --- | --- | --- |
| **#** | **Search Term** | **Hits** |
| 1 | exp dementia/ | 464683 |
| 2 | exp delirium/ | 45480 |
| 3 | dement*.mp. | 271791 |
| 4 | deliri*.mp. | 51928 |
| 5 | alzheimer*.mp. | 315313 |
| 6 | (lewy* adj2 bod*).mp. | 23785 |
| 7 | (creutzfeldt or cjd).mp. | 14767 |
| 8 | huntington*.mp. | 39820 |
| 9 | major neurocognitive disorder*.mp. | 546 |
| 10 | binswanger*.mp. | 1145 |
| 11 | korsako*.mp. | 3473 |
| 12 | (pick* adj2 disease).mp. | 8518 |
| 13 | (cerebr* adj2 deteriorat*).mp. | 436 |
| 14 | (frontotemporal adj2 degenerat*).mp. | 5724 |
| 15 | 1 or 2 or 3 or 4 or 5 or 6 or 7 or 8 or 9 or 10 or 11 or 12 or 13 or 14 | 608030 |
| 16 | exp telehealth/ | 92381 |
| 17 | (telemedicine or telehealth or telemonitoring or remote monitoring).mp. | 85978 |
| 18 | (mhealth or m-health or ehealth or e-health or emental or e-mental or e-prescri*).mp. | 23727 |
| 19 | ((Medical or health) adj3 monitor*).mp. | 26142 |
| 20 | exp medical informatics/ | 23893 |
| 21 | ((Medical or biomedical or health or clinical) adj3 informatics).mp. | 30096 |
| 22 | exp technology/ | 279270 |
| 23 | (technolog* or digital*).mp. | 1313204 |
| 24 | exp internet/ | 132477 |
| 25 | computer assisted therapy/ or personal digital assistant/ | 6715 |
| 26 | ((internet or online or web* or tele* or computer or cyberspace or cyber space or remote or virtual or electronic) adj3 (consult* or based or application* or intervention* or program* or therap* or rehabilitat* or prescri*)).mp. | 419435 |
| 27 | exp mobile phone/ or exp mobile application/ | 68481 |
| 28 | (app or apps).mp. | 64557 |
| 29 | (mobile* or portable or phone* or telephone* or smartphone* or cellphone* or smartwatch* or screen or touchscreen or multimedia or multi media).mp. | 720663 |
| 30 | (Gaming or game or exergam*).mp. | 53854 |
| 31 | robot*.mp. | 128202 |
| 32 | (Connected Devices or Smart Devices or Digital Assistant).mp. | 3950 |
| 33 | electronic medical record system/ or electronic medical record/ or exp hospital information system/ | 114434 |
| 34 | ((Health or medical or hospital) adj3 (record* or informati* or data or science)).mp. | 958545 |
| 35 | ((Clinical or medical) adj3 (alert or reminder)).mp. | 1812 |
| 36 | (personalized medicine or personalised medicine or interoperability or decision support).mp. | 139161 |
| 37 | exp social media/ | 52761 |
| 38 | (social media or social network* or Facebook or twitter or youtube or Instagram or flickr or Linkedin or blog* or on-line communit* or online communit* or wiki*).mp. | 106586 |
| 39 | (surveillance or GPS or track* or position* or sensor* or big data or open data or cloud or 4G* or 5G* or bluetooth or wearable* or wireless).mp. | 2382949 |
| 40 | (home adj6 (automat* or monitor*)).mp. | 18043 |
| 41 | exp artificial intelligence/ | 95956 |
| 42 | ((Artificial or machine or deep or hierarchical or ambient or comput* or assist*) adj3 (intelligence or learning)).mp. | 264711 |
| 43 | (Computer adj3 (vision or knowledge)).mp. | 11288 |
| 44 | (Knowledge adj3 (acquisition or representation*)).mp. | 7673 |
| 45 | natural language processing.mp. | 14296 |
| 46 | (AI or NLP).mp. | 79604 |
| 47 | ((Computer or automated) adj3 reasoning).mp. | 249 |
| 48 | 16 or 17 or 18 or 19 or 20 or 21 or 22 or 23 or 24 or 25 or 26 or 27 or 28 or 29 or 30 or 31 or 32 or 33 or 34 or 35 or 36 or 37 or 38 or 39 or 40 or 41 or 42 or 43 or 44 or 45 or 46 or 47 | 5870583 |
| 49 | exp "systematic review"/ or exp meta analysis/ | 583407 |
| 50 | exp "systematic review (topic)"/ or exp "meta analysis (topic)"/ | 75008 |
| 51 | ((systematic or scoping) adj3 (review* or overview*)).ti,ab. | 425079 |
| 52 | (meta analy* or metaanaly* or meta-analy*).ti,ab. | 373615 |
| 53 | 49 or 50 or 51 or 52 | 787543 |
| 54 | 15 and 48 and 53 | 3576 |

## MEDLINE^®^

| Ovid MEDLINE(R) ALL <1946 to February 19, 2024> | | |
| --- | --- | --- |
| **#** | **Search Term** | **Hits** |
| 1 | exp Dementia/ | 212406 |
| 2 | exp Delirium/ | 13216 |
| 3 | dement*.mp. | 171747 |
| 4 | deliri*.mp. | 25656 |
| 5 | alzheimer*.mp. | 211169 |
| 6 | (lewy* adj2 bod*).mp. | 13128 |
| 7 | (creutzfeldt or cjd).mp. | 9368 |
| 8 | huntington*.mp. | 23245 |
| 9 | major neurocognitive disorder*.mp. | 359 |
| 10 | binswanger*.mp. | 609 |
| 11 | korsako*.mp. | 1872 |
| 12 | (pick* adj2 disease).mp. | 4569 |
| 13 | (cerebr* adj2 deteriorat*).mp. | 268 |
| 14 | (frontotemporal adj2 degenerat*).mp. | 3960 |
| 15 | 1 or 2 or 3 or 4 or 5 or 6 or 7 or 8 or 9 or 10 or 11 or 12 or 13 or 14 | 373909 |
| 16 | exp Telemedicine/ | 46711 |
| 17 | (telemedicine or telehealth or telemonitoring or remote monitoring).mp. | 61478 |
| 18 | (mhealth or m-health or ehealth or e-health or emental or e-mental or e-prescri*).mp. | 22402 |
| 19 | ((Medical or health) adj3 monitor*).mp. | 21804 |
| 20 | exp Medical Informatics/ | 504353 |
| 21 | ((Medical or biomedical or health or clinical) adj3 informatics).mp. | 23847 |
| 22 | exp Technology/ | 505857 |
| 23 | (technolog* or digital*).mp. | 976394 |
| 24 | exp Internet/ | 99699 |
| 25 | exp Computers, Handheld/ or exp Therapy, Computer-Assisted/ | 60062 |
| 26 | ((internet or online or web* or tele* or computer or cyberspace or cyber space or remote or virtual or electronic) adj3 (consult* or based or application* or intervention* or program* or therap* or rehabilitat* or prescri*)).mp. | 197464 |
| 27 | exp Cell Phone/ or Mobile Applications/ | 31484 |
| 28 | (app or apps).mp. | 47937 |
| 29 | (mobile* or portable or phone* or telephone* or smartphone* or cellphone* or smartwatch* or screen or touchscreen or multimedia or multi media).mp. | 502368 |
| 30 | (Gaming or game or exergam*).mp. | 42889 |
| 31 | robot*.mp. | 82661 |
| 32 | (Connected Devices or Smart Devices or Digital Assistant).mp. | 2172 |
| 33 | exp Medical Records Systems, Computerized/ or exp hospital information system/ | 75418 |
| 34 | ((Health or medical or hospital) adj3 (record* or informati* or data or science)).mp. | 497187 |
| 35 | ((Clinical or medical) adj3 (alert or reminder)).mp. | 1136 |
| 36 | (personalized medicine or personalised medicine or interoperability or decision support).mp. | 75088 |
| 37 | exp Social Media/ | 16931 |
| 38 | (social media or social network* or Facebook or twitter or youtube or Instagram or flickr or Linkedin or blog* or on-line communit* or online communit* or wiki*).mp. | 73158 |
| 39 | (surveillance or GPS or track* or position* or sensor* or big data or open data or cloud or 4G* or 5G* or bluetooth or wearable* or wireless).mp. | 1806531 |
| 40 | (home adj6 (automat* or monitor*)).mp. | 9609 |
| 41 | exp Artificial Intelligence/ | 190243 |
| 42 | ((Artificial or machine or deep or hierarchical or ambient or comput* or assist*) adj3 (intelligence or learning)).mp. | 213802 |
| 43 | (Computer adj3 (vision or knowledge)).mp. | 9787 |
| 44 | (Knowledge adj3 (acquisition or representation*)).mp. | 6331 |
| 45 | natural language processing.mp. | 12132 |
| 46 | (AI or NLP).mp. | 59165 |
| 47 | ((Computer or automated) adj3 reasoning).mp. | 211 |
| 48 | 16 or 17 or 18 or 19 or 20 or 21 or 22 or 23 or 24 or 25 or 26 or 27 or 28 or 29 or 30 or 31 or 32 or 33 or 34 or 35 or 36 or 37 or 38 or 39 or 40 or 41 or 42 or 43 or 44 or 45 or 46 or 47 | 4640018 |
| 49 | exp "systematic review"/ or exp meta-analysis/ | 338887 |
| 50 | exp Systematic Reviews as Topic/ or exp Meta-Analysis as Topic/ | 37194 |
| 51 | ((systematic or scoping) adj3 (review* or overview*)).ti,ab. | 355144 |
| 52 | (meta analy* or metaanaly* or meta-analy*).ti,ab. | 296214 |
| 53 | 49 or 50 or 51 or 52 | 526807 |
| 54 | 15 and 48 and 53 | 2017 |

## Cochrane Library

| **#** | **Search Term** | **Hits** |
| --- | --- | --- |
| ID | Search | Hits |
| #1 | MeSH descriptor: [Dementia] explode all trees | 9235 |
| #2 | MeSH descriptor: [Delirium] explode all trees | 1533 |
| #3 | (dement*):ti,ab,kw | 17753 |
| #4 | (deliri*):ti,ab,kw | 5745 |
| #5 | (alzheimer*):ti,ab,kw | 14169 |
| #6 | (lewy* NEAR/1 bod*):ti,ab,kw | 538 |
| #7 | (creutzfeldt or cjd):ti,ab,kw | 69 |
| #8 | (huntington*):ti,ab,kw | 815 |
| #9 | (major neurocognitive disorder*):ti,ab,kw | 761 |
| #10 | (binswanger*):ti,ab,kw | 9 |
| #11 | (korsako*):ti,ab,kw | 79 |
| #12 | (pick* NEAR/1 disease):ti,ab,kw | 129 |
| #13 | (cerebr* NEAR/1 deteriorat*):ti,ab,kw | 11 |
| #14 | (frontotemporal NEAR/1 degenerat*):ti,ab,kw | 18 |
| #15 | #1 or #2 or #3 or #4 or #5 or #6 or #7 or #8 or #9 or #10 or #11 or #12 or #13 or #14 | 32251 |
| #16 | MeSH descriptor: [Telemedicine] explode all trees | 4744 |
| #17 | (telemedicine or telehealth or telemonitoring or "remote monitoring"):ti,ab,kw | 10681 |
| #18 | (mhealth or m-health or ehealth or e-health or emental or e-mental or e-prescri*):ti,ab,kw | 4400 |
| #19 | ((Medical or health) NEAR/2 monitor*):ti,ab,kw | 1695 |
| #20 | MeSH descriptor: [Medical Informatics] explode all trees | 14699 |
| #21 | ((Medical or biomedical or health or clinical) NEAR/2 informatics):ti,ab,kw | 458 |
| #22 | MeSH descriptor: [Technology] explode all trees | 9050 |
| #23 | (technolog* or digital*):ti,ab,kw | 51579 |
| #24 | MeSH descriptor: [Internet] explode all trees | 6471 |
| #25 | MeSH descriptor: [Computers, Handheld] explode all trees | 1525 |
| #26 | MeSH descriptor: [Therapy, Computer-Assisted] explode all trees | 3190 |
| #27 | ((internet or online or web* or tele* or computer or cyberspace or "cyber space" or remote or virtual or electronic) NEAR/2 (consult* or based or application* or intervention* or program* or therap* or rehabilitat* or prescri*)):ti,ab,kw | 40253 |
| #28 | MeSH descriptor: [Cell Phone] explode all trees | 3421 |
| #29 | MeSH descriptor: [Mobile Applications] this term only | 1898 |
| #30 | (app or apps):ti,ab,kw | 10674 |
| #31 | (mobile* or portable or phone* or telephone* or smartphone* or cellphone* or smartwatch* or screen or touchscreen or multimedia or "multi media"):ti,ab,kw | 71986 |
| #32 | (gaming or game or exergam*):ti,ab,kw | 6579 |
| #33 | (robot*):ti,ab,kw | 7450 |
| #34 | ("Connected Devices" or "Smart Devices" or "Digital Assistant"):ti,ab,kw | 251 |
| #35 | MeSH descriptor: [Medical Records Systems, Computerized] explode all trees | 1174 |
| #36 | MeSH descriptor: [Hospital Information Systems] explode all trees | 863 |
| #37 | ((Health or medical or hospital) NEAR/2 (record* or informati* or data or science)):ti,ab,kw | 31013 |
| #38 | ((Clinical or medical) NEAR/2 (alert or reminder)):ti,ab,kw | 97 |
| #39 | ("personalized medicine" or "personalised medicine" or interoperability or "decision support"):ti,ab,kw | 6263 |
| #40 | MeSH descriptor: [Social Media] explode all trees | 562 |
| #41 | ("social media" or "social network" or "social networks" or Facebook or twitter or youtube or Instagram or flickr or Linkedin or blog* or "on-line community" or "on-line communities" or "online community" or "online communities" or wiki*):ti,ab,kw | 5994 |
| #42 | (surveillance or GPS or track* or position* or sensor* or "big data" or "open data" or cloud or 4G* or 5G* or bluetooth or wearable* or wireless):ti,ab,kw | 104924 |
| #43 | (home NEAR/5 (automat* or monitor*)):ti,ab,kw | 2799 |
| #44 | MeSH descriptor: [Artificial Intelligence] explode all trees | 3108 |
| #45 | ((Artificial or machine or deep or hierarchical or ambient or comput* or assist*) NEAR/2 (intelligence or learning)):ti,ab,kw | 5432 |
| #46 | (Computer NEAR/2 (vision or knowledge)):ti,ab,kw | 211 |
| #47 | (Knowledge NEAR/2 (acquisition or representation*)):ti,ab,kw | 502 |
| #48 | ("natural language processing"):ti,ab,kw | 256 |
| #49 | (AI or NLP):ti,ab,kw | 5911 |
| #50 | ((Computer or automated) NEAR/2 reasoning):ti,ab,kw | 7 |
| #51 | #16 or #17 or #18 or #19 or #20 or #21 or #22 or #23 or #24 or #25 or #26 or #27 or #28 or #29 or #30 or #31 or #32 or #33 or #34 or #35 or #36 or #37 or #38 or #39 or #40 or #41 or #42 or #43 or #44 or #45 or #46 or #47 or #48 or #49 or #50 | 290278 |
| #52 | #15 and #51 | 5795 |
| #53 | #52 in Cochrane Reviews | 74 |

## Scopus^®^

| **#** | **Search Term** | **Hits** |
| --- | --- | --- |
| 1 | ( ( TITLE-ABS-KEY ( ( dement* OR deliri* OR alzheimer* OR creutzfeldt OR cjd OR huntington* OR binswanger* OR korsako* OR "major neurocognitive disorder" OR "major neurocognitive disorders" ) ) ) OR ( TITLE-ABS-KEY ( ( lewy* W/2 bod* ) ) ) OR ( TITLE-ABS-KEY ( ( pick* W/2 disease ) ) ) OR ( TITLE-ABS-KEY ( ( cerebr* W/2 deteriorat* ) ) ) OR ( TITLE-ABS-KEY ( ( frontotemporal W/2 degenerat* ) ) ) ) | 528,981 |
| 2 | ( ( TITLE-ABS-KEY ( ( computer OR automated ) W/2 reasoning ) ) OR ( TITLE-ABS-KEY ( ai OR nlp ) ) OR ( TITLE-ABS-KEY ( "natural language processing" ) ) OR ( TITLE-ABS-KEY ( knowledge W/2 ( acquisition OR representation* ) ) ) OR ( TITLE-ABS-KEY ( computer W/2 ( vision OR knowledge ) ) ) OR ( TITLE-ABS-KEY ( ( artificial OR machine OR deep OR hierarchical OR ambient OR comput* OR assist* ) W/2 ( intelligence OR learning ) ) ) OR ( TITLE-ABS-KEY ( home W/5 ( automat* OR monitor* ) ) ) OR ( TITLE-ABS-KEY ( surveillance OR gps OR track* OR position* OR sensor* OR "big data" OR "open data" OR cloud OR 4g* OR 5g* OR bluetooth OR wearable* OR wireless ) ) OR ( TITLE-ABS-KEY ( "social media" OR "social network" OR "social networks" OR facebook OR twitter OR youtube OR instagram OR flickr OR linkedin OR blog* OR "on-line community" OR "on-line communities" OR "online community" OR "online communities" OR wiki* ) ) OR ( TITLE-ABS-KEY ( "personalized medicine" OR "personalised medicine" OR interoperability OR "decision support" ) ) OR ( TITLE-ABS-KEY ( ( clinical OR medical ) W/2 ( alert OR reminder ) ) ) OR ( TITLE-ABS-KEY ( ( clinical OR medical ) W/2 ( alert OR reminder ) ) ) OR ( TITLE-ABS-KEY ( ( health OR medical OR hospital ) W/2 ( record* OR informati* OR data OR science ) ) ) OR ( TITLE-ABS-KEY ( robot* ) ) OR ( TITLE-ABS-KEY ( gaming OR game OR exergam* ) ) OR ( TITLE-ABS-KEY ( mobile* OR portable OR phone* OR telephone* OR smartphone* OR cellphone* OR smartwatch* OR screen OR touchscreen OR multimedia OR "multi media" ) ) OR ( TITLE-ABS-KEY ( app OR apps ) ) OR ( TITLE-ABS-KEY ( ( internet OR online OR web* OR tele* OR computer OR cyberspace OR "cyber space" OR remote OR virtual OR electronic ) W/2 ( consult* OR based OR application* OR intervention* OR program* OR therap* OR rehabilitat* OR prescri* ) ) ) OR ( TITLE-ABS-KEY ( technolog* OR digital* ) ) OR ( TITLE-ABS-KEY ( ( medical OR biomedical OR health OR clinical ) W/2 informatics ) ) OR ( TITLE-ABS-KEY ( telemedicine OR telehealth OR telemonitoring OR remote AND monitoring ) ) OR ( TITLE-ABS-KEY ( mhealth OR m-health OR ehealth OR e-health OR emental OR e-mental OR e-prescri* ) ) OR ( TITLE-ABS-KEY ( ( medical OR health ) W/2 monitor* ) ) ) | 16,489,948 |
| 3 | ( ( TITLE-ABS-KEY ( ( meta PRE/ analy* ) OR metaanaly* OR meta-analy* ) ) OR ( TITLE-ABS-KEY ( ( systematic OR scoping ) W/2 ( review* OR overview* ) ) ) ) | 773,373 |
| 4 | 1 AND 2 AND 3 | 3,104 |

## Epistemonikos

| **#** | **Search Term** | **Hits** |
| --- | --- | --- |
| 1 | (title:(dement* OR deliri* OR alzheimer* OR huntington* OR binswanger* OR korsako* OR creutzfeldt OR cjd OR lewy* OR pick* OR "major neurocognitive disorder" OR "major neurocognitive disorders" OR "cerebral deterioration" OR "frontotemporal degeneration") OR abstract:(dement* OR deliri* OR alzheimer* OR huntington* OR binswanger* OR korsako* OR creutzfeldt OR cjd OR lewy* OR pick* OR "major neurocognitive disorder" OR "major neurocognitive disorders" OR "cerebral deterioration" OR "frontotemporal degeneration")) | 46,451 |
| 2 | (title:(digital OR technolog* OR telemedicine OR telehealth OR telemonitoring OR "remote monitoring" OR mhealth OR m-health OR ehealth OR "e-health" OR "health technologies" OR emental OR "e-mental" OR e-prescri* OR "medical monitoring" OR "health monitoring" OR "medical informatics" OR "biomedical informatics" OR "health informatics" OR "clinical informatics" OR internet OR app OR apps OR mobile* OR portable OR phone* OR telephone* OR smartphone* OR cellphone* OR smartwatch* OR screen OR touchscreen OR multimedia OR "multi media" OR gaming OR game OR exergam* OR robot* OR "connected devices" OR "smart devices" OR "clinical alert" OR "clinical reminder" OR "medical alert" OR "medical reminder" OR "personalized medicine" OR "personalised medicine" OR interoperability OR "decision support" OR "social media" OR "social network" OR "social networks" OR surveillance OR GPS OR track* OR position* OR sensor* OR "big data" OR "open data" OR cloud OR 4G* OR 5G* OR bluetooth OR wearable* OR wireless OR AI OR NLP OR "artificial intelligence" OR "machine learning" OR "natural language processing" OR "online consultation" OR "online consultations" OR "electronic consultation" OR "electronic consultations" OR "remote consultation" OR "remote consultations" OR "online intervention" OR "online therapy" OR "online rehabilitation" OR "electronic prescription" OR "electronic prescribing") OR abstract:(digital OR technolog* OR telemedicine OR telehealth OR telemonitoring OR "remote monitoring" OR mhealth OR m-health OR ehealth OR "e-health" OR "health technologies" OR emental OR "e-mental" OR e-prescri* OR "medical monitoring" OR "health monitoring" OR "medical informatics" OR "biomedical informatics" OR "health informatics" OR "clinical informatics" OR internet OR app OR apps OR mobile* OR portable OR phone* OR telephone* OR smartphone* OR cellphone* OR smartwatch* OR screen OR touchscreen OR multimedia OR "multi media" OR gaming OR game OR exergam* OR robot* OR "connected devices" OR "smart devices" OR "clinical alert" OR "clinical reminder" OR "medical alert" OR "medical reminder" OR "personalized medicine" OR "personalised medicine" OR interoperability OR "decision support" OR "social media" OR "social network" OR "social networks" OR surveillance OR GPS OR track* OR position* OR sensor* OR "big data" OR "open data" OR cloud OR 4G* OR 5G* OR bluetooth OR wearable* OR wireless OR AI OR NLP OR "artificial intelligence" OR "machine learning" OR "natural language processing" OR "online consultation" OR "online consultations" OR "electronic consultation" OR "electronic consultations" OR "remote consultation" OR "remote consultations" OR "online intervention" OR "online therapy" OR "online rehabilitation" OR "electronic prescription" OR "electronic prescribing")) | 456,804 |
| 3 | (title:(((systematic OR scoping) AND (review* OR overview*)) OR ((meta analy*) OR metaanaly* OR meta-analy*)) OR abstract:(((systematic OR scoping) AND (review* OR overview*)) OR ((meta analy*) OR metaanaly* OR meta-analy*))) | 476,960 |
| 4 | 1 AND 2 AND 3 | 1065 |

#

# Supplementary Appendix 2 – Reasons for excluding shortlisted studies

| **Title** | **First Author** | **Year** | **Journal** | **Notes** |
| --- | --- | --- | --- | --- |
| Review of mobile phone use in preventive medicine and disease management | Skinner, C. | 2008 | Conf. Telehealth Assistive Technol., Telehealth/AT | 1 |
| Investigating the effectiveness of technologies applied to assist seniors: A systematic literature review | Khosravi P. | 2016 | International Journal of Medical Informatics | 1 |
| The management of urinary incontinence in nursing homes: a scoping review. | Ostaszkiewicz, Joan | 2023 | Australian & New Zealand Continence Journal | 1 |
| Tele-neurology in sub-Saharan Africa: A systematic review of the literature | Sarfo F.S. | 2017 | Journal of the Neurological Sciences | 1 |
| Collaborative Care for Psychiatric Disorders in Older Adults: A Systematic Review | Dham P. | 2017 | Canadian Journal of Psychiatry | 1 |
| Virtual models of care for people with palliative care needs living in their own home: A systematic meta-review and narrative synthesis | Disalvo D. | 2021 | Palliative Medicine | 1 |
| Telemedicine for neurologic diseases: A systematic review and meta-analysis. | Leon-Salas B. | 2023 | European journal of neurology | 1 |
| Consistency of dementia caregiver intervention classification: An evidence-based synthesis | Gaugler J.E. | 2017 | International Psychogeriatrics | 2 |
| What do we know about strategies to manage dementia-related wandering? A scoping review | Neubauer N.A. | 2018 | Alzheimer's and Dementia: Diagnosis, Assessment and Disease Monitoring | 2 |
| Clinical effectiveness of pharmacological and non-pharmacological treatments for the management of anxiety in community dwelling people living with dementia: A systematic review and meta-analysis | Nimmons D. | 2024 | Neuroscience and Biobehavioral Reviews | 2 |
| A systematic review of the use of technology for reminiscence therapy | Lazar A. | 2014 | Health education & behavior | 2 |
| Review: Non-pharmacological interventions delivered by family caregivers improve symptoms in people with dementia | Seitz, D.P. | 2013 | Evid. -Based Ment. Health | 2 |
| Alzheimer's early detection in post-acute COVID-19 syndrome: a systematic review and expert consensus on preclinical assessments | Vandersteen C. | 2023 | Frontiers in Aging Neuroscience | 2 |
| Multi-domain prognostic models used in middle-aged adults without known cognitive impairment for predicting subsequent dementia. | Mohanannair Geethadevi, Gopisankar | 2023 | The Cochrane database of systematic reviews | 2 |
| Exploring the impact of Covid-19 on the care and quality of life of people with dementia and their carers: A scoping review | Masterson-Algar, P. | 2022 | Dementia | 2 |
| Factors Concerning Veterans With Dementia, Their Caregivers, and Coordination of Care: A Systematic Literature Review | Mileski M. | 2017 | Military medicine | 2 |
| A rapid review of internet mediated research methods with people with dementia: Practical, technical, and ethical considerations | Phenwan, T. | 2021 | Qual. Rep. | 2 |
| Exploring assistive technology as a potential beneficial intervention tool for people with alzheimer's disease - a systematic review | Klimova B. | 2018 | Neuropsychiatric Disease and Treatment | 2 |
| Psychosocial intervention for carers of people with dementia: What components are most effective and when? A systematic review of systematic reviews | Dickinson C. | 2017 | International Psychogeriatrics | 2 |
| Factors affecting information technology use from the perspective of aging persons with cognitive disabilities: A scoping review of qualitative research | Rocheleau J.N. | 2020 | Technology and Disability | 2 |
| Using health information technology in residential aged care homes: An integrative review to identify service and quality outcomes | Bail K. | 2022 | International Journal of Medical Informatics | 3 |
| The effectiveness of interventions in supporting self-management of informal caregivers of people with dementia; A systematic meta review Health services research | Huis In Het Veld, J.G. | 2015 | BMC Geriatr. | 3 |
| 1: No focus on Dementia; 2: No focus on Telemedicine; 3: Not systematic or scoping review | | | | |

# Supplementary Appendix 3 – Additional Findings Relevant for Reporting

## Impact of telemedicine and telehealth on financial-related outcomes

- Overall impact of telemedicine and telehealth interventions evidenced the potential cost-effectiveness, with being reported in 27 reviews^(4,6,7,12,15,16,23,26,30,31,33,34,37,42,44-46,58,69,79,80,83,86-89)^ (RFO of 27.0% (95% CI 21.0 to 40.0); weight of 10.3%).
- Most reviews showed the importance of telemedicine in decreasing travel-related expenses, which ultimately benefits end-users and enhances their accessibility via the use of remote consultation platforms (particularly important among those individuals living in remote or rural regions).
- Some reviews showed the cost-saving feature inherent to the use of telemedicine/telehealth services as compared to regular care. For example, one study showed a decrease in consultation expenditure by up to 85·7% when telemedicine has been taken up and utilized instead of in-person medical appointment visits (costs of €3.74 per telephone consultation compared to €86.87 for face-to-face interactions)^30^.
- Some reviews showed the importance of remote interventions in permitting caregivers to decrease commuting routinely back and forth from their homes to work.
- One review indicated that due to the utilization of remote interventions, caregiving-related financial burdens were decreased, which indirectly impacts their resilience and capacity to manage dementia care^15^.
- Not all reviews consistently and clearly reported cost benefits derived from telehealth/telemedicine systems; in some cases, these technologies were linked to enhanced time (such as in those cases with technical difficulties) or when complementary resources were needed.

*Impact of telemedicine on clinical improvement of underlying diseases (besides the ones related to neurological or dementia disorders)*

- Based on moderate evidence, some studies reported clinical improvement in managing underlying non-neurological and non-dementia-related conditions^(1,2,4,6,14,17,22,25,26,30,31,42,46,56,70,73,76,78,79,80,81,87,89)^ (RFO of 26·0% [95% CI 18·0 to 37·0]; weight of 9·9%)​.
- Potentially improved comorbidities documented chronic conditions, including chronic obstructive pulmonary disease (COPD) and heart failure.
- Only one study reported telehealth’s role in reducing hospital readmissions by better supporting patients’ self-care of underlying diseases.
- Some studies showed promising results regarding the use of telemedicine in postoperative care, specifically for patients recovering from major surgeries like cancer. These studies reported positive outcomes in accelerating recovery times, decreasing hospitalization rates and duration, and improving patient-reported outcomes (including psychological ones).
- Telehealth solutions fostered timely monitoring and adjustments in treatment plans, potentially preventing exacerbations of physical health conditions.

## Impact of telemedicine on safety-related outcomes

- Safety-related outcomes associated with telemedicine interventions were reported in 21 studies^(1,2,4,10,11,17,23,26,31,32,34,46,40,47,52,56,69,74,76,78,87)^, accounting for an RFO of 30.0% [95% CI 21.0 to 40.0] and a weight of 9.9% in the analysis.
- Telemedicine and telehealth applied to dementia care impacted safety by mitigating risks associated with in-person visits and enhancing patient monitoring. For example, systems utilizing telecare technology, such as GPS tracking and ambient sensors, permit professionals to monitor patients’ location, thereby decreasing risks of wandering and improving safety for patients with dementia.
- Telemedicine was also suggested to be a safe alternative for healthcare access, especially during the COVID-19 pandemic, by decreasing the need for physical contact, decreasing infection risks for vulnerable populations.

## Impact of telemedicine on habitual physical activity metrics

- Telemedicine was shown to positively impact habitual physical activity metrics among patients with dementia and related conditions, with an RFO of 29.0% [95% CI 20.0 to 38.0] and a weight of 9.9% ^(2,9,10,14,17,23,26,34,40,44,56,59,68,69,76,84,89)^.
- Telemedicine was reported to increase engagement in physical activities, a specialty based on the use of wearable devices and remote exercise classes.
- Some studies suggested the efficacy of individualized telehealth programs in fostering participation in physical activities, with adherence rates across interventions varying widely, from 25% to as high as 90%.
- One included record evidenced improvements in balance, limb strength, and walking performance through a smartphone application training plan that offered three to four sessions per week^56^.
- Despite the notable positive results, the effectiveness of telemedicine systems on physical activity presented some variability. A few included studies showed restricted or non-significant improvements, particularly in cases where physical activity support was unsupervised or relied heavily on self-monitoring.

## Impact of telemedicine on hospitalization

- Telemedicine interventions yielded mixed results in terms of hospitalization outcomes within dementia and related care, with an RFO of 18.0% [95% CI 12.0 to 25.0] and a weight of 8.7%^(31,50,57,72,77,86,89)^.
- Several studies showed that a reduction in emergency department (ED) visits and hospital readmissions correlated with telemedicine platforms. For example, one study reported a notable 33% reduction in ED visits and a 66% decrease in hospital readmissions in the intervention group compared to controls.
- Telemedicine was also reported to be effective in targeted behavioral and psychological symptoms of people living with dementia, such as managing agitation and noisy BPSD (behavioral and psychological symptoms of dementia).

## Impact of telemedicine on motor progression in both a clinical and research capacity

- This was the least frequently reported outcome ^(31,69)^, with a relative frequency of occurrence (RFO) of 2.0% [95% CI 1.0 to 4.0] and a weight of 7.3%.
- The restricted amount of data extracted showed the relative scarcity of research in this area, especially related to motor function appraisal and intervention for dementia-related symptoms.
- A limited number of studies suggested the potential applicability and utility of wearable systems and remote monitoring technologies to track motor progression (e.g., body posture, gait, and movement).

Reference list of the 91 included studies

1. Di Lorito C, Bosco A, Rai H, et al. A systematic literature review and meta-analysis on digital health interventions for people living with dementia and mild cognitive impairment. Int J Geriatr Psychiatry. Jun 2022;37(6). [doi: 10.1002/gps.5730] [Medline: 35588315]
2. Daly Lynn J, Rondón-Sulbarán J, Quinn E, Ryan A, McCormack B, Martin S. A systematic review of electronic assistive technology within supporting living environments for people with dementia. Dementia (London). 2019;18(7-8):2371-2435. [doi: 10.1177/1471301217733649] [Medline: 28990408]
3. Dam AEH, de Vugt ME, Klinkenberg IPM, Verhey FRJ, van Boxtel MPJ. A systematic review of social support interventions for caregivers of people with dementia: are they doing what they promise? Maturitas. Mar 2016;85:117-130. [doi: 10.1016/j.maturitas.2015.12.008] [Medline: 26857890]
4. Elbaz S, Cinalioglu K, Sekhon K, et al. A systematic review of telemedicine for older adults with dementia during COVID-19: an alternative to in-person health services? Front Neurol. 2021;12:761965. [doi: 10.3389/fneur.2021.761965] [Medline: 34970210]
5. Jackson D, Roberts G, Wu ML, Ford R, Doyle C. A systematic review of the effect of telephone, internet or combined support for carers of people living with Alzheimer’s, vascular or mixed dementia in the community. Arch Gerontol Geriatr. 2016;66:218-236. [doi: 10.1016/j.archger.2016.06.013] [Medline: 27372903]
6. Martin-Khan M, Wootton R, Gray L. A systematic review of the reliability of screening for cognitive impairment in older adults by use of standardised assessment tools administered via the telephone. J Telemed Telecare. 2010;16(8):422-428. [doi: 10.1258/jtt.2010.100209] [Medline: 21030488]
7. Riley CO, McKinstry B, Fairhurst K. Accuracy of telephone screening tools to identify dementia patients remotely: systematic review. JRSM Open. Sep 2022;13(9):20542704221115956. [doi: 10.1177/20542704221115956] [Medline: 36082188]
8. Elliott E, Green C, Llewellyn DJ, Quinn TJ. Accuracy of telephone-based cognitive screening tests: systematic review and meta-analysis. Curr Alzheimer Res. 2020;17(5):460-471. [doi: 10.2174/1567205017999200626201121] [Medline: 32589557]
9. van der Wardt V, Hancox J, Gondek D, et al. Adherence support strategies for exercise interventions in people with mild cognitive impairment and dementia: a systematic review. Prev Med Rep. Sep 2017;7:38-45. [doi: 10.1016/j.pmedr.2017.05.007] [Medline: 28593121]
10. Martinez-Alcala CI, Pliego-Pastrana P, Lopez-Noguerola JS, Rosales-Lagarde A, Zaleta-Arias ME. Adoption of ICT in the aging: systematic review based on ICT for Alzheimer’s disease and other senile dementias. Presented at: 2015 10th Iberian Conference on Information Systems and Technologies (CISTI); Jun 17-20, 2015; Aveiro, Portugal. [doi: 10.1109/CISTI.2015.7170393]
11. Gagnon-Roy M, Bourget A, Stocco S, Courchesne ACL, Kuhne N, Provencher V. Assistive technology addressing safety issues in dementia: a scoping review. Am J Occup Ther. 2017;71(5):7105190020p1-7105190020p10. [doi: 10.5014/ajot.2017.025817] [Medline: 28809655]
12. Kwan RYC, Lai CKY. Can smartphones enhance telephone-based cognitive assessment (TBCA)? Int J Environ Res Public Health. Dec 12, 2013;10(12):7110-7125. [doi: 10.3390/ijerph10127110] [Medline: 24351736]
13. Lin X, Ward SA, Pritchard E, et al. Carer-reported measures for a dementia registry: a systematic scoping review and a qualitative study. Australas J Ageing. Mar 2023;42(1):34-52. [doi: 10.1111/ajag.13148] [Medline: 36383194]
14. Cotelli M, Manenti R, Brambilla M, et al. Cognitive telerehabilitation in mild cognitive impairment, Alzheimer’s disease and frontotemporal dementia: a systematic review. J Telemed Telecare. Feb 2019;25(2):67-79. [doi: 10.1177/1357633X17740390] [Medline: 29117794]
15. Dedzoe JDS, Malmgren Fänge A, Christensen J, Lethin C. Collaborative learning through a virtual community of practice in dementia care support: a scoping review. Healthcare (Basel). Feb 26, 2023;11(5):692. [doi: 10.3390/healthcare11050692] [Medline: 36900696]
16. Waller A, Dilworth S, Mansfield E, Sanson-Fisher R. Computer and telephone delivered interventions to support caregivers of people with dementia: a systematic review of research output and quality. BMC Geriatr. Nov 16, 2017;17(1):265. [doi: 10.1186/s12877-017-0654-6] [Medline: 29145806]
17. Maggio MG, De Bartolo D, Calabrò RS, et al. Computer-assisted cognitive rehabilitation in neurological patients: state-of-art and future perspectives. Front Neurol. 2023;14:1255319. [doi: 10.3389/fneur.2023.1255319] [Medline: 37854065]
18. Piau A, Wild K, Mattek N, Kaye J. Current state of digital biomarker technologies for real-life, home-based monitoring of cognitive function for mild cognitive impairment to mild Alzheimer disease and implications for clinical care: systematic review. J Med Internet Res. Aug 30, 2019;21(8):e12785. [doi: 10.2196/12785] [Medline: 31471958]
19. Gaigher JM, Lacerda IB, Dourado MCN. Dementia and mental health during the COVID-19 pandemic: a systematic review. Front Psychiatry. 2022;13:879598. [doi: 10.3389/fpsyt.2022.879598] [Medline: 35873228]
20. Morgan D, Innes A, Kosteniuk J. Dementia care in rural and remote settings: a systematic review of formal or paid care. Maturitas. Jan 2011;68(1):17-33. [doi: 10.1016/j.maturitas.2010.09.008] [Medline: 21041045]
21. Barth J, Nickel F, Kolominsky-Rabas PL. Diagnosis of cognitive decline and dementia in rural areas - a scoping review. Int J Geriatr Psychiatry. Mar 2018;33(3):459-474. [doi: 10.1002/gps.4841] [Medline: 29314221]
22. Watt JA, Lane NE, Veroniki AA, et al. Diagnostic accuracy of virtual cognitive assessment and testing: systematic review and meta-analysis. J Am Geriatr Soc. Jun 2021;69(6):1429-1440. [doi: 10.1111/jgs.17190] [Medline: 33948937]
23. Costanzo MC, Arcidiacono C, Rodolico A, Panebianco M, Aguglia E, Signorelli MS. Diagnostic and interventional implications of telemedicine in Alzheimer’s disease and mild cognitive impairment: a literature review. Int J Geriatr Psychiatry. Jan 2020;35(1):12-28. [doi: 10.1002/gps.5219] [Medline: 31617247]
24. Beishon LC, Elliott E, Hietamies TM, et al. Diagnostic test accuracy of remote, multidomain cognitive assessment (telephone and video call) for dementia. Cochrane Database Syst Rev. Apr 8, 2022;4(4):CD013724. [doi: 10.1002/14651858.CD013724.pub2] [Medline: 35395108]
25. McCleery J, Laverty J, Quinn TJ. Diagnostic test accuracy of telehealth assessment for dementia and mild cognitive impairment. Cochrane Database Syst Rev. Jul 20, 2021;7(7):CD013786. [doi: 10.1002/14651858.CD013786.pub2] [Medline: 34282852]
26. Sohn M, Yang J, Sohn J, Lee JH. Digital healthcare for dementia and cognitive impairment: a scoping review. Int J Nurs Stud. Apr 2023;140:104413. [doi: 10.1016/j.ijnurstu.2022.104413] [Medline: 36821951]
27. Rai HK, Kernaghan D, Schoonmade L, Egan KJ, Pot AM. Digital technologies to prevent social isolation and loneliness in dementia: a systematic review. J Alzheimers Dis. 2022;90(2):513-528. [doi: 10.3233/JAD-220438] [Medline: 36120780]
28. Scerbe A, O’Connell ME, Astell A, et al. Digital tools for delivery of dementia education for caregivers of persons with dementia: a systematic review and meta-analysis of impact on caregiver distress and depressive symptoms. PLoS One. 2023;18(5):e0283600. [doi: 10.1371/journal.pone.0283600] [Medline: 37196022]
29. Binng D, Splonskowski M, Jacova C. Distance assessment for detecting cognitive impairment in older adults: a systematic review of psychometric evidence. Dement Geriatr Cogn Disord. 2020;49(5):456-470. [doi: 10.1159/000511945] [Medline: 33291097]
30. Naslund JA, Mitchell LM, Joshi U, Nagda D, Lu C. Economic evaluation and costs of telepsychiatry programmes: a systematic review. J Telemed Telecare. Jun 2022;28(5):311-330. [doi: 10.1177/1357633X20938919] [Medline: 32746762]
31. Zhao Q, Li C, Zhang Y, et al. Economic evaluations of electronic health interventions for people with age-related cognitive impairment and their caregivers: a systematic review. Int J Geriatr Psychiatry. Sep 2023;38(9):e5990. [doi: 10.1002/gps.5990] [Medline: 37655517]
32. Brims L, Oliver K. Effectiveness of assistive technology in improving the safety of people with dementia: a systematic review and meta-analysis. Aging Ment Health. Aug 3, 2019;23(8):942-951. [doi: 10.1080/13607863.2018.1455805]
33. Eaglestone G, Gkaintatzi E, Stoner C, Pacella R, McCrone P. Effectiveness of community non-pharmacological interventions for mild cognitive impairment and dementia: a systematic review of economic evaluations and a review of reviews. medRxiv. Preprint posted online on 2022. [doi: 10.1101/2022.12.16.22283561]
34. Vandepitte S, Van Den Noortgate N, Putman K, Verhaeghe S, Faes K, Annemans L. Effectiveness of supporting informal caregivers of people with dementia: a systematic review of randomized and non-randomized controlled trials. J Alzheimers Dis. Apr 8, 2016;52(3):929-965. [doi: 10.3233/JAD-151011] [Medline: 27079704]
35. Saragih ID, Tonapa SI, Porta CM, Lee B. Effects of telehealth intervention for people with dementia and their carers: a systematic review and meta‐analysis of randomized controlled studies. J Nurs Scholarsh. Nov 2022;54(6):704-719. URL: <https://sigmapubs.onlinelibrary.wiley.com/toc/15475069/54/6> [doi: 10.1111/jnu.12797]
36. Lins S, Hayder-Beichel D, Rücker G, et al. Efficacy and experiences of telephone counselling for informal carers of people with dementia. Cochrane Database Syst Rev. Sep 1, 2014;2014(9):CD009126. [doi: 10.1002/14651858.CD009126.pub2] [Medline: 25177838]
37. Durepos P, MacLean R, Ricketts N, et al. Engaging care partners of persons living with dementia in acceptance and commitment therapy (ACT) programs: a scoping review. Aging Ment Health. May 2024;28(5):725-737. [doi: 10.1080/13607863.2023.2288864] [Medline: 38100551]
38. Muirhead K, Macaden L, Smyth K, et al. Establishing the effectiveness of technology-enabled dementia education for health and social care practitioners: a systematic review. Syst Rev. Sep 21, 2021;10(1):252. [doi: 10.1186/s13643-021-01781-8] [Medline: 34548101]
39. Kruse CS, Fohn J, Umunnakwe G, Patel K, Patel S. Evaluating the facilitators, barriers, and medical outcomes commensurate with the use of assistive technology to support people with dementia: a systematic review literature. Healthcare (Basel). Aug 18, 2020;8(3):278. [doi: 10.3390/healthcare8030278] [Medline: 32824711]
40. Caprioli T, Mason S, Tetlow H, Reilly S, Giebel C. Exploring the views and the use of information and communication technologies to access post-diagnostic support by people living with dementia and unpaid carers: a systematic review. Aging Ment Health. Dec 2, 2023;27(12):2329-2345. [doi: 10.1080/13607863.2023.2196246]
41. Hung L, Wong J, Smith C, et al. Facilitators and barriers to using telepresence robots in aged care settings: a scoping review. J Rehabil Assist Technol Eng. 2022;9:20556683211072385. [doi: 10.1177/20556683211072385] [Medline: 35083063]
42. Gentry MT, Lapid MI, Rummans TA. Geriatric telepsychiatry: systematic review and policy considerations. Am J Geriatr Psychiatry. Feb 2019;27(2):109-127. [doi: 10.1016/j.jagp.2018.10.009] [Medline: 30416025]
43. Pinto-Bruno ÁC, García-Casal JA, Csipke E, Jenaro-Río C, Franco-Martín M. ICT-based applications to improve social health and social participation in older adults with dementia. A systematic literature review. Aging Ment Health. Jan 2017;21(1):58-65. [doi: 10.1080/13607863.2016.1262818] [Medline: 27936876]
44. Zhu EM, Buljac-Samardžić M, Ahaus K, Sevdalis N, Huijsman R. Implementation and dissemination of home- and community-based interventions for informal caregivers of people living with dementia: a systematic scoping review. Implement Sci. Nov 8, 2023;18(1):60. [doi: 10.1186/s13012-023-01314-y] [Medline: 37940960]
45. Coumoundouros C, Mårtensson E, Ferraris G, et al. Implementation of e-mental health interventions for informal caregivers of adults with chronic diseases: mixed methods systematic review with a qualitative comparative analysis and thematic synthesis. JMIR Ment Health. Nov 30, 2022;9(11):e41891. [doi: 10.2196/41891] [Medline: 36314782]
46. Gately ME, Trudeau SA, Moo LR. In-home video telehealth for dementia management: implications for rehabilitation. Curr Geriatr Rep. Sep 1, 2019;8(3):239-249. [doi: 10.1007/s13670-019-00297-3] [Medline: 32015957]
47. D’Onofrio G, Sancarlo D, Ricciardi F, et al. Information and communication technologies for the activities of daily living in older patients with dementia: a systematic review. J Alzheimers Dis. 2017;57(3):927-935. [doi: 10.3233/JAD-161145] [Medline: 28304297]
48. Pit SW, Horstmanshof L, Moehead A, Hayes O, Schache V, Parkinson L. International standards for dementia workforce education and training: a scoping review. Gerontologist. Feb 1, 2024;64(2):gnad023. [doi: 10.1093/geront/gnad023] [Medline: 37071967]
49. Spencer L, Potterton R, Allen K, Musiat P, Schmidt U. Internet-based interventions for carers of individuals with psychiatric disorders, neurological disorders, or brain injuries: systematic review. J Med Internet Res. Jul 9, 2019;21(7):e10876. [doi: 10.2196/10876] [Medline: 31290399]
50. Leng M, Zhao Y, Xiao H, Li C, Wang Z. Internet-based supportive interventions for family caregivers of people with dementia: systematic review and meta-analysis. J Med Internet Res. Sep 9, 2020;22(9):e19468. [doi: 10.2196/19468] [Medline: 32902388]
51. Gonella S, Mitchell G, Bavelaar L, et al. Interventions to support family caregivers of people with advanced dementia at the end of life in nursing homes: a mixed-methods systematic review. Palliat Med. Feb 2022;36(2):268-291. [doi: 10.1177/02692163211066733] [Medline: 34965759]
52. Müller C, Lautenschläger S, Meyer G, Stephan A. Interventions to support people with dementia and their caregivers during the transition from home care to nursing home care: a systematic review. Int J Nurs Stud. Jun 2017;71:139-152. [doi: 10.1016/j.ijnurstu.2017.03.013] [Medline: 28411508]
53. Kruse CS, Mileski ME, Wilkinson R, Hock B, Samson R, Castillo T. Leveraging technology to diagnose Alzheimer’s disease: a systematic review and meta-analysis. Healthcare (Basel). Nov 21, 2023;11(23):3013. [doi: 10.3390/healthcare11233013] [Medline: 38063581]
54. Brito S de, Scianni AA, Peniche P da C, Faria C de M. Measurement properties of outcome measures used in neurological telerehabilitation: a systematic review using COSMIN checklist. Clin Rehabil. Mar 2023;37(3):415-435. [doi: 10.1177/02692155221129834] [Medline: 36448251]
55. El-Saifi N, Moyle W, Jones C, Tuffaha H. Medication adherence in older patients with dementia: a systematic literature review. J Pharm Pract. Jun 2018;31(3):322-334. [doi: 10.1177/0897190017710524] [Medline: 28539102]
56. Bacanoiu MV, Danoiu M. New strategies to improve the quality of life for normal aging versus pathological aging. J Clin Med. Jul 20, 2022;11(14):4207. [doi: 10.3390/jcm11144207] [Medline: 35887969]
57. Lee DCA, Tirlea L, Haines TP. Non-pharmacological interventions to prevent hospital or nursing home admissions among community-dwelling older people with dementia: a systematic review and meta-analysis. Health Soc Care Community. Sep 2020;28(5):1408-1429. [doi: 10.1111/hsc.12984] [Medline: 32223022]
58. Amiri P, Niazkhani Z, Pirnejad H, ShojaeiBaghini M, Bahaadinbeigy K. Objectives, outcomes, facilitators, and barriers of telemedicine systems for patients with Alzheimer’s disease and their caregivers and care providers: a systematic review. Arch Iran Med. Aug 1, 2022;25(8):564-573. [doi: 10.34172/aim.2022.90] [Medline: 37543880]
59. Nissen RM, Serwe KM. Occupational therapy telehealth applications for the dementia-caregiver dyad: a scoping review. Phys Occup Ther Geriatr. Oct 2, 2018;36(4):366-379. [doi: 10.1080/02703181.2018.1536095]
60. Etxeberria I, Salaberria K, Gorostiaga A. Online support for family caregivers of people with dementia: a systematic review and meta-analysis of RCTs and quasi-experimental studies. Aging Ment Health. Jul 3, 2021;25(7):1165-1180. [doi: 10.1080/13607863.2020.1758900]
61. Egan KJ, Pinto-Bruno ÁC, Bighelli I, et al. Online training and support programs designed to improve mental health and reduce burden among caregivers of people with dementia: a systematic review. J Am Med Dir Assoc. Mar 2018;19(3):200-206. [doi: 10.1016/j.jamda.2017.10.023] [Medline: 29306605]
62. Elliot V, Morgan D, Kosteniuk J, et al. Palliative and end-of-life care for people living with dementia in rural areas: a scoping review. PLoS One. 2021;16(1):e0244976. [doi: 10.1371/journal.pone.0244976] [Medline: 33444351]
63. Boyle LD, Husebo BS, Vislapuu M. Promotors and barriers to the implementation and adoption of assistive technology and telecare for people with dementia and their caregivers: a systematic review of the literature. BMC Health Serv Res. Dec 23, 2022;22(1):1573. [doi: 10.1186/s12913-022-08968-2] [Medline: 36550456]
64. Elvish R, Lever SJ, Johnstone J, Cawley R, Keady J. Psychological interventions for carers of people with dementia: a systematic review of quantitative and qualitative evidence. Couns and Psychother Res. Jun 2013;13(2):106-125. [doi: 10.1080/14733145.2012.739632]
65. Hunter MB, Jenkins N, Dolan C, Pullen H, Ritchie C, Muniz-Terrera G. Reliability of telephone and videoconference methods of cognitive assessment in older adults with and without dementia. J Alzheimers Dis. 2021;81(4):1625-1647. [doi: 10.3233/JAD-210088] [Medline: 33967052]
66. González-Fraile E, Ballesteros J, Rueda JR, Santos-Zorrozúa B, Solà I, McCleery J. Remotely delivered information, training and support for informal caregivers of people with dementia. Cochrane Database Syst Rev. Jan 4, 2021;1(1):CD006440. [doi: 10.1002/14651858.CD006440.pub3] [Medline: 33417236]
67. Lin JS, O’Connor E, Rossom RC, Perdue LA, Eckstrom E. Screening for cognitive impairment in older adults: a systematic review for the U.S. Preventive Services Task Force. Ann Intern Med. Nov 5, 2013;159(9):601-612. [doi: 10.7326/0003-4819-159-9-201311050-00730] [Medline: 24145578]
68. Corbett A, Stevens J, Aarsland D, et al. Systematic review of services providing information and/or advice to people with dementia and/or their caregivers. Int J Geriat Psychiatry. Jun 2012;27(6):628-636. [doi: 10.1002/gps.2762]
69. Maresova P, Tomsone S, Lameski P, et al. Technological solutions for older people with Alzheimer’s disease: review. Curr Alzheimer Res. Aug 15, 2018;15(10):975-983. [doi: 10.2174/1567205015666180427124547]
70. Bauernschmidt D, Hirt J, Langer G, et al. Technology-based counselling for people with dementia and their informal carers: a systematic review and meta-analysis. J Alzheimers Dis. 2023;93(3):891-906. [doi: 10.3233/JAD-221194] [Medline: 37125549]
71. Mao W, Qi X, Chi I, Wichinsky L, Wu B. Technology-based interventions to address social isolation and loneliness among informal dementia caregivers: a scoping review. J Am Med Dir Assoc. Nov 2023;24(11):1700-1707. [doi: 10.1016/j.jamda.2023.08.005] [Medline: 37678415]
72. Zhu A, Cao W, Zhou Y, Xie A, Cheng Y, Chu SF. Tele-health intervention for carers of dementia patients-a systematic review and meta-analysis of randomized controlled trials. Front Aging Neurosci. 2021;13:612404. [doi: 10.3389/fnagi.2021.612404] [Medline: 33643022]
73. Carotenuto A, Traini E, Fasanaro AM, Battineni G, Amenta F. Tele-neuropsychological assessment of Alzheimer’s disease. J Pers Med. Jul 21, 2021;11(8):688. [doi: 10.3390/jpm11080688] [Medline: 34442332]
74. Ferreira Santana R, Vaqueiro Dantas R, et al. Telecare to elderly people with Alzheimer and their caregivers: systematic review. Cienc Cuid Saude. 2018;17:1-6. [doi: 10.4025/cienccuidsaude.v17i4.41653]
75. Söylemez BA, Özgül E, Küçükgüçlü Ö, Yener G. Telehealth applications used for self-efficacy levels of family caregivers for individuals with dementia: a systematic review and Meta-analysis. Geriatr Nurs (Lond). Jan 2023;49:178-192. [doi: 10.1016/j.gerinurse.2022.12.001]
76. Washington SE, Bollinger RM, Edwards E, McGowan L, Stephens S. Telehealth delivery of evidence-based intervention within older adult populations: a scoping review. OTJR (Thorofare N J). Jul 2023;43(3):467-477. [doi: 10.1177/15394492231180838] [Medline: 37322873]
77. Graven LJ, Glueckauf RL, Regal RA, Merbitz NK, Lustria MLA, James BA. Telehealth interventions for family caregivers of persons with chronic health conditions: a systematic review of randomized controlled trials. Int J Telemed Appl. 2021;2021:3518050. [doi: 10.1155/2021/3518050] [Medline: 34093704]
78. Yi JS, Pittman CA, Price CL, Nieman CL, Oh ES. Telemedicine and dementia care: a systematic review of barriers and facilitators. J Am Med Dir Assoc. Jul 2021;22(7):1396-1402. [doi: 10.1016/j.jamda.2021.03.015] [Medline: 33887231]
79. Sekhon H, Sekhon K, Launay C, et al. Telemedicine and the rural dementia population: a systematic review. Maturitas. Jan 2021;143:105-114. [doi: 10.1016/j.maturitas.2020.09.001] [Medline: 33308615]
80. León-Salas B, González-Hernández Y, Infante-Ventura D, et al. Telemedicine for neurological diseases: a systematic review and meta-analysis. Eur J Neurol. Jan 2023;30(1):241-254. [doi: 10.1111/ene.15599] [Medline: 36256522]
81. Folder N, Power E, Rietdijk R, Christensen I, Togher L, Parker D. The effectiveness and characteristics of communication partner training programs for families of people with dementia: a systematic review. Gerontologist. Apr 1, 2024;64(4):gnad095. [doi: 10.1093/geront/gnad095] [Medline: 37439771]
82. Yu Y, Xiao L, Ullah S, et al. The effectiveness of internet-based psychoeducation programs for caregivers of people living with dementia: a systematic review and meta-analysis. Aging Ment Health. 2023;27(10):1895-1911. [doi: 10.1080/13607863.2023.2190082] [Medline: 36951611]
83. Hailey D, Roine R, Ohinmaa A. The effectiveness of telemental health applications: a review. Can J Psychiatry. Nov 2008;53(11):769-778. [doi: 10.1177/070674370805301109] [Medline: 19087471]
84. Lucero RJ, Fehlberg EA, Patel AGM, et al. The effects of information and communication technologies on informal caregivers of persons living with dementia: a systematic review. Alzheimers Dement (N Y). 2018;5:1-12. [doi: 10.1016/j.trci.2018.11.003] [Medline: 30623020]
85. Rueda Daz LJ, Monteiro da Cruz DL. The efficacy of telephone use to assist and improve the wellbeing of family caregivers of persons with chronic diseases: a systematic review. JBI Database System Rev Implement Rep. Dec 2014;12(12):106-140. [doi: 10.11124/jbisrir-2014-1566]
86. Nkodo JA, Gana W, Debacq C, et al. The role of telemedicine in the management of the behavioral and psychological symptoms of dementia: a systematic review. Am J Geriatr Psychiatry. Oct 2022;30(10):1135-1150. [doi: 10.1016/j.jagp.2022.01.013] [Medline: 35241355]
87. Liang J, Aranda MP. The use of telehealth among people living with dementia-caregiver dyads during the COVID-19 pandemic: scoping review. J Med Internet Res. May 25, 2023;25:e45045. [doi: 10.2196/45045] [Medline: 37227755]
88. Armstrong MJ, Alliance S. Virtual support groups for informal caregivers of individuals with dementia: a scoping review. Alzheimer Dis Assoc Disord. 2019;33(4):362-369. [doi: 10.1097/WAD.0000000000000349] [Medline: 31567144]
89. Wood M, Walshe C, McCullagh A. What are the digitally enabled psychosocial interventions delivered by trained practitioners being offered to adults with life-shortening illnesses and palliative care needs and their informal and professional caregivers? A scoping review. Palliat Support Care. Aug 2023;21(4):727-740. [doi: 10.1017/S1478951523000172] [Medline: 36994819]
90. Sun Y, Ji M, Leng M, Wang Z. Which cognitive behavioral therapy delivery formats work for depressive symptoms in dementia caregivers? - A systematic review and network meta-analysis of randomized controlled trials. J Affect Disord. Jul 1, 2022;308:181-187. [doi: 10.1016/j.jad.2022.04.055] [Medline: 35429541]
91. Kishita N, Hammond L, Dietrich CM, Mioshi E. Which interventions work for dementia family carers?: an updated systematic review of randomized controlled trials of carer interventions. Int Psychogeriatr. Nov 2018;30(11):1679-1696. [doi: 10.1017/S1041610218000947] [Medline: 30017008]
