# Supplementary material for: An Overview of Reviews on Telemedicine and Telehealth in Dementia Care: Mixed Methods Synthesis
Source: JMIR Ment Health. 2025 Nov 6;12:e75266. doi: 10.2196/75266 (PMC12975415; doi:10.2196/75266)
Supplement: Multimedia Appendix 2 [file mental-v12-e75266-s002.docx]

| **Table S1.** Primary information on included systematic and scoping reviews (*n = 91*) | | | | |
| --- | --- | --- | --- | --- |
| **Author ID** | **Year** | **Journal** | **Objective** | **Population** |
| Di Lorito C. | 2022 | International Journal of Geriatric Psychiatry | a) To describe types, design, content, and delivery features of digital health interventions for PLWD or MCI. b) To summarize potential physical, cognitive, behavioral and psychological effects of the intervention and identify interventions with greater effect sizes. | Older adults living with dementia or MCI |
| Daly Lynn J. | 2019 | Dementia (London, England) | a) To review research on the use of electronic assistive technology within long-term residential care settings. | PLWD and their caregivers |
| Dam A.E.H. | 2016 | Maturitas | a) To review effect of social support interventions on caregiver support and well-being variables b) To evaluate methodological quality of research in the field | Informal caregivers of community-dwelling PLWD |
| Elbaz S. | 2021 | Frontiers In Neurology | a) To review the use, feasibility and acceptability of telemedicine for o older people living with dementia during the COVID-19 pandemic b) To explore mental health impacts of these technologies | Older people living with dementia during COVID-19 |
| Jackson D. | 2016 | Archives of Gerontology And Geriatrics | a) To review effectiveness of telephone, internet-based or combined interventions in social support and mental health needs of family caregivers of PLWD. b) To assess impact of these interventions on the management of clinical risk factors by caregivers and if interventions addressed specific dementia etiologies | Unpaid, informal carers of PLWD or MCI |
| Martin-Khan M. | 2010 | Journal of Telemedicine And Telecare | a) To review reliability of standardized assessment tools for cognitive impairment administered by telephone | Older adults undergoing cognitive impairment screening |
| Riley C.O. | 2022 | Jrsm Open | a) To review validity of telephone cognitive screening tools for the identification of MCI and dementia compared to in-person assessments. | People undergoing cognitive screening for MCI and dementia |
| Elliott E. | 2020 | Current Alzheimer Research | a) To summarize teste accuracy of telephone-based cognitive screening assessments for identification of dementia or MCI | PLWD or MCI |
| van der Wardt V. | 2017 | Preventive Medicine Reports | a) To review effectiveness of strategies implemented in exercise studies to support adherence among PLWD or MCI | PLWD or MCI |
| Martínez-Alcalá | 2015 | Iber. Conf. Inf. Syst. Technol., CISTI | a) To integrate the knowledge of older adults living with AD and the opportunities that ICTs provide to individuals, their families and health services. | People living with AD |
| Gagnon-Roy M. | 2017 | The American Journal of Occupational Therapy | a) To review types and characteristics of assistive technologies that might address safety issues and improve participation of PLWD in meaningful occupations b) To evaluate advantages and disadvantages of using assistive technologies in this context c) To determine key factors for selection of assistive technologies considering application in the population above | Older adults living with dementia |
| Kwan R.Y.-C. | 2013 | International Journal of Environmental Research And Public Health | a) To review telephone-based cognitive assessments validity (comparison with medical assessment, comparison with application of the same instrument face-to-face, and comparison with validated instruments) b) To discuss its potential enhancements by smartphone technology. | People undergoing cognitive assessment (healthy and PLWD or MCI) |
| Lin X. | 2023 | Australasian Journal On Ageing | a) To inform the identification and development of carer-reported measures for a dementia clinical quality registry | Informal carers of PLWD in the community |
| Cotelli M. | 2019 | Journal Of Telemedicine And Telecare | a) To review efficacy of cognitive telerehabilitation interventions in individuals with MCI, AD or FTD compared with conventional FTF rehabilitation. | People living with MCI, AD or FTD |
| Dedzoe, Justice Dey-Seshie | 2023 | Healthcare (Basel, Switzerland) | a) To summarize research on reflective collaborative learning through virtual communities of practice (vCoP) b) To review research on facilitators and barriers associated with resilience capacity and knowledge acquisition through vCoP. | People living with dementia (PLWD), their informal and formal caregivers. |
| Waller A. | 2017 | BMC Geriatrics | a) To review research on acceptability, utilization and effectiveness of telehealth interventions for caregivers of PLWD considering scope, volume and quality. | Caregivers of PLWD |
| Maggio M.G. | 2023 | Frontiers in Neurology | a) To describe the available software and device used for cognitive training or rehabilitation interventions for people living with neurological conditions | People living with neurological conditions |
| Piau A. | 2019 | Journal of Medical Internet Research | a) To review real-life, home-based use of technologies for early detection and follow-up of PLWD or MCI b) To describe potential implications in clinical practice | Cognitively healthy older adults and living with cognitive decline (from subjective cognitive complaints to early AD) |
| Gaigher J.M. | 2022 | Frontiers in Psychiatry | a) To review and discuss potential impacts of the COVID-19 pandemic on PLWD and t their caregivers’ mental health. | Older adults living with dementia and their caregivers |
| Morgan D. | 2011 | Maturitas | a) To review formal caregiving for PLWD in rural and remote areas, considering the continuum of early detection, diagnosis and long-term care | PLWD and in rural and remote areas and their carers |
| Barth J. | 2018 | International Journal of Geriatric Psychiatry | a) To review interventions applied to older people in rural areas for screening and diagnosis of cognitive impairment and dementia | Older people living in rural areas undergoing screening or diagnostic assessment for cognitive impairment and dementia |
| Watt J.A. | 2021 | Journal of The American Geriatrics Society | a) To summarize diagnostic accuracy of virtual cognitive assessments b) To analyze potential barriers for implementation | People undergoing cognitive assessment |
| Costanzo M.C. | 2020 | International Journal of Geriatric Psychiatry | a) To summarize the implementation of telemedicine for diagnosis, treatment, and follow‐ up of individuals with AD or MCI and their caregivers. | People living with MCI or AD, excluding other dementia etiologies |
| Beishon L.C. | 2022 | Cochrane Database of Systematic Reviews | a) To evaluate accuracy of remotely delivered multidomain cognitive tests for diagnosis of dementia b) To analyze potential differences in the scores for remote tests and compare to face-to-face delivery. | Adults undergoing cognitive testing |
| McCleery J. | 2020 | Cochrane Database of Systematic Reviews | a) To evaluate diagnostic test accuracy of telehealth assessment for dementia and MCI | People being assessed for the diagnosis of dementia and mild cognitive impairment |
| Sohn M. | 2023 | International Journal of Nursing Studies | a) To identify studies on digital technology used for dementia and mild cognitive impairment b) To explore how measures used to confirm appropriateness or effectiveness of each technology are constructed and if they fit their purpose | PLWD or cognitive impairment and their caregivers |
| Rai H.K. | 2022 | Journal of Alzheimer's Disease | a) To review digital technologies impact on social isolation and loneliness prevention in PLWD | PLWD |
| Scerbe A. | 2023 | Plos ONE | a) To review technologies used for dementia education with a focus on the quality of evidence and mental health impact for caregivers of PLWD | Caregivers of PLWD |
| Binng, Davina | 2020 | Dementia and Geriatric Cognitive Disorders | a) To review psychometric properties of remote cognitive impairment assessment of older adults | Older adults undergoing assessment for cognitive impairment |
| Naslund J.A. | 2022 | Journal of Telemedicine And Telecare | a) To review economic evaluations and costs of developing and delivering telepsychiatry programmes for mental healthcare | People accessing telepsychiatry services for clinical care of mental disorders |
| Zhao Q. | 2023 | International Journal of Geriatric Psychiatry | a) To evaluate economic evidence of e-health interventions applied to people with age-related cognitive impairment and their caregivers | People with age-related cognitive impairment and their caregivers |
| Brims L. | 2019 | Aging & Mental Health | a) To review the effectiveness of AT in improving safety of PLWD in the domestic setting | PLWD and in their homes |
| Eaglestone G. | 2022 | Medrxiv | a) To review economic evaluations of nursing home and community non-pharmacological interventions for PLWD or MCI |  |
| Vandepitte, Sophie | 2016 | Journal of Alzheimer's Disease | a) To review effectiveness of supportive strategies applied to informal caregivers of PLWD | PLWD and their informal caregivers |
| Saragih I.D. | 2022 | Journal of Nursing Scholarship | a) To review effectiveness of telehealth psychoeducational and behavioral interventions applied to PLWD and their caregivers | PLWD and their caregivers |
| Lins S. | 2014 | Cochrane Database of Systematic Reviews | a) To review efficacy of telephone counselling for informal carers of PLWD b) To summarize carers’ and counsellors’ experiences in receiving and conducting telephone counselling c) To identify which parts of the intervention are valued and have a positive effect as well as those that requires improvement | Informal caregivers of PLWD who provide unpaid care |
| Durepos P. | 2023 | Aging & Mental Health | a) To review literature on ACT programs delivered to care partners of PLWD b) To describe influential engagement factors of ACT programs | Care partners of PLWD |
| Muirhead K. | 2021 | Systematic Reviews | a) To assess the impact of educational interventions on dementia care and associated healthcare professionals' knowledge, skills, and attitudes. | Healthcare professionals supporting PLWD |
| Kruse, Clemens Scott | 2020 | Healthcare (Basel, Switzerland) | a) To review barriers, facilitators and medical outcomes of assistive technologies for PLWD and their caregivers | PLWD and their carers |
| Caprioli T. | 2023 | Aging & Mental Health | a) To review how information communication technologies (iCT) have been used to access remote post-diagnostic support that address the needs of PLWD and unpaid carers b) To explore care recipients’ views on accessing support remotely. | PLWD in the community (including supportive / assistive living accommodation) and their unpaid caregivers |
| Hung, Lillian | 2022 | Journal of Rehabilitation and Assistive Technologies Engineering | a) To review barriers and facilitators to the use of telepresence robots in aged care settings | Older people in aged care settings |
| Gentry M.T. | 2019 | American Journal of Geriatric Psychiatry | a) To review feasibility, acceptability, and cost-effectiveness of telemedicine use for psychiatric assessment and treatment | Older people undergoing psychiatric assessment and treatment |
| Pinto-Bruno AC. | 2017 | Aging & Mental Health | a) To evaluate utility of ICT to promote ‘social health’ and ‘active ageing’ in PLWD | PLWD |
| Zhu E.M. | 2023 | Implementation Science : IS | a) To review implementation strategies of home delivered and community-based interventions for informal caregivers of PLWD b) To evaluate outcomes, barriers and facilitators related to these interventions | Informal caregivers of PLWD |
| Coumoundouros C. | 2022 | JMIR Mental Health | a) To examine factors associated with the effectiveness and implementation of e–mental health interventions for informal caregivers of individuals living with chronic disease. | Informal adult caregivers providing unpaid care to people with cancer, chronic obstructive pulmonary disease, dementia, diabetes, heart disease, or stroke. |
| Gately ME | 2019 | Current Geriatrics Reports | a) To review use of in-home video telehealth for taking care of PLWD and their caregivers, focusing on the implications for rehabilitation professionals. | PLWD and their caregivers |
| D'Onofrio G. | 2017 | Journal of Alzheimer's Disease | a) To review ICT concepts and approaches in supporting activities of daily living for older people living with dementia b) To describe potential effects of the intervention on self-reliance | Older people living with dementia |
| Pit S.W. | 2024 | The Gerontologist | a) To review elements of national or state-wide standards on dementia education with potential of development of international standards for dementia workforce training and education | Health care professionals, PLWD, informal carers and the general community |
| Spencer L. | 2019 | Journal of Medical Internet Research | a) To review potential impact of internet-based interventions on the mental health of informal caregivers of people living with psychiatric or neurological conditions b) To appraise research quality  c) To compare internet-based interventions with other type of interventions such as face-to-face | Informal caregivers of people living with psychiatric or neurological conditions |
| Leng M. | 2020 | Journal of Medical Internet Research | a) To summarize efficacy of internet-based supportive intervention on health outcomes of family caregivers of PLWD b) To evaluate effects of the intervention on the care recipients | PLWD and their family caregivers |
| Gonella S. | 2022 | Palliative Medicine | a) To summarize interventions to support family caregivers of people living with advanced dementia at the end of life in nursing homes  b) To provide clinical practice recommendations | Family caregivers of people living with advanced dementia |
| Muller C. | 2017 | International Journal of Nursing Studies | a) To evaluate interventions supporting informal caregivers of PLWD during transition from home care to nursing home care | Informal caregivers of PLWD |
| Kruse, Clemens Scott | 2023 | Healthcare (Basel, Switzerland) | a) To summarize effectiveness of technology-based diagnosis of AD  b) To use meta-analysis data aiming acceleration of the construction of clinical guidelines. | People undergoing diagnosis for AD |
| Brito S.A.F. | 2023 | Clinical Rehabilitation | a) To summarize the measurement properties (reliability, validity, and responsiveness) and clinical utility of measurement tools used in telerehabilitation in individuals with neurological conditions. | Adults living with any neurological condition |
| El-Saifi N. | 2018 | Journal of Pharmacy Practice | a) To review medication adherence of older people living with dementia including contributing factors and available interventions. | Older people living with dementia or cognitive impairment |
| Bacanoiu 2022 | 2022 | Journal of Clinical Medicine | To evaluate the intervention patterns, surveys delivered through variables online platforms and tools to reflect the stagnation of early aging and the evolution of patients living with PD and dementia. | People living with motor and cognitive impairments from Parkinson’s disease (PD) and AD |
| Lee D.-C.A. | 2020 | Health & Social Care in The Community | a) To review and summarize potential impact of non-pharmacological interventions in the prevention of hospital or nursing home admissions among older PLWD | Community-dwelling older PLWD and their informal caregivers |
| Amiri 2022 | 2022 | Archives of Iranian Medicine | a) To review outcomes and objectives of application of telemedicine for people living with AD and their caregivers b) To asses barriers and facilitators related to implementation of telemedicine systems for the population above | People living with AD their caregivers and healthcare providers |
| Nissen, R.M. | 2018 | Phys. Occup. Ther. Geriatr. | a) To review application of telehealth by occupational therapy practitioners applied to PLWD and their caregivers. | Dementia-caregiver dyad |
| Etxeberria I. | 2021 | Aging & Mental Health | a) To summarize efficacy of online support programs on psychological well-being of caregivers of PLWD | Family caregivers of PLWD at home |
| Egan K.J. | 2018 | Journal of The American Medical Directors Association | a) To identify studies of Internet-based interventions designed to train and support caregivers of PLWD | Caregivers of PLWD |
| Elliot V. | 2021 | Plos ONE | a) To review palliative and end-of life care in rural areas for PLWD and their families b) To describe key findings, literature gaps and perform recommendations for future research | PLWD in rural areas and their families |
| Boyle L.D. | 2022 | BMC Health Services Research | a) To review drivers and barriers to the implementation and adoption of assistive technologies for PLWD and their informal and formal caregivers | PLWD and their formal or informal caregivers |
| Elvish, R. | 2013 | Couns. Psychother. Res. | a) To review quantitative and qualitative research on psychological interventions for carers of PLWD | Caregivers of PLWD |
| Hunter M.B. | 2021 | Journal of Alzheimer's Disease | a) To review cross-modal test-retest reliability of cognitive tests comparing in-person versus remote administration in neurologic healthy individuals and those living with dementia. | People undergoing cognitive assessment (healthy and PLWD) |
| Gonzalez-Fraile E. | 2021 | Cochrane Database of Systematic Reviews | a) To evaluate acceptability and potential effect of remote information, training and support interventions on burden, mood and quality of life of informal caregivers of PLWD. | Informal caregivers of PLWD |
| Lin J.S. | 2013 | Annals Of Internal Medicine | a) To review test accuracy of cognitive screening instrument in older adults to inform the US Preventive Services Task Force b) To review benefits and harms of interventions to treat cognitive impairment | Older adults undergoing cognitive impairment screening |
| Corbett A. | 2012 | International Journal of Geriatric Psychiatry | a) To review whether information services confer significant benefit for quality of life, neuropsychiatric symptoms and carer burden. | PLWD and their caregivers |
| Maresova P. | 2018 | Current Alzheimer Research | a) To review assistive technologies for people living with AD | People living with AD |
| Bauernschmidt D. | 2023 | Journal of Alzheimer's Disease | a) To summarize effectiveness of technology-based counselling interventions for PLWD and their informal caregivers | PLWD and their informal caregivers |
| Mao W. | 2023 | Journal of The American Medical Directors Association | a) To Identify technology-based interventions targeting social isolation and loneliness among informal caregivers of PLWD b) To review characteristics and components of these technology-based interventions c) To inform and develop implications for future interventions, practice and policy | Informal caregivers of PLWD |
| Zhu A. | 2021 | Frontiers in Aging Neuroscience | a) To summarize effect of remotely delivered psychoeducation and psychotherapy interventions on mental health outcomes of caregivers of PLWD | Caregivers of PLWD |
| Carotenuto A. | 2021 | Journal of Personalized Medicine | a) To explore if the neuropsychological tests traditionally employed in face-to-face (FTF) contexts are reliable via telemedicine. | PLWD or cognitive impairment |
| Ferreira Santana, Rosimere | 2018 | Ciencia, Cuidado e Saude | a) To review use of nursing telecare intervention for older people living with AD and their caregivers | People living with AD and their caregivers |
| SÃ¶ylemez BA | 2022 | Geriatric Nursing (New York, N.Y.) | a) To review the effect of telehealth applications for family caregivers of PLWD considering self-efficacy levels, caregiving burden, stress, depression, and quality of life. | Family caregivers of PLWD |
| Washington S.E. | 2023 | OTJR: Occupational Therapy Journal of Research | a) To review evidence-based occupational therapy telehealth interventions applied to older adults | Older adults |
| Graven L.J. | 2021 | International Journal of Telemedicine And Applications | a) To evaluate components and outcomes of telehealth interventions for family caregivers of people living with chronic health conditions | Family caregivers of peole living with chronic health conditions |
| Yi J.S. | 2021 | Journal of The American Medical Directors Association | a) To review use of synchronous in-home or clinic video-based telemedicine visits applied to older adults living with AD or MCI  c) To identify barriers and facilitators related to the intervention | Older adults living with AD or MCI |
| Sekhon H. | 2021 | Maturitas | a) To review the potential impact of telemedicine on health outcomes in older people living with dementia in rural areas | Older people living with dementia in rural areas |
| Leon-Salas B. | 2023 | European Journal of Neurology | a) To review effectiveness and safety of telemedicine combined with usual care compared to usual care for management and follow-up of people living with neurological conditions | People living with neurological conditions |
| Folder N. | 2023 | The Gerontologist | a) To review characteristics and effectiveness of Communication Partner Training (CPT) programs applied to family members of PLWD | Family members of PLWD |
| Yu Y. | 2023 | Aging & Mental Health | a) To describe characteristics and summarize effectiveness of internet-based psychoeducation programs applied to caregivers of PLWD | Caregivers of PLWD |
| Hailey D. | 2008 | Canadian Journal of Psychiatry | a) To review potential benefits of telemental health (including different technologies) considering clinical and administrative outcomes | People living with mental health disorders or substance abuse |
| Lucero R.J. | 2019 | Alzheimer's and Dementia: Translational Research and Clinical Interventions | a) To review potential effects of ICT interventions on the health of informal caregivers of PLWD | Informal caregivers of PLWD |
| Rueda Diaz L.J. | 2014 | JBI Database of Systematic Reviews and Implementation Reports | a) To review efficacy of interventions delivered using the telephone in improving wellbeing of family caregivers of people living with chronic disease | Family caregivers of people living with chronic diseases regardless of the disease type, severity and duration of care. |
| Nkodo J.-A. | 2022 | American Journal of Geriatric Psychiatry | a) To review use of telemedicine for management and follow-up of PLWD and behavioral/psychological symptoms and their caregivers | PLWD and their caregivers |
| Liang J. | 2023 | Journal of Medical Internet Research | a) Describe implementation of telehealth services for PLWD and their caregivers during the COVID-19 pandemic b) To review effectiveness, user experience, and barriers related to the implementation of these telehealth services | Community-dwelling PLWD or cognitive impairment and their family caregivers |
| Armstrong 2019 | 2019 | Alzheimer Disease and Associated Disorders | a) To review approaches, outcomes, barriers, and facilitators for virtual support group development applied to informal caregivers of PLWD | Informal caregivers of PLWD |
| Wood M. | 2023 | Palliative & Supportive Care | a) To describe digitally enabled psychosocial interventions applied to adults living with shortening or terminal illnesses receiving palliative care and their caregivers b) To analyze delivery and evaluation of these interventions | People living with life-shortening illnesses and with palliative care needs and their informal and professional caregivers |
| Sun Y. | 2022 | Journal of Affective Disorders | a) To summarize effect of different delivery formats for Cognitive Behavioral Therapy (CBT) on depressive symptoms among caregivers of PLWD | Caregivers of PLWD |
| Kishita N. | 2018 | International Psychogeriatrics | a) To review and appraise efficacy of psychoeducation and psychotherapeutic (CBT) interventions on mental health outcomes of family carers of PLWD | Family carers of PLWD |
| Legend:  PLWD – People Living With Dementia  MCI – Mild Cognitive Impairment  AD – Alzheimer’s Disease  FTD – Frontotemporal Dementia  ICT – Information and Communication Technology  AT – Assistive Technology  ACT – Acceptance and Commitment Therapy  CBT – Cognitive Behavioral Therapy  CPT – Communication Partner Training  vCoP – Virtual Communities of Practice  FTF – Face-To-Face | | | | |

**Reference list of the 91 included studies**

Di Lorito C, Bosco A, Rai H, et al. A systematic literature review and meta-analysis on digital health interventions for people living with dementia and mild cognitive impairment. Int J Geriatr Psychiatry. Jun 2022;37(6). [doi: 10.1002/gps.5730] [Medline: 35588315]

Daly Lynn J, Rondón-Sulbarán J, Quinn E, Ryan A, McCormack B, Martin S. A systematic review of electronic assistive technology within supporting living environments for people with dementia. Dementia (London). 2019;18(7-8):2371-2435. [doi: 10.1177/1471301217733649] [Medline: 28990408]

Dam AEH, de Vugt ME, Klinkenberg IPM, Verhey FRJ, van Boxtel MPJ. A systematic review of social support interventions for caregivers of people with dementia: are they doing what they promise? Maturitas. Mar 2016;85:117-130. [doi: 10.1016/j.maturitas.2015.12.008] [Medline: 26857890]

Elbaz S, Cinalioglu K, Sekhon K, et al. A systematic review of telemedicine for older adults with dementia during COVID-19: an alternative to in-person health services? Front Neurol. 2021;12:761965. [doi: 10.3389/fneur.2021.761965] [Medline: 34970210]

Jackson D, Roberts G, Wu ML, Ford R, Doyle C. A systematic review of the effect of telephone, internet or combined support for carers of people living with Alzheimer’s, vascular or mixed dementia in the community. Arch Gerontol Geriatr. 2016;66:218-236. [doi: 10.1016/j.archger.2016.06.013] [Medline: 27372903]

Martin-Khan M, Wootton R, Gray L. A systematic review of the reliability of screening for cognitive impairment in older adults by use of standardised assessment tools administered via the telephone. J Telemed Telecare. 2010;16(8):422-428. [doi: 10.1258/jtt.2010.100209] [Medline: 21030488]

Riley CO, McKinstry B, Fairhurst K. Accuracy of telephone screening tools to identify dementia patients remotely: systematic review. JRSM Open. Sep 2022;13(9):20542704221115956. [doi: 10.1177/20542704221115956] [Medline: 36082188]

Elliott E, Green C, Llewellyn DJ, Quinn TJ. Accuracy of telephone-based cognitive screening tests: systematic review and meta-analysis. Curr Alzheimer Res. 2020;17(5):460-471. [doi: 10.2174/1567205017999200626201121] [Medline: 32589557]

van der Wardt V, Hancox J, Gondek D, et al. Adherence support strategies for exercise interventions in people with mild cognitive impairment and dementia: a systematic review. Prev Med Rep. Sep 2017;7:38-45. [doi: 10.1016/j.pmedr.2017.05.007] [Medline: 28593121]

Martinez-Alcala CI, Pliego-Pastrana P, Lopez-Noguerola JS, Rosales-Lagarde A, Zaleta-Arias ME. Adoption of ICT in the aging: systematic review based on ICT for Alzheimer’s disease and other senile dementias. Presented at: 2015 10th Iberian Conference on Information Systems and Technologies (CISTI); Jun 17-20, 2015; Aveiro, Portugal. [doi: 10.1109/CISTI.2015.7170393]

Gagnon-Roy M, Bourget A, Stocco S, Courchesne ACL, Kuhne N, Provencher V. Assistive technology addressing safety issues in dementia: a scoping review. Am J Occup Ther. 2017;71(5):7105190020p1-7105190020p10. [doi: 10.5014/ajot.2017.025817] [Medline: 28809655]

Kwan RYC, Lai CKY. Can smartphones enhance telephone-based cognitive assessment (TBCA)? Int J Environ Res Public Health. Dec 12, 2013;10(12):7110-7125. [doi: 10.3390/ijerph10127110] [Medline: 24351736]

Lin X, Ward SA, Pritchard E, et al. Carer-reported measures for a dementia registry: a systematic scoping review and a qualitative study. Australas J Ageing. Mar 2023;42(1):34-52. [doi: 10.1111/ajag.13148] [Medline: 36383194]

Cotelli M, Manenti R, Brambilla M, et al. Cognitive telerehabilitation in mild cognitive impairment, Alzheimer’s disease and frontotemporal dementia: a systematic review. J Telemed Telecare. Feb 2019;25(2):67-79. [doi: 10.1177/1357633X17740390] [Medline: 29117794]

Dedzoe JDS, Malmgren Fänge A, Christensen J, Lethin C. Collaborative learning through a virtual community of practice in dementia care support: a scoping review. Healthcare (Basel). Feb 26, 2023;11(5):692. [doi: 10.3390/healthcare11050692] [Medline: 36900696]

Waller A, Dilworth S, Mansfield E, Sanson-Fisher R. Computer and telephone delivered interventions to support caregivers of people with dementia: a systematic review of research output and quality. BMC Geriatr. Nov 16, 2017;17(1):265. [doi: 10.1186/s12877-017-0654-6] [Medline: 29145806]

Maggio MG, De Bartolo D, Calabrò RS, et al. Computer-assisted cognitive rehabilitation in neurological patients: state-of-art and future perspectives. Front Neurol. 2023;14:1255319. [doi: 10.3389/fneur.2023.1255319] [Medline: 37854065]

Piau A, Wild K, Mattek N, Kaye J. Current state of digital biomarker technologies for real-life, home-based monitoring of cognitive function for mild cognitive impairment to mild Alzheimer disease and implications for clinical care: systematic review. J Med Internet Res. Aug 30, 2019;21(8):e12785. [doi: 10.2196/12785] [Medline: 31471958]

Gaigher JM, Lacerda IB, Dourado MCN. Dementia and mental health during the COVID-19 pandemic: a systematic review. Front Psychiatry. 2022;13:879598. [doi: 10.3389/fpsyt.2022.879598] [Medline: 35873228]

Morgan D, Innes A, Kosteniuk J. Dementia care in rural and remote settings: a systematic review of formal or paid care. Maturitas. Jan 2011;68(1):17-33. [doi: 10.1016/j.maturitas.2010.09.008] [Medline: 21041045]

Barth J, Nickel F, Kolominsky-Rabas PL. Diagnosis of cognitive decline and dementia in rural areas - a scoping review. Int J Geriatr Psychiatry. Mar 2018;33(3):459-474. [doi: 10.1002/gps.4841] [Medline: 29314221]

Watt JA, Lane NE, Veroniki AA, et al. Diagnostic accuracy of virtual cognitive assessment and testing: systematic review and meta-analysis. J Am Geriatr Soc. Jun 2021;69(6):1429-1440. [doi: 10.1111/jgs.17190] [Medline: 33948937]

Costanzo MC, Arcidiacono C, Rodolico A, Panebianco M, Aguglia E, Signorelli MS. Diagnostic and interventional implications of telemedicine in Alzheimer’s disease and mild cognitive impairment: a literature review. Int J Geriatr Psychiatry. Jan 2020;35(1):12-28. [doi: 10.1002/gps.5219] [Medline: 31617247]

Beishon LC, Elliott E, Hietamies TM, et al. Diagnostic test accuracy of remote, multidomain cognitive assessment (telephone and video call) for dementia. Cochrane Database Syst Rev. Apr 8, 2022;4(4):CD013724. [doi: 10.1002/14651858.CD013724.pub2] [Medline: 35395108]

McCleery J, Laverty J, Quinn TJ. Diagnostic test accuracy of telehealth assessment for dementia and mild cognitive impairment. Cochrane Database Syst Rev. Jul 20, 2021;7(7):CD013786. [doi: 10.1002/14651858.CD013786.pub2] [Medline: 34282852]

Sohn M, Yang J, Sohn J, Lee JH. Digital healthcare for dementia and cognitive impairment: a scoping review. Int J Nurs Stud. Apr 2023;140:104413. [doi: 10.1016/j.ijnurstu.2022.104413] [Medline: 36821951]

Rai HK, Kernaghan D, Schoonmade L, Egan KJ, Pot AM. Digital technologies to prevent social isolation and loneliness in dementia: a systematic review. J Alzheimers Dis. 2022;90(2):513-528. [doi: 10.3233/JAD-220438] [Medline: 36120780]

Scerbe A, O’Connell ME, Astell A, et al. Digital tools for delivery of dementia education for caregivers of persons with dementia: a systematic review and meta-analysis of impact on caregiver distress and depressive symptoms. PLoS One. 2023;18(5):e0283600. [doi: 10.1371/journal.pone.0283600] [Medline: 37196022]

Binng D, Splonskowski M, Jacova C. Distance assessment for detecting cognitive impairment in older adults: a systematic review of psychometric evidence. Dement Geriatr Cogn Disord. 2020;49(5):456-470. [doi: 10.1159/000511945] [Medline: 33291097]

Naslund JA, Mitchell LM, Joshi U, Nagda D, Lu C. Economic evaluation and costs of telepsychiatry programmes: a systematic review. J Telemed Telecare. Jun 2022;28(5):311-330. [doi: 10.1177/1357633X20938919] [Medline: 32746762]

Zhao Q, Li C, Zhang Y, et al. Economic evaluations of electronic health interventions for people with age-related cognitive impairment and their caregivers: a systematic review. Int J Geriatr Psychiatry. Sep 2023;38(9):e5990. [doi: 10.1002/gps.5990] [Medline: 37655517]

Brims L, Oliver K. Effectiveness of assistive technology in improving the safety of people with dementia: a systematic review and meta-analysis. Aging Ment Health. Aug 3, 2019;23(8):942-951. [doi: 10.1080/13607863.2018.1455805]

Eaglestone G, Gkaintatzi E, Stoner C, Pacella R, McCrone P. Effectiveness of community non-pharmacological interventions for mild cognitive impairment and dementia: a systematic review of economic evaluations and a review of reviews. medRxiv. Preprint posted online on 2022. [doi: 10.1101/2022.12.16.22283561]

Vandepitte S, Van Den Noortgate N, Putman K, Verhaeghe S, Faes K, Annemans L. Effectiveness of supporting informal caregivers of people with dementia: a systematic review of randomized and non-randomized controlled trials. J Alzheimers Dis. Apr 8, 2016;52(3):929-965. [doi: 10.3233/JAD-151011] [Medline: 27079704]

Saragih ID, Tonapa SI, Porta CM, Lee B. Effects of telehealth intervention for people with dementia and their carers: a systematic review and meta‐analysis of randomized controlled studies. J Nurs Scholarsh. Nov 2022;54(6):704-719. URL: <https://sigmapubs.onlinelibrary.wiley.com/toc/15475069/54/6> [doi: 10.1111/jnu.12797]

Lins S, Hayder-Beichel D, Rücker G, et al. Efficacy and experiences of telephone counselling for informal carers of people with dementia. Cochrane Database Syst Rev. Sep 1, 2014;2014(9):CD009126. [doi: 10.1002/14651858.CD009126.pub2] [Medline: 25177838]

Durepos P, MacLean R, Ricketts N, et al. Engaging care partners of persons living with dementia in acceptance and commitment therapy (ACT) programs: a scoping review. Aging Ment Health. May 2024;28(5):725-737. [doi: 10.1080/13607863.2023.2288864] [Medline: 38100551]

Muirhead K, Macaden L, Smyth K, et al. Establishing the effectiveness of technology-enabled dementia education for health and social care practitioners: a systematic review. Syst Rev. Sep 21, 2021;10(1):252. [doi: 10.1186/s13643-021-01781-8] [Medline: 34548101]

Kruse CS, Fohn J, Umunnakwe G, Patel K, Patel S. Evaluating the facilitators, barriers, and medical outcomes commensurate with the use of assistive technology to support people with dementia: a systematic review literature. Healthcare (Basel). Aug 18, 2020;8(3):278. [doi: 10.3390/healthcare8030278] [Medline: 32824711]

Caprioli T, Mason S, Tetlow H, Reilly S, Giebel C. Exploring the views and the use of information and communication technologies to access post-diagnostic support by people living with dementia and unpaid carers: a systematic review. Aging Ment Health. Dec 2, 2023;27(12):2329-2345. [doi: 10.1080/13607863.2023.2196246]

Hung L, Wong J, Smith C, et al. Facilitators and barriers to using telepresence robots in aged care settings: a scoping review. J Rehabil Assist Technol Eng. 2022;9:20556683211072385. [doi: 10.1177/20556683211072385] [Medline: 35083063]

Gentry MT, Lapid MI, Rummans TA. Geriatric telepsychiatry: systematic review and policy considerations. Am J Geriatr Psychiatry. Feb 2019;27(2):109-127. [doi: 10.1016/j.jagp.2018.10.009] [Medline: 30416025]

Pinto-Bruno ÁC, García-Casal JA, Csipke E, Jenaro-Río C, Franco-Martín M. ICT-based applications to improve social health and social participation in older adults with dementia. A systematic literature review. Aging Ment Health. Jan 2017;21(1):58-65. [doi: 10.1080/13607863.2016.1262818] [Medline: 27936876]

Zhu EM, Buljac-Samardžić M, Ahaus K, Sevdalis N, Huijsman R. Implementation and dissemination of home- and community-based interventions for informal caregivers of people living with dementia: a systematic scoping review. Implement Sci. Nov 8, 2023;18(1):60. [doi: 10.1186/s13012-023-01314-y] [Medline: 37940960]

Coumoundouros C, Mårtensson E, Ferraris G, et al. Implementation of e-mental health interventions for informal caregivers of adults with chronic diseases: mixed methods systematic review with a qualitative comparative analysis and thematic synthesis. JMIR Ment Health. Nov 30, 2022;9(11):e41891. [doi: 10.2196/41891] [Medline: 36314782]

Gately ME, Trudeau SA, Moo LR. In-home video telehealth for dementia management: implications for rehabilitation. Curr Geriatr Rep. Sep 1, 2019;8(3):239-249. [doi: 10.1007/s13670-019-00297-3] [Medline: 32015957]

D’Onofrio G, Sancarlo D, Ricciardi F, et al. Information and communication technologies for the activities of daily living in older patients with dementia: a systematic review. J Alzheimers Dis. 2017;57(3):927-935. [doi: 10.3233/JAD-161145] [Medline: 28304297]

Pit SW, Horstmanshof L, Moehead A, Hayes O, Schache V, Parkinson L. International standards for dementia workforce education and training: a scoping review. Gerontologist. Feb 1, 2024;64(2):gnad023. [doi: 10.1093/geront/gnad023] [Medline: 37071967]

Spencer L, Potterton R, Allen K, Musiat P, Schmidt U. Internet-based interventions for carers of individuals with psychiatric disorders, neurological disorders, or brain injuries: systematic review. J Med Internet Res. Jul 9, 2019;21(7):e10876. [doi: 10.2196/10876] [Medline: 31290399]

Leng M, Zhao Y, Xiao H, Li C, Wang Z. Internet-based supportive interventions for family caregivers of people with dementia: systematic review and meta-analysis. J Med Internet Res. Sep 9, 2020;22(9):e19468. [doi: 10.2196/19468] [Medline: 32902388]

Gonella S, Mitchell G, Bavelaar L, et al. Interventions to support family caregivers of people with advanced dementia at the end of life in nursing homes: a mixed-methods systematic review. Palliat Med. Feb 2022;36(2):268-291. [doi: 10.1177/02692163211066733] [Medline: 34965759]

Müller C, Lautenschläger S, Meyer G, Stephan A. Interventions to support people with dementia and their caregivers during the transition from home care to nursing home care: a systematic review. Int J Nurs Stud. Jun 2017;71:139-152. [doi: 10.1016/j.ijnurstu.2017.03.013] [Medline: 28411508]

Kruse CS, Mileski ME, Wilkinson R, Hock B, Samson R, Castillo T. Leveraging technology to diagnose Alzheimer’s disease: a systematic review and meta-analysis. Healthcare (Basel). Nov 21, 2023;11(23):3013. [doi: 10.3390/healthcare11233013] [Medline: 38063581]

Brito S de, Scianni AA, Peniche P da C, Faria C de M. Measurement properties of outcome measures used in neurological telerehabilitation: a systematic review using COSMIN checklist. Clin Rehabil. Mar 2023;37(3):415-435. [doi: 10.1177/02692155221129834] [Medline: 36448251]

El-Saifi N, Moyle W, Jones C, Tuffaha H. Medication adherence in older patients with dementia: a systematic literature review. J Pharm Pract. Jun 2018;31(3):322-334. [doi: 10.1177/0897190017710524] [Medline: 28539102]

Bacanoiu MV, Danoiu M. New strategies to improve the quality of life for normal aging versus pathological aging. J Clin Med. Jul 20, 2022;11(14):4207. [doi: 10.3390/jcm11144207] [Medline: 35887969]

Lee DCA, Tirlea L, Haines TP. Non-pharmacological interventions to prevent hospital or nursing home admissions among community-dwelling older people with dementia: a systematic review and meta-analysis. Health Soc Care Community. Sep 2020;28(5):1408-1429. [doi: 10.1111/hsc.12984] [Medline: 32223022]

Amiri P, Niazkhani Z, Pirnejad H, ShojaeiBaghini M, Bahaadinbeigy K. Objectives, outcomes, facilitators, and barriers of telemedicine systems for patients with Alzheimer’s disease and their caregivers and care providers: a systematic review. Arch Iran Med. Aug 1, 2022;25(8):564-573. [doi: 10.34172/aim.2022.90] [Medline: 37543880]

Nissen RM, Serwe KM. Occupational therapy telehealth applications for the dementia-caregiver dyad: a scoping review. Phys Occup Ther Geriatr. Oct 2, 2018;36(4):366-379. [doi: 10.1080/02703181.2018.1536095]

Etxeberria I, Salaberria K, Gorostiaga A. Online support for family caregivers of people with dementia: a systematic review and meta-analysis of RCTs and quasi-experimental studies. Aging Ment Health. Jul 3, 2021;25(7):1165-1180. [doi: 10.1080/13607863.2020.1758900]

Egan KJ, Pinto-Bruno ÁC, Bighelli I, et al. Online training and support programs designed to improve mental health and reduce burden among caregivers of people with dementia: a systematic review. J Am Med Dir Assoc. Mar 2018;19(3):200-206. [doi: 10.1016/j.jamda.2017.10.023] [Medline: 29306605]

Elliot V, Morgan D, Kosteniuk J, et al. Palliative and end-of-life care for people living with dementia in rural areas: a scoping review. PLoS One. 2021;16(1):e0244976. [doi: 10.1371/journal.pone.0244976] [Medline: 33444351]

Boyle LD, Husebo BS, Vislapuu M. Promotors and barriers to the implementation and adoption of assistive technology and telecare for people with dementia and their caregivers: a systematic review of the literature. BMC Health Serv Res. Dec 23, 2022;22(1):1573. [doi: 10.1186/s12913-022-08968-2] [Medline: 36550456]

Elvish R, Lever SJ, Johnstone J, Cawley R, Keady J. Psychological interventions for carers of people with dementia: a systematic review of quantitative and qualitative evidence. Couns and Psychother Res. Jun 2013;13(2):106-125. [doi: 10.1080/14733145.2012.739632]

Hunter MB, Jenkins N, Dolan C, Pullen H, Ritchie C, Muniz-Terrera G. Reliability of telephone and videoconference methods of cognitive assessment in older adults with and without dementia. J Alzheimers Dis. 2021;81(4):1625-1647. [doi: 10.3233/JAD-210088] [Medline: 33967052]

González-Fraile E, Ballesteros J, Rueda JR, Santos-Zorrozúa B, Solà I, McCleery J. Remotely delivered information, training and support for informal caregivers of people with dementia. Cochrane Database Syst Rev. Jan 4, 2021;1(1):CD006440. [doi: 10.1002/14651858.CD006440.pub3] [Medline: 33417236]

Lin JS, O’Connor E, Rossom RC, Perdue LA, Eckstrom E. Screening for cognitive impairment in older adults: a systematic review for the U.S. Preventive Services Task Force. Ann Intern Med. Nov 5, 2013;159(9):601-612. [doi: 10.7326/0003-4819-159-9-201311050-00730] [Medline: 24145578]

Corbett A, Stevens J, Aarsland D, et al. Systematic review of services providing information and/or advice to people with dementia and/or their caregivers. Int J Geriat Psychiatry. Jun 2012;27(6):628-636. [doi: 10.1002/gps.2762]

Maresova P, Tomsone S, Lameski P, et al. Technological solutions for older people with Alzheimer’s disease: review. Curr Alzheimer Res. Aug 15, 2018;15(10):975-983. [doi: 10.2174/1567205015666180427124547]

Bauernschmidt D, Hirt J, Langer G, et al. Technology-based counselling for people with dementia and their informal carers: a systematic review and meta-analysis. J Alzheimers Dis. 2023;93(3):891-906. [doi: 10.3233/JAD-221194] [Medline: 37125549]

Mao W, Qi X, Chi I, Wichinsky L, Wu B. Technology-based interventions to address social isolation and loneliness among informal dementia caregivers: a scoping review. J Am Med Dir Assoc. Nov 2023;24(11):1700-1707. [doi: 10.1016/j.jamda.2023.08.005] [Medline: 37678415]

Zhu A, Cao W, Zhou Y, Xie A, Cheng Y, Chu SF. Tele-health intervention for carers of dementia patients-a systematic review and meta-analysis of randomized controlled trials. Front Aging Neurosci. 2021;13:612404. [doi: 10.3389/fnagi.2021.612404] [Medline: 33643022]

Carotenuto A, Traini E, Fasanaro AM, Battineni G, Amenta F. Tele-neuropsychological assessment of Alzheimer’s disease. J Pers Med. Jul 21, 2021;11(8):688. [doi: 10.3390/jpm11080688] [Medline: 34442332]

Ferreira Santana R, Vaqueiro Dantas R, et al. Telecare to elderly people with Alzheimer and their caregivers: systematic review. Cienc Cuid Saude. 2018;17:1-6. [doi: 10.4025/cienccuidsaude.v17i4.41653]

Söylemez BA, Özgül E, Küçükgüçlü Ö, Yener G. Telehealth applications used for self-efficacy levels of family caregivers for individuals with dementia: a systematic review and Meta-analysis. Geriatr Nurs (Lond). Jan 2023;49:178-192. [doi: 10.1016/j.gerinurse.2022.12.001]

Washington SE, Bollinger RM, Edwards E, McGowan L, Stephens S. Telehealth delivery of evidence-based intervention within older adult populations: a scoping review. OTJR (Thorofare N J). Jul 2023;43(3):467-477. [doi: 10.1177/15394492231180838] [Medline: 37322873]

Graven LJ, Glueckauf RL, Regal RA, Merbitz NK, Lustria MLA, James BA. Telehealth interventions for family caregivers of persons with chronic health conditions: a systematic review of randomized controlled trials. Int J Telemed Appl. 2021;2021:3518050. [doi: 10.1155/2021/3518050] [Medline: 34093704]

Yi JS, Pittman CA, Price CL, Nieman CL, Oh ES. Telemedicine and dementia care: a systematic review of barriers and facilitators. J Am Med Dir Assoc. Jul 2021;22(7):1396-1402. [doi: 10.1016/j.jamda.2021.03.015] [Medline: 33887231]

Sekhon H, Sekhon K, Launay C, et al. Telemedicine and the rural dementia population: a systematic review. Maturitas. Jan 2021;143:105-114. [doi: 10.1016/j.maturitas.2020.09.001] [Medline: 33308615]

León-Salas B, González-Hernández Y, Infante-Ventura D, et al. Telemedicine for neurological diseases: a systematic review and meta-analysis. Eur J Neurol. Jan 2023;30(1):241-254. [doi: 10.1111/ene.15599] [Medline: 36256522]

Folder N, Power E, Rietdijk R, Christensen I, Togher L, Parker D. The effectiveness and characteristics of communication partner training programs for families of people with dementia: a systematic review. Gerontologist. Apr 1, 2024;64(4):gnad095. [doi: 10.1093/geront/gnad095] [Medline: 37439771]

Yu Y, Xiao L, Ullah S, et al. The effectiveness of internet-based psychoeducation programs for caregivers of people living with dementia: a systematic review and meta-analysis. Aging Ment Health. 2023;27(10):1895-1911. [doi: 10.1080/13607863.2023.2190082] [Medline: 36951611]

Hailey D, Roine R, Ohinmaa A. The effectiveness of telemental health applications: a review. Can J Psychiatry. Nov 2008;53(11):769-778. [doi: 10.1177/070674370805301109] [Medline: 19087471]

Lucero RJ, Fehlberg EA, Patel AGM, et al. The effects of information and communication technologies on informal caregivers of persons living with dementia: a systematic review. Alzheimers Dement (N Y). 2018;5:1-12. [doi: 10.1016/j.trci.2018.11.003] [Medline: 30623020]

Rueda Daz LJ, Monteiro da Cruz DL. The efficacy of telephone use to assist and improve the wellbeing of family caregivers of persons with chronic diseases: a systematic review. JBI Database System Rev Implement Rep. Dec 2014;12(12):106-140. [doi: 10.11124/jbisrir-2014-1566]

Nkodo JA, Gana W, Debacq C, et al. The role of telemedicine in the management of the behavioral and psychological symptoms of dementia: a systematic review. Am J Geriatr Psychiatry. Oct 2022;30(10):1135-1150. [doi: 10.1016/j.jagp.2022.01.013] [Medline: 35241355]

Liang J, Aranda MP. The use of telehealth among people living with dementia-caregiver dyads during the COVID-19 pandemic: scoping review. J Med Internet Res. May 25, 2023;25:e45045. [doi: 10.2196/45045] [Medline: 37227755]

Armstrong MJ, Alliance S. Virtual support groups for informal caregivers of individuals with dementia: a scoping review. Alzheimer Dis Assoc Disord. 2019;33(4):362-369. [doi: 10.1097/WAD.0000000000000349] [Medline: 31567144]

Wood M, Walshe C, McCullagh A. What are the digitally enabled psychosocial interventions delivered by trained practitioners being offered to adults with life-shortening illnesses and palliative care needs and their informal and professional caregivers? A scoping review. Palliat Support Care. Aug 2023;21(4):727-740. [doi: 10.1017/S1478951523000172] [Medline: 36994819]

Sun Y, Ji M, Leng M, Wang Z. Which cognitive behavioral therapy delivery formats work for depressive symptoms in dementia caregivers? - A systematic review and network meta-analysis of randomized controlled trials. J Affect Disord. Jul 1, 2022;308:181-187. [doi: 10.1016/j.jad.2022.04.055] [Medline: 35429541]

Kishita N, Hammond L, Dietrich CM, Mioshi E. Which interventions work for dementia family carers?: an updated systematic review of randomized controlled trials of carer interventions. Int Psychogeriatr. Nov 2018;30(11):1679-1696. [doi: 10.1017/S1041610218000947] [Medline: 30017008]
