# Supplementary material for: An Overview of Reviews on Telemedicine and Telehealth in Dementia Care: Mixed Methods Synthesis
Source: JMIR Ment Health. 2025 Nov 6;12:e75266. doi: 10.2196/75266 (PMC12975415; doi:10.2196/75266)
Supplement: Multimedia Appendix 3 [file mental-v12-e75266-s003.docx]

| **Table S2. Reporting completeness among included systematic reviews using the AMSTAR-2 tool** | | | | | | | | | | | | | | | | | | | |
| --- | --- | --- | --- | --- | --- | --- | --- | --- | --- | --- | --- | --- | --- | --- | --- | --- | --- | --- | --- |
| **Review ID** | **1** | **2*** | **3** | **4** | **5** | **6** | **7*** | **8** | **9a*** | **9b*** | **10** | **11a*** | **11b*** | **12** | **13*** | **14** | **15*** | **16** | **Overall confidence** |
| Amiri 2022 | + | - | + | P | + | - | - | - | - | - | - | NA | NA | NA | - | - | NA | + | Critically low |
| Bacanoiu 2022 | + | - | + | P | - | - | - | + | + | + | - | NA | NA | NA | - | - | NA | + | Critically low |
| Bauernschmidt D. 2023 | + | + | + | P | + | + | - | + | + | NA | + | + | NA | + | + | + | - | + | Critically Low |
| Beishon L.C. 2022 | + | + | + | P | + | + | + | + | NA | + | - | NA | NA | NA | + | + | + | + | Moderate |
| Binng, Davina 2020 | + | - | + | P | + | - | - | - | NA | - | - | NA | NA | NA | - | + | - | + | Critically Low |
| Boyle L.D. 2022 | + | P | + | P | + | + | - | - | P | - | - | NA | NA | NA | + | + | NA | + | Critically Low |
| Brims L. 2019 | + | P | + | P | + | - | P | P | + | NA | - | + | NA | + | + | + | - | + | Low |
| Brito S.A.F. 2023 | + | P | - | P | + | + | - | - | - | - | + | NA | NA | NA | + | - | NA | + | Critically low |
| Caprioli T. 2023 | + | + | + | P | - | - | P | P | - | - | - | NA | NA | NA | - | - | NA | + | Critically low |
| Carotenuto A. 2021 | - | - | + | P | - | - | - | + | + | NA | - | NA | NA | NA | + | - | NA | + | Critically low |
| Corbett A. 2012 | + | P | + | + | - | - | P | + | + | NA | - | + | NA | + | + | + | - | + | Low |
| Costanzo M.C. 2020 | + | - | + | P | + | - | - | + | - | NA | - | NA | NA | NA | - | + | - | + | Critically low |
| Cotelli M .2019 | + | P | - | + | + | - | - | + | + | - | - | NA | NA | NA | + | + | NA | + | Critically low |
| Coumoundouros C. 2022 | + | + | + | + | + | + | + | + | + | NA | - | + | NA | - | + | - | - | + | Low |
| D’onofrio G. 2017 | + | - | + | + | P | - | - | - | - | - | - | NA | NA | NA | - | + | NA | + | Critically low |
| Daly Lynn J. 2019 | + | - | + | P | - | + | - | P | - | - | - | NA | NA | NA | - | + | NA | + | Critically low |
| Dam A.E.H 2016 | + | - | + | + | + | - | + | P | + | NA | - | NA | NA | NA | + | + | NA | + | Critically low |
| Di Lorito C. 2022 | + | - | + | - | + | + | - | P | + | NA | - | + | NA | + | + | + | - | - | Critically low |
| Egan K.J. 2018 | + | P | + | P | + | - | - | + | + | NA | - | NA | NA | NA | + | + | - | + | Low |
| Eaglestone G. 2022 | + | + | - | P | + | - | - | P | NA | NA | - | NA | NA | NA | - | - | NA | + | Critically low |
| El-Saifi N. 2018 | + | - | - | - | + | - | - | P | - | - | - | NA | NA | NA | - | - | NA | + | Critically low |
| Elbaz S. 2021 | - | - | - | P | + | - | - | P | - | - | - | NA | NA | NA | - | - | NA | + | Critically low |
| Elliott E. 2020 | + | P | - | P | + | + | - | P | + | + | - | + | + | - | + | - | - | + | Critically low |
| Elvish R. 2013 | + | - | - | - | - | + | - | P | P | P | - | NA | NA | NA | - | - | NA | - | Critically low |
| Etxeberria I. 2021 | + | - | - | - | + | - | - | P | - | - | - | + | - | + | + | + | - | + | Critically low |
| Ferreira Santana, R 2018 | + | - | - | - | - | - | - | P | - | - | - | NA | NA | NA | - | - | NA | - | Critically low |
| Folder N. 2024 | + | + | - | - | + | - | - | P | + | + | - | NA | NA | NA | - | - | NA | + | Critically low |
| Gaigher J.M. 2022 | + | P | - | - | + | - | - | P | + | + | - | NA | NA | NA | - | - | NA | - | Critically low |
| Gentry M.T. 2019 | + | - | - | - | - | - | - | P | - | - | - | NA | NA | NA | - | - | NA | + | Critically low |
| Gonella S. 2022 | + | + | + | + | + | + | - | P | + | + | + | NA | NA | NA | + | - | NA | + | Low |
| Gonzalez-Fraile E. 2021 | + | + | + | P | + | + | + | + | + | NA | + | + | NA | + | + | + | - | + | High |
| Graven L.J. 2021 | + | - | - | - | + | + | - | P | - | NA | - | NA | NA | NA | - | - | NA | + | Critically low |
| Hailey D. 2008 | - | - | - | P | + | + | - | - | P | P | - | NA | NA | NA | + | - | NA | + | Critically low |
| Hunter M.B. 2021 | + | - | - | - | + | + | - | - | - | - | - | NA | NA | NA | - | - | NA | + | Critically low |
| Jackson D. 2016 | + | - | - | P | - | - | - | P | + | - | - | NA | NA | NA | - | - | NA | + | Critically low |
| Kishita N. 2018 | + | - | - | - | + | + | - | - | - | - | - | - | - | - | - | - | - | + | Critically low |
| Kruse, Clemens Scott 2020 | + | P | - | - | + | + | - | P | - | - | - | NA | NA | NA | - | - | NA | + | Critically low |
| Kruse, Clemens Scott 2023 | + | - | - | P | + | - | - | - | - | - | - | - | - | - | - | - | - | + | Critically Low |
| Kwan R.Y.-C. 2013 | + | - | + | - | - | - | - | - | NA | - | - | NA | NA | NA | - | + | NA | + | Critically Low |
| Lee D.-C.A. 2020 | + | P | + | P | + | - | - | + | + | + | - | - | + | - | + | + | - | + | Critically Low |
| Leng M. 2020 | + | + | + | P | + | + | P | + | + | + | - | + | + | + | - | - | + | + | Low |
| Leon-Salas B. 2023 | + | + | - | P | + | + | + | + | + | NA | - | + | NA | - | + | + | - | + | Low |
| Lin J.S.2013 | + | + | + | P | + | + | P | P | + | + | - | + | + | + | + | + | + | + | High |
| Lins S. 2014 | + | + | + | + | + | + | + | + | + | NA | + | + | NA | + | + | + | + | + | High |
| Lucero R.J. 2019 | + | P | + | P | + | + | - | P | + | + | + | NA | NA | NA | + | - | NA | + | Low |
| Martin-Khan M. 2010 | + | - | - | P | - | - | - | - | NA | - | - | NA | NA | NA | - | - | NA | + | Critically Low |
| Martínez-Alcalá 2015 | + | - | + | P | - | - | - | - | NA | - | - | NA | NA | NA | - | - | NA | - | Critically Low |
| McCleery J. 2021 | + | + | + | P | + | + | + | + | NA | + | - | NA | NA | NA | + | + | NA | + | Moderate |
| Morgan D. 2011 | + | - | - | P | + | - | - | P | NA | - | - | NA | NA | NA | - | - | NA | + | Critically Low |
| Muirhead K. 2021 | + | P | + | + | + | - | P | P | + | + | - | NA | NA | NA | + | - | NA | + | Moderate |
| Muller C. 2017 | + | P | - | P | + | + | P | + | + | + | - | NA | NA | NA | + | + | NA | + | Moderate |
| Naslund J.A. 2022 | + | - | + | P | + | + | - | + | - | - | - | NA | NA | NA | - | + | NA | + | Critically Low |
| Nkodo J.-A. 2022 | - | - | - | - | - | - | - | P | - | - | - | NA | NA | NA | - | - | NA | + | Critically low |
| Piau A. 2019 | + | - | - | - | + | - | - | P | - | - | - | NA | NA | NA | - | - | NA | + | Critically low |
| Pinto-Bruno AC. 2017 | + | - | - | P | + | + | - | P | NA | - | - | NA | NA | NA | - | + | NA | + | Critically low |
| Rai H.K. 2022 | + | P | + | P | + | + | - | + | P | + | - | NA | NA | NA | + | + | NA | + | Low |
| Riley C.O. 2022 | + | - | - | - | + | + | + | - | - | - | - | NA | NA | NA | - | - | NA | + | Critically Low |
| Rueda Daz L.J. 2014 | + | - | + | P | - | - | P | P | P | NA | - | NA | NA | NA | + | + | NA | + | Low |
| Söylemez BA 2022 | + | + | - | P | + | + | P | - | - | - | - | - | - | - | - | - | - | + | Critically Low |
| Saragih I.D. 2022 | + | + | - | - | + | + | - | P | + | NA | - | - | NA | - | - | + | + | + | Critically Low |
| Scerbe A. 2023 | + | P | - | P | + | + | - | P | + | + | - | - | NA | + | + | - | - | + | Critically Low |
| Sekhon H. 2021 | - | - | - | P | - | - | - | + | - | - | - | NA | NA | NA | - | - | NA | + | Critically Low |
| Spencer L. 2019 | + | - | - | - | + | + | - | - | + | P | - | NA | NA | NA | + | - | NA | + | Critically Low |
| Sun Y. 2022 | + | + | + | + | + | + | + | + | + | + | - | + | + | + | + | + | + | + | High |
| van der Wardt V. 2017 | + | P | + | P | - | - | - | - | - | - | - | NA | NA | NA | - | - | NA | + | Critically Low |
| Vandepitte, Sophie 2016 | + | - | + | - | - | - | - | P | + | + | - | NA | NA | NA | + | + | NA | + | Critically Low |
| Waller A. 2017 | + | - | + | P | - | - | - | + | + | + | - | NA | NA | NA | + | - | NA | + | Critically Low |
| Watt J.A. 2021 | + | + | + | + | + | + | - | + | + | + | - | + | + | + | + | - | - | + | Critically Low |
| Yi J.S. 2021 | + | - | + | + | + | + | - | + | + | + | - | NA | NA | NA | + | + | NA | + | Critically Low |
| Yu Y. 2023 | + | + | + | + | + | + | - | + | + | + | - | + | + | + | + | + | - | + | Critically Low |
| Zhao Q. 2023 | + | - | + | P | + | + | - | + | + | NA | + | NA | NA | NA | + | + | NA | + | Critically Low |
| Zhu A. 2021 | + | - | + | + | + | + | - | + | + | + | - | + | + | + | + | - | + | + | Critically Low |

**Reference list of the 91 included studies**

Di Lorito C, Bosco A, Rai H, et al. A systematic literature review and meta-analysis on digital health interventions for people living with dementia and mild cognitive impairment. Int J Geriatr Psychiatry. Jun 2022;37(6). [doi: 10.1002/gps.5730] [Medline: 35588315]

Daly Lynn J, Rondón-Sulbarán J, Quinn E, Ryan A, McCormack B, Martin S. A systematic review of electronic assistive technology within supporting living environments for people with dementia. Dementia (London). 2019;18(7-8):2371-2435. [doi: 10.1177/1471301217733649] [Medline: 28990408]

Dam AEH, de Vugt ME, Klinkenberg IPM, Verhey FRJ, van Boxtel MPJ. A systematic review of social support interventions for caregivers of people with dementia: are they doing what they promise? Maturitas. Mar 2016;85:117-130. [doi: 10.1016/j.maturitas.2015.12.008] [Medline: 26857890]

Elbaz S, Cinalioglu K, Sekhon K, et al. A systematic review of telemedicine for older adults with dementia during COVID-19: an alternative to in-person health services? Front Neurol. 2021;12:761965. [doi: 10.3389/fneur.2021.761965] [Medline: 34970210]

Jackson D, Roberts G, Wu ML, Ford R, Doyle C. A systematic review of the effect of telephone, internet or combined support for carers of people living with Alzheimer’s, vascular or mixed dementia in the community. Arch Gerontol Geriatr. 2016;66:218-236. [doi: 10.1016/j.archger.2016.06.013] [Medline: 27372903]

Martin-Khan M, Wootton R, Gray L. A systematic review of the reliability of screening for cognitive impairment in older adults by use of standardised assessment tools administered via the telephone. J Telemed Telecare. 2010;16(8):422-428. [doi: 10.1258/jtt.2010.100209] [Medline: 21030488]

Riley CO, McKinstry B, Fairhurst K. Accuracy of telephone screening tools to identify dementia patients remotely: systematic review. JRSM Open. Sep 2022;13(9):20542704221115956. [doi: 10.1177/20542704221115956] [Medline: 36082188]

Elliott E, Green C, Llewellyn DJ, Quinn TJ. Accuracy of telephone-based cognitive screening tests: systematic review and meta-analysis. Curr Alzheimer Res. 2020;17(5):460-471. [doi: 10.2174/1567205017999200626201121] [Medline: 32589557]

van der Wardt V, Hancox J, Gondek D, et al. Adherence support strategies for exercise interventions in people with mild cognitive impairment and dementia: a systematic review. Prev Med Rep. Sep 2017;7:38-45. [doi: 10.1016/j.pmedr.2017.05.007] [Medline: 28593121]

Martinez-Alcala CI, Pliego-Pastrana P, Lopez-Noguerola JS, Rosales-Lagarde A, Zaleta-Arias ME. Adoption of ICT in the aging: systematic review based on ICT for Alzheimer’s disease and other senile dementias. Presented at: 2015 10th Iberian Conference on Information Systems and Technologies (CISTI); Jun 17-20, 2015; Aveiro, Portugal. [doi: 10.1109/CISTI.2015.7170393]

Gagnon-Roy M, Bourget A, Stocco S, Courchesne ACL, Kuhne N, Provencher V. Assistive technology addressing safety issues in dementia: a scoping review. Am J Occup Ther. 2017;71(5):7105190020p1-7105190020p10. [doi: 10.5014/ajot.2017.025817] [Medline: 28809655]

Kwan RYC, Lai CKY. Can smartphones enhance telephone-based cognitive assessment (TBCA)? Int J Environ Res Public Health. Dec 12, 2013;10(12):7110-7125. [doi: 10.3390/ijerph10127110] [Medline: 24351736]

Lin X, Ward SA, Pritchard E, et al. Carer-reported measures for a dementia registry: a systematic scoping review and a qualitative study. Australas J Ageing. Mar 2023;42(1):34-52. [doi: 10.1111/ajag.13148] [Medline: 36383194]

Cotelli M, Manenti R, Brambilla M, et al. Cognitive telerehabilitation in mild cognitive impairment, Alzheimer’s disease and frontotemporal dementia: a systematic review. J Telemed Telecare. Feb 2019;25(2):67-79. [doi: 10.1177/1357633X17740390] [Medline: 29117794]

Dedzoe JDS, Malmgren Fänge A, Christensen J, Lethin C. Collaborative learning through a virtual community of practice in dementia care support: a scoping review. Healthcare (Basel). Feb 26, 2023;11(5):692. [doi: 10.3390/healthcare11050692] [Medline: 36900696]

Waller A, Dilworth S, Mansfield E, Sanson-Fisher R. Computer and telephone delivered interventions to support caregivers of people with dementia: a systematic review of research output and quality. BMC Geriatr. Nov 16, 2017;17(1):265. [doi: 10.1186/s12877-017-0654-6] [Medline: 29145806]

Maggio MG, De Bartolo D, Calabrò RS, et al. Computer-assisted cognitive rehabilitation in neurological patients: state-of-art and future perspectives. Front Neurol. 2023;14:1255319. [doi: 10.3389/fneur.2023.1255319] [Medline: 37854065]

Piau A, Wild K, Mattek N, Kaye J. Current state of digital biomarker technologies for real-life, home-based monitoring of cognitive function for mild cognitive impairment to mild Alzheimer disease and implications for clinical care: systematic review. J Med Internet Res. Aug 30, 2019;21(8):e12785. [doi: 10.2196/12785] [Medline: 31471958]

Gaigher JM, Lacerda IB, Dourado MCN. Dementia and mental health during the COVID-19 pandemic: a systematic review. Front Psychiatry. 2022;13:879598. [doi: 10.3389/fpsyt.2022.879598] [Medline: 35873228]

Morgan D, Innes A, Kosteniuk J. Dementia care in rural and remote settings: a systematic review of formal or paid care. Maturitas. Jan 2011;68(1):17-33. [doi: 10.1016/j.maturitas.2010.09.008] [Medline: 21041045]

Barth J, Nickel F, Kolominsky-Rabas PL. Diagnosis of cognitive decline and dementia in rural areas - a scoping review. Int J Geriatr Psychiatry. Mar 2018;33(3):459-474. [doi: 10.1002/gps.4841] [Medline: 29314221]

Watt JA, Lane NE, Veroniki AA, et al. Diagnostic accuracy of virtual cognitive assessment and testing: systematic review and meta-analysis. J Am Geriatr Soc. Jun 2021;69(6):1429-1440. [doi: 10.1111/jgs.17190] [Medline: 33948937]

Costanzo MC, Arcidiacono C, Rodolico A, Panebianco M, Aguglia E, Signorelli MS. Diagnostic and interventional implications of telemedicine in Alzheimer’s disease and mild cognitive impairment: a literature review. Int J Geriatr Psychiatry. Jan 2020;35(1):12-28. [doi: 10.1002/gps.5219] [Medline: 31617247]

Beishon LC, Elliott E, Hietamies TM, et al. Diagnostic test accuracy of remote, multidomain cognitive assessment (telephone and video call) for dementia. Cochrane Database Syst Rev. Apr 8, 2022;4(4):CD013724. [doi: 10.1002/14651858.CD013724.pub2] [Medline: 35395108]

McCleery J, Laverty J, Quinn TJ. Diagnostic test accuracy of telehealth assessment for dementia and mild cognitive impairment. Cochrane Database Syst Rev. Jul 20, 2021;7(7):CD013786. [doi: 10.1002/14651858.CD013786.pub2] [Medline: 34282852]

Sohn M, Yang J, Sohn J, Lee JH. Digital healthcare for dementia and cognitive impairment: a scoping review. Int J Nurs Stud. Apr 2023;140:104413. [doi: 10.1016/j.ijnurstu.2022.104413] [Medline: 36821951]

Rai HK, Kernaghan D, Schoonmade L, Egan KJ, Pot AM. Digital technologies to prevent social isolation and loneliness in dementia: a systematic review. J Alzheimers Dis. 2022;90(2):513-528. [doi: 10.3233/JAD-220438] [Medline: 36120780]

Scerbe A, O’Connell ME, Astell A, et al. Digital tools for delivery of dementia education for caregivers of persons with dementia: a systematic review and meta-analysis of impact on caregiver distress and depressive symptoms. PLoS One. 2023;18(5):e0283600. [doi: 10.1371/journal.pone.0283600] [Medline: 37196022]

Binng D, Splonskowski M, Jacova C. Distance assessment for detecting cognitive impairment in older adults: a systematic review of psychometric evidence. Dement Geriatr Cogn Disord. 2020;49(5):456-470. [doi: 10.1159/000511945] [Medline: 33291097]

Naslund JA, Mitchell LM, Joshi U, Nagda D, Lu C. Economic evaluation and costs of telepsychiatry programmes: a systematic review. J Telemed Telecare. Jun 2022;28(5):311-330. [doi: 10.1177/1357633X20938919] [Medline: 32746762]

Zhao Q, Li C, Zhang Y, et al. Economic evaluations of electronic health interventions for people with age-related cognitive impairment and their caregivers: a systematic review. Int J Geriatr Psychiatry. Sep 2023;38(9):e5990. [doi: 10.1002/gps.5990] [Medline: 37655517]

Brims L, Oliver K. Effectiveness of assistive technology in improving the safety of people with dementia: a systematic review and meta-analysis. Aging Ment Health. Aug 3, 2019;23(8):942-951. [doi: 10.1080/13607863.2018.1455805]

Eaglestone G, Gkaintatzi E, Stoner C, Pacella R, McCrone P. Effectiveness of community non-pharmacological interventions for mild cognitive impairment and dementia: a systematic review of economic evaluations and a review of reviews. medRxiv. Preprint posted online on 2022. [doi: 10.1101/2022.12.16.22283561]

Vandepitte S, Van Den Noortgate N, Putman K, Verhaeghe S, Faes K, Annemans L. Effectiveness of supporting informal caregivers of people with dementia: a systematic review of randomized and non-randomized controlled trials. J Alzheimers Dis. Apr 8, 2016;52(3):929-965. [doi: 10.3233/JAD-151011] [Medline: 27079704]

Saragih ID, Tonapa SI, Porta CM, Lee B. Effects of telehealth intervention for people with dementia and their carers: a systematic review and meta‐analysis of randomized controlled studies. J Nurs Scholarsh. Nov 2022;54(6):704-719. URL: <https://sigmapubs.onlinelibrary.wiley.com/toc/15475069/54/6> [doi: 10.1111/jnu.12797]

Lins S, Hayder-Beichel D, Rücker G, et al. Efficacy and experiences of telephone counselling for informal carers of people with dementia. Cochrane Database Syst Rev. Sep 1, 2014;2014(9):CD009126. [doi: 10.1002/14651858.CD009126.pub2] [Medline: 25177838]

Durepos P, MacLean R, Ricketts N, et al. Engaging care partners of persons living with dementia in acceptance and commitment therapy (ACT) programs: a scoping review. Aging Ment Health. May 2024;28(5):725-737. [doi: 10.1080/13607863.2023.2288864] [Medline: 38100551]

Muirhead K, Macaden L, Smyth K, et al. Establishing the effectiveness of technology-enabled dementia education for health and social care practitioners: a systematic review. Syst Rev. Sep 21, 2021;10(1):252. [doi: 10.1186/s13643-021-01781-8] [Medline: 34548101]

Kruse CS, Fohn J, Umunnakwe G, Patel K, Patel S. Evaluating the facilitators, barriers, and medical outcomes commensurate with the use of assistive technology to support people with dementia: a systematic review literature. Healthcare (Basel). Aug 18, 2020;8(3):278. [doi: 10.3390/healthcare8030278] [Medline: 32824711]

Caprioli T, Mason S, Tetlow H, Reilly S, Giebel C. Exploring the views and the use of information and communication technologies to access post-diagnostic support by people living with dementia and unpaid carers: a systematic review. Aging Ment Health. Dec 2, 2023;27(12):2329-2345. [doi: 10.1080/13607863.2023.2196246]

Hung L, Wong J, Smith C, et al. Facilitators and barriers to using telepresence robots in aged care settings: a scoping review. J Rehabil Assist Technol Eng. 2022;9:20556683211072385. [doi: 10.1177/20556683211072385] [Medline: 35083063]

Gentry MT, Lapid MI, Rummans TA. Geriatric telepsychiatry: systematic review and policy considerations. Am J Geriatr Psychiatry. Feb 2019;27(2):109-127. [doi: 10.1016/j.jagp.2018.10.009] [Medline: 30416025]

Pinto-Bruno ÁC, García-Casal JA, Csipke E, Jenaro-Río C, Franco-Martín M. ICT-based applications to improve social health and social participation in older adults with dementia. A systematic literature review. Aging Ment Health. Jan 2017;21(1):58-65. [doi: 10.1080/13607863.2016.1262818] [Medline: 27936876]

Zhu EM, Buljac-Samardžić M, Ahaus K, Sevdalis N, Huijsman R. Implementation and dissemination of home- and community-based interventions for informal caregivers of people living with dementia: a systematic scoping review. Implement Sci. Nov 8, 2023;18(1):60. [doi: 10.1186/s13012-023-01314-y] [Medline: 37940960]

Coumoundouros C, Mårtensson E, Ferraris G, et al. Implementation of e-mental health interventions for informal caregivers of adults with chronic diseases: mixed methods systematic review with a qualitative comparative analysis and thematic synthesis. JMIR Ment Health. Nov 30, 2022;9(11):e41891. [doi: 10.2196/41891] [Medline: 36314782]

Gately ME, Trudeau SA, Moo LR. In-home video telehealth for dementia management: implications for rehabilitation. Curr Geriatr Rep. Sep 1, 2019;8(3):239-249. [doi: 10.1007/s13670-019-00297-3] [Medline: 32015957]

D’Onofrio G, Sancarlo D, Ricciardi F, et al. Information and communication technologies for the activities of daily living in older patients with dementia: a systematic review. J Alzheimers Dis. 2017;57(3):927-935. [doi: 10.3233/JAD-161145] [Medline: 28304297]

Pit SW, Horstmanshof L, Moehead A, Hayes O, Schache V, Parkinson L. International standards for dementia workforce education and training: a scoping review. Gerontologist. Feb 1, 2024;64(2):gnad023. [doi: 10.1093/geront/gnad023] [Medline: 37071967]

Spencer L, Potterton R, Allen K, Musiat P, Schmidt U. Internet-based interventions for carers of individuals with psychiatric disorders, neurological disorders, or brain injuries: systematic review. J Med Internet Res. Jul 9, 2019;21(7):e10876. [doi: 10.2196/10876] [Medline: 31290399]

Leng M, Zhao Y, Xiao H, Li C, Wang Z. Internet-based supportive interventions for family caregivers of people with dementia: systematic review and meta-analysis. J Med Internet Res. Sep 9, 2020;22(9):e19468. [doi: 10.2196/19468] [Medline: 32902388]

Gonella S, Mitchell G, Bavelaar L, et al. Interventions to support family caregivers of people with advanced dementia at the end of life in nursing homes: a mixed-methods systematic review. Palliat Med. Feb 2022;36(2):268-291. [doi: 10.1177/02692163211066733] [Medline: 34965759]

Müller C, Lautenschläger S, Meyer G, Stephan A. Interventions to support people with dementia and their caregivers during the transition from home care to nursing home care: a systematic review. Int J Nurs Stud. Jun 2017;71:139-152. [doi: 10.1016/j.ijnurstu.2017.03.013] [Medline: 28411508]

Kruse CS, Mileski ME, Wilkinson R, Hock B, Samson R, Castillo T. Leveraging technology to diagnose Alzheimer’s disease: a systematic review and meta-analysis. Healthcare (Basel). Nov 21, 2023;11(23):3013. [doi: 10.3390/healthcare11233013] [Medline: 38063581]

Brito S de, Scianni AA, Peniche P da C, Faria C de M. Measurement properties of outcome measures used in neurological telerehabilitation: a systematic review using COSMIN checklist. Clin Rehabil. Mar 2023;37(3):415-435. [doi: 10.1177/02692155221129834] [Medline: 36448251]

El-Saifi N, Moyle W, Jones C, Tuffaha H. Medication adherence in older patients with dementia: a systematic literature review. J Pharm Pract. Jun 2018;31(3):322-334. [doi: 10.1177/0897190017710524] [Medline: 28539102]

Bacanoiu MV, Danoiu M. New strategies to improve the quality of life for normal aging versus pathological aging. J Clin Med. Jul 20, 2022;11(14):4207. [doi: 10.3390/jcm11144207] [Medline: 35887969]

Lee DCA, Tirlea L, Haines TP. Non-pharmacological interventions to prevent hospital or nursing home admissions among community-dwelling older people with dementia: a systematic review and meta-analysis. Health Soc Care Community. Sep 2020;28(5):1408-1429. [doi: 10.1111/hsc.12984] [Medline: 32223022]

Amiri P, Niazkhani Z, Pirnejad H, ShojaeiBaghini M, Bahaadinbeigy K. Objectives, outcomes, facilitators, and barriers of telemedicine systems for patients with Alzheimer’s disease and their caregivers and care providers: a systematic review. Arch Iran Med. Aug 1, 2022;25(8):564-573. [doi: 10.34172/aim.2022.90] [Medline: 37543880]

Nissen RM, Serwe KM. Occupational therapy telehealth applications for the dementia-caregiver dyad: a scoping review. Phys Occup Ther Geriatr. Oct 2, 2018;36(4):366-379. [doi: 10.1080/02703181.2018.1536095]

Etxeberria I, Salaberria K, Gorostiaga A. Online support for family caregivers of people with dementia: a systematic review and meta-analysis of RCTs and quasi-experimental studies. Aging Ment Health. Jul 3, 2021;25(7):1165-1180. [doi: 10.1080/13607863.2020.1758900]

Egan KJ, Pinto-Bruno ÁC, Bighelli I, et al. Online training and support programs designed to improve mental health and reduce burden among caregivers of people with dementia: a systematic review. J Am Med Dir Assoc. Mar 2018;19(3):200-206. [doi: 10.1016/j.jamda.2017.10.023] [Medline: 29306605]

Elliot V, Morgan D, Kosteniuk J, et al. Palliative and end-of-life care for people living with dementia in rural areas: a scoping review. PLoS One. 2021;16(1):e0244976. [doi: 10.1371/journal.pone.0244976] [Medline: 33444351]

Boyle LD, Husebo BS, Vislapuu M. Promotors and barriers to the implementation and adoption of assistive technology and telecare for people with dementia and their caregivers: a systematic review of the literature. BMC Health Serv Res. Dec 23, 2022;22(1):1573. [doi: 10.1186/s12913-022-08968-2] [Medline: 36550456]

Elvish R, Lever SJ, Johnstone J, Cawley R, Keady J. Psychological interventions for carers of people with dementia: a systematic review of quantitative and qualitative evidence. Couns and Psychother Res. Jun 2013;13(2):106-125. [doi: 10.1080/14733145.2012.739632]

Hunter MB, Jenkins N, Dolan C, Pullen H, Ritchie C, Muniz-Terrera G. Reliability of telephone and videoconference methods of cognitive assessment in older adults with and without dementia. J Alzheimers Dis. 2021;81(4):1625-1647. [doi: 10.3233/JAD-210088] [Medline: 33967052]

González-Fraile E, Ballesteros J, Rueda JR, Santos-Zorrozúa B, Solà I, McCleery J. Remotely delivered information, training and support for informal caregivers of people with dementia. Cochrane Database Syst Rev. Jan 4, 2021;1(1):CD006440. [doi: 10.1002/14651858.CD006440.pub3] [Medline: 33417236]

Lin JS, O’Connor E, Rossom RC, Perdue LA, Eckstrom E. Screening for cognitive impairment in older adults: a systematic review for the U.S. Preventive Services Task Force. Ann Intern Med. Nov 5, 2013;159(9):601-612. [doi: 10.7326/0003-4819-159-9-201311050-00730] [Medline: 24145578]

Corbett A, Stevens J, Aarsland D, et al. Systematic review of services providing information and/or advice to people with dementia and/or their caregivers. Int J Geriat Psychiatry. Jun 2012;27(6):628-636. [doi: 10.1002/gps.2762]

Maresova P, Tomsone S, Lameski P, et al. Technological solutions for older people with Alzheimer’s disease: review. Curr Alzheimer Res. Aug 15, 2018;15(10):975-983. [doi: 10.2174/1567205015666180427124547]

Bauernschmidt D, Hirt J, Langer G, et al. Technology-based counselling for people with dementia and their informal carers: a systematic review and meta-analysis. J Alzheimers Dis. 2023;93(3):891-906. [doi: 10.3233/JAD-221194] [Medline: 37125549]

Mao W, Qi X, Chi I, Wichinsky L, Wu B. Technology-based interventions to address social isolation and loneliness among informal dementia caregivers: a scoping review. J Am Med Dir Assoc. Nov 2023;24(11):1700-1707. [doi: 10.1016/j.jamda.2023.08.005] [Medline: 37678415]

Zhu A, Cao W, Zhou Y, Xie A, Cheng Y, Chu SF. Tele-health intervention for carers of dementia patients-a systematic review and meta-analysis of randomized controlled trials. Front Aging Neurosci. 2021;13:612404. [doi: 10.3389/fnagi.2021.612404] [Medline: 33643022]

Carotenuto A, Traini E, Fasanaro AM, Battineni G, Amenta F. Tele-neuropsychological assessment of Alzheimer’s disease. J Pers Med. Jul 21, 2021;11(8):688. [doi: 10.3390/jpm11080688] [Medline: 34442332]

Ferreira Santana R, Vaqueiro Dantas R, et al. Telecare to elderly people with Alzheimer and their caregivers: systematic review. Cienc Cuid Saude. 2018;17:1-6. [doi: 10.4025/cienccuidsaude.v17i4.41653]

Söylemez BA, Özgül E, Küçükgüçlü Ö, Yener G. Telehealth applications used for self-efficacy levels of family caregivers for individuals with dementia: a systematic review and Meta-analysis. Geriatr Nurs (Lond). Jan 2023;49:178-192. [doi: 10.1016/j.gerinurse.2022.12.001]

Washington SE, Bollinger RM, Edwards E, McGowan L, Stephens S. Telehealth delivery of evidence-based intervention within older adult populations: a scoping review. OTJR (Thorofare N J). Jul 2023;43(3):467-477. [doi: 10.1177/15394492231180838] [Medline: 37322873]

Graven LJ, Glueckauf RL, Regal RA, Merbitz NK, Lustria MLA, James BA. Telehealth interventions for family caregivers of persons with chronic health conditions: a systematic review of randomized controlled trials. Int J Telemed Appl. 2021;2021:3518050. [doi: 10.1155/2021/3518050] [Medline: 34093704]

Yi JS, Pittman CA, Price CL, Nieman CL, Oh ES. Telemedicine and dementia care: a systematic review of barriers and facilitators. J Am Med Dir Assoc. Jul 2021;22(7):1396-1402. [doi: 10.1016/j.jamda.2021.03.015] [Medline: 33887231]

Sekhon H, Sekhon K, Launay C, et al. Telemedicine and the rural dementia population: a systematic review. Maturitas. Jan 2021;143:105-114. [doi: 10.1016/j.maturitas.2020.09.001] [Medline: 33308615]

León-Salas B, González-Hernández Y, Infante-Ventura D, et al. Telemedicine for neurological diseases: a systematic review and meta-analysis. Eur J Neurol. Jan 2023;30(1):241-254. [doi: 10.1111/ene.15599] [Medline: 36256522]

Folder N, Power E, Rietdijk R, Christensen I, Togher L, Parker D. The effectiveness and characteristics of communication partner training programs for families of people with dementia: a systematic review. Gerontologist. Apr 1, 2024;64(4):gnad095. [doi: 10.1093/geront/gnad095] [Medline: 37439771]

Yu Y, Xiao L, Ullah S, et al. The effectiveness of internet-based psychoeducation programs for caregivers of people living with dementia: a systematic review and meta-analysis. Aging Ment Health. 2023;27(10):1895-1911. [doi: 10.1080/13607863.2023.2190082] [Medline: 36951611]

Hailey D, Roine R, Ohinmaa A. The effectiveness of telemental health applications: a review. Can J Psychiatry. Nov 2008;53(11):769-778. [doi: 10.1177/070674370805301109] [Medline: 19087471]

Lucero RJ, Fehlberg EA, Patel AGM, et al. The effects of information and communication technologies on informal caregivers of persons living with dementia: a systematic review. Alzheimers Dement (N Y). 2018;5:1-12. [doi: 10.1016/j.trci.2018.11.003] [Medline: 30623020]

Rueda Daz LJ, Monteiro da Cruz DL. The efficacy of telephone use to assist and improve the wellbeing of family caregivers of persons with chronic diseases: a systematic review. JBI Database System Rev Implement Rep. Dec 2014;12(12):106-140. [doi: 10.11124/jbisrir-2014-1566]

Nkodo JA, Gana W, Debacq C, et al. The role of telemedicine in the management of the behavioral and psychological symptoms of dementia: a systematic review. Am J Geriatr Psychiatry. Oct 2022;30(10):1135-1150. [doi: 10.1016/j.jagp.2022.01.013] [Medline: 35241355]

Liang J, Aranda MP. The use of telehealth among people living with dementia-caregiver dyads during the COVID-19 pandemic: scoping review. J Med Internet Res. May 25, 2023;25:e45045. [doi: 10.2196/45045] [Medline: 37227755]

Armstrong MJ, Alliance S. Virtual support groups for informal caregivers of individuals with dementia: a scoping review. Alzheimer Dis Assoc Disord. 2019;33(4):362-369. [doi: 10.1097/WAD.0000000000000349] [Medline: 31567144]

Wood M, Walshe C, McCullagh A. What are the digitally enabled psychosocial interventions delivered by trained practitioners being offered to adults with life-shortening illnesses and palliative care needs and their informal and professional caregivers? A scoping review. Palliat Support Care. Aug 2023;21(4):727-740. [doi: 10.1017/S1478951523000172] [Medline: 36994819]

Sun Y, Ji M, Leng M, Wang Z. Which cognitive behavioral therapy delivery formats work for depressive symptoms in dementia caregivers? - A systematic review and network meta-analysis of randomized controlled trials. J Affect Disord. Jul 1, 2022;308:181-187. [doi: 10.1016/j.jad.2022.04.055] [Medline: 35429541]

Kishita N, Hammond L, Dietrich CM, Mioshi E. Which interventions work for dementia family carers?: an updated systematic review of randomized controlled trials of carer interventions. Int Psychogeriatr. Nov 2018;30(11):1679-1696. [doi: 10.1017/S1041610218000947] [Medline: 30017008]
